# Thank you to Reviewers 2021

**DOI:** 10.1002/cam4.4695

**Published:** 2022-05-03

The Editor‐in‐Chief, the co‐Editors, and the Editorial team gratefully acknowledge the below individuals who served as peer reviewers for *Cancer Medicine* during 2021.

Aacharya, Srijan

Aakre, Jeremiah

Aapro, Matti

Aaronson, Neil K.

Aarts, Anne

Aasebø, Kristine

Abalo, Raquel

Abate, Massimo

Abbas, Kirellos

Abbasi, Wajid Arshad

Abbaszadegan, Mohammad Reza

Abbotoy, Daniel

Abbott, Tom E. F.

Abd El‐Aziz, Ayman Hassan

Abd Elnaser, Anwar

Abdel‐Daim, Mohamed

Abdelhakiem, Mohamed

Abdellatif, Ahmed

Abdelmohsen, Gaser

Abdel‐Naim, Ashraf B.

Abdel‐Rahman, Omar

Abdel‐Rahman, Omar

Abdel‐Razeq, Hikmat

Abderrahman, Balkees

Abdessater, Maher

Abdolahad, Mohammad

Abdollahi, Pegah

Abdollahpour‐Alitappeh, Meghdad

Abdou, Yara

Abdulhaq, Haifaa

Abdulkarim, Bassam S.

Abdullah, Khatijah

Abdulwadoud Alshoabi, Sultan

Abe, Jiro

Abe, Keiko

Abe, Takanori

Abeling, Nico

Abenavoli, Ludovico

Aberger, Fritz

Abgottspon, Stephanie

Abi‐Raad, Rita

Ablin, Richard

Abnet, Christian

Abnet, Christian C.

Abobaker Al‐Ghanimi, Ibrahim

Aboelsoud, Mohammed

Aboulafia, David

Aboussekhra, Abdelilah

Abraham, Jame

Abraham, Jean E.

Abraham, Philip

Abrahao, Renata

Abrams, Thomas

Abramson, Jeremy S.

Abreu, Miguel Henriques

Abruzzese, Elisabetta

Abruzzi, Amy

Abualkhair, Wesal

Abubakar, Mustapha

Abufaraj, Mohammad

Abuhelwa, Ahmad

Abukhdeir, Abde

Abulizi, Guzhalinuer

Abushukair, Hassan

Accardi, Luisa

Accordino, Melissa

Acharya, Jay

Achebe, Ikechukwu

Acquah, George

Acuna, Sergio A.

Acuna‐Villaorduna, Ana

Adachi, Souichi

Adam, JF

Adam, Maya

Adam, Rosalind

Adam, Salome

Adam, Vojtech

Adambekov, Shalkar

Adami, Guy

Adami, Hans‐Olav

Adamkov, Marian

Adamo, Vincenzo

Adams, Brian

Adams, F.

Adams, Hugo J. A.

Adams, Kumari N.

Adams, Richard

Adams‐Campbell, Lucile

Adamu, Danladi

Adashek, Jacob

Addeo, Alfredo

Addeo, Raffaele

Addissie, Adamu

Adedeji, Isaac

Adelborg, Kasper

Aden, Konrad

Adjei Boakye, Eric

Adjei, Araba

Adrian, Thomas E.

Adusumalli, Srinath

Advani, Anjali

Aerts, Hugo J. W. L.

Aerts, Joachim

Afzali Deligani, Maryam

Agaku, Israel

Agalar, Cihan

Agalliu, Ilir

Agarwal, Charu

Agarwal, Ekta

Agarwal, Neeraj

Agarwal, Ritesh

Agarwal, Shailesh

Aggarwal, Ajay

Aggarwal, Bharat B.

Aggerholm‐Pedersen, Ninna

Aghdaei, H. Asadzadeh

Aglago, Elom

Aglietta, Massimo

Agnelli, Giancarlo

Agnelli, Luca

Agolli, Linda

Agoritsas, Thomas

Agostinis, Chiara

Agrawal, Tarjani

Aguiar, Pedro

Agulnik, Asya

Agulnik, Mark

Ahadi, Ali Mohammad

Ahadova, Aysel

Ahangari, G.

Ahles, Tim

Ahmad, Aamir

Ahmad, Sarfraz

Ahmad, Syed A.

Ahmad, Tessnim

Ahmadi, Mohsen

Ahmadzadehfar, Hojjat

Ahmed, Nuzhat

Ahmed, Sairah

Ahmed, Sayem

Ahmed, Wafaa

Ahn, Dongbin

Ahn, Hanjong

Ahn, Kwang Seok

Ahn, Myung‐Ju

Ahn, Sang‐Hoon

Ahn, Sei‐Hyun

Ahn, Sung‐Ja

Ahn, Sungsoo

Ahn, Yong Chan

Ai, Xiang

Ai, Xiaoming

Ai, Yanqiu

Ai, Zisheng

Aigner, Karl

Ailawadhi, Sikander

Aisner, Dara L.

Aizawa, Rihito

Aizer, Ayal A.

Ajani, J. A.

Ajisawa, Atsushi

Akagi, Yoshito

Akahane, Koshi

Akaike, Tomoko

Akaishi, Ryujiro

Akamine, Takaki

Akane, Kiyoko

Akao, Yukihiro

Akay, Ali

Akbani, Rehan

Akbari, Abolfazl

Akce, Mehmet

Akdemir, Kadir C.

Akechi, Tatsuo

Akerley, Wallace

Akhavanfard, Sara

Akhavan‐Niaki, Haleh

Akhmedov, Mobil

Akhtar, Razia

Akimoto, Tetsuo

Akin, Semiha

Akinmade, Akinola

Akinyemiju, Tomi

Akiyama, Yasuto

Akiyoshi, Takashi

Aksoy, Asude

Akter, Mst. Farzana

Al Achkar, Morhaf

Al Assaad, Majd

Ala, Moein

Al‐Abd, Ahmed M.

Alabi, Rasheed Omobolaji

Alagoz, Oguzhan

Alagpulinsa, David

Alagpulinsa, David A.

Alahmari, Ali

Alajez, Nehad M.

Alajez, Nehad

Alamoudi, Hanin

Alani, Rhoda

Alatrash, Gheath

Al‐Badawi, Ismail

Albalawi, Lina

Albanes, Demetrius

Albanes, Demitrius

Albano, Domenico

Albert, Adelin

Albi, Elisabetta

Albiges, Laurence

Albritton, Karen

Albuquerque, Kevin

Alcoceba, Miguel

Al‐Danakh, Abdullah

Alderuccio, Juan

Al‐Dewik, Nader

Al‐Dhabi, mokhtar

Aldoss, Ibrahim

Alegria, Margarita

Alektiar, Kaled M.

Alemany, Laia

Alenezi, Abdulraham

Alese, Olatunji

Aleskandarany, Mohammed A.

Alessio, Massimo

Alexander, Erik

Alexander, Koshy

Alexander, Mariam

Alexandrov, Ludmil

AlFaar, Ahmad

Alfieri, Sara

Alford, Simone

Algazi, Alain

Alhalabi, Omar

Al‐Haseni, Ali

Ali, Askal A.

Ali, Bahadori

Ali, J. M.

Ali, Mustafa

Ali, Navaira

Ali, Raghib

Ali, Siraj M.

Ali, Syed Z.

Alibhai, Shabbir

Alimoglu, O.

Alinejad‐Rokny, Hamid

Alix‐Panabieres, Catherine

Al‐Jabi, Samah

Al‐Kufaishi, Ali

Allame, Farzad

Allavena, Paola

Allcock, Richard

Allegra, Carmen

Allegra, Eugenia

Allegra, Sarah

Allegrucci, Cinzia

Allemani, Claudia

Allen, Antino

Allen, Naomi

Allen, Upton

Allodji, Rodrigue

Allott, Emma

Almangush, Alhadi

Almassinokiani, Fariba

Almeida Parra, Julia

Almeida, Rodrigo Coutinho de

Almouzni, Geneviève

Almutairi, Abdulaali

Al‐Omari, Amal

Alongi, Pierpaolo

Alonso, Jordi

Alonso, Sergio

Al‐Rawashdeh, Nedal

Alshaker, Heba

Alshammari, Mukhlid

Al‐Shamsi, Humaid O.

Altan, Mehmet

Altekruse, Sean

Al‐Tell, Abdallah

Alter, Blanche

Altomonte, Jennifer

Altorki, Nasser K.

Al‐Toubah, Taymeyah

Alumkal, Joshi J.

Alvarez, Clara

Alvarez, Elysia M.

Alvarez‐Fernandez, Sheila Maria

Alvarez‐Larran, Alberto

Álvarez‐Maestro, M.

Alves dos Santos, Camila

Alves, Carla L.

Alves, Mariana

Alwan, Heba

Alzahrani, Anas

Amaadour, Lamiae

Amani, Ali Mohammad

Amankwah, Ernest

Amaral Mendes, Rui

Amaravadi, Ravi K.

Amatore, Florent

Ambulkar, Reshma

Ameri, Pietro

Amidi, Ali

Amin Al Olama, Ali

Amin, Asim

Aminawung, Jenerius

Amini, Arya

Amir, Amnon

Amir, Eitan

Amoli, Mahsa

Amos, Christopher

Amrane, Karim

Amro, Elias

Amrutkar, Manoj

Amundadottir, Laufey

An, Chao

An, Lawrence

An, Sanqi

An, Thuy

An, Xin

An, Yi

Anampa, Jesus

Anand, Kartik

Anand, Shashi

Anand, Vivek

Anastasov, Natasa

Anazodo, Antoinette

Andersen, Klaus

Andersen, Vibeke

Anderson, Chelsea

Anderson, Geoff

Anderson, Janeane

Anderson, John

Anderson, Megan

Anderson, Peter

Anderson, Roger

Andersson, Yvonne

Andl, Claudia

Ando, Taiki

Ando, Yuichi

Andoh, Akira

Andoh, Shohei

Andre, Thierry

Andreassen, Bettina

Andrechek, Eran

Andresen, Carolin

Andrews, Miles C.

Andriani, Lisa

Andrieu, Nathalie

Angajala, Anusha

Angel Diaz, Miguel

Angelini, Sabrina

Angelique, Gougelet

Angell, Trevor

Angus, Lindsay

Ankathil, Ravindran

Ankerst, Donna P.

Anna, Pessino

Annibali, Daniela

Annibali, Ombretta

Annilo, Tarmo

Annunziata, Salvatore

Anoff, Debra

Ansari, Kausar

Ansari, Noureddin Nakhostin

Ansell, Stephen

Antman, Elliott M.

Antonelli, Alessandro

Antonelli, Giulio

Antonelli, Guido

Antonescu, Cristina

Antoniewicz, Maciek R.

Antonsson, Annika

Anttila, Ahti

Antunes, Camila

Antunes, Luis

Antwi, Samuel

Aoe, Jo

Aoe, Keisuke

Aoki, Manabu

Aoki, Masahiko

Aoyama, Toru

Apolo, Andrea

Appelbaum, Fred

Appelt, Ane

Appendino, Giovanni

Apperley, Jane

Apple, Alexandra

Applebaum, Allison J.

Aragane, Naoko

Aragon‐Ching, Jeanny

Arai, Nana

Arai, Yuki

Arakawa, Yoshiki

Arandi, Nargess

Arandjelovic, Ognjen

Aras, Glfidan

Araujo, Daniel

Araujo‐Castro, Marta

Arbyn, Marc

Arcaini, Luca

Archer, Stephanie

Arciero, Cletus

Arem, Hannah

Arenas, Alicia

Arend, Rebecca C.

Arends, Jann

Aretz, Stefan

Arfuso, Frank

Arga, Kazim Yalcin

Argenta, Peter

Argiles Huguet, Jose Maria

Arians, Nathalie

Arias, Anita

Arima, Yoshimi

Arisaka, Yukiko

Arivazhagan, A.

Ariza‐Heredia, Ella

Arlt, A.

Armaroli, Paola

Armenian, Saro

Armirotti, Andrea

Armitage, James

Armstrong, Andrew J.

Armstrong, Gregory

Armstrong, Katrina

Arnelo, Urban

Arnold, Jonathon

Arnold, Lauren

Arnold, Melina

Aro, Karoliina

Arodz, Tomasz

Aron, Monish

Aronson, William

Arora, M.

Arraras, Juan

Arredondo, Jorge

Arrington, John A.

Arteaga, Carlos L.

Arthur, R.

Arthurs, Erin

Artigalás, Osvaldo

Artyomov, Maxim N.

Arun, Banu

Arunachalam, Ashwini

Asadi, Malek Hossein

Asafu‐Adjei, Denise

Asamoah, Francis

Asamura, Hisao

Asano, Hiroshi

Asano, Kenichiro

Asano, Masatake

Asano, Naoko

Asaoka, Raito

Asati, Vikas

Asato, Ryo

Asbach, Patrick

Ascha, Mustafa

Ascierto, Paolo

Ascierto, Paolo A.

Ascoli, Valeria

Asdahl, Peter

Asgary, Ramin

Asghar, Waseem

Ashihara, Eishi

Ashok, Shaha

Ashourpour, Mahboubeh

Ashton‐Prolla, Patricia

Ashur‐Fabian, Osnat

Askar, Mostafa

Aspinall, Sherrie L.

Assi, Hazem

Atabey, Nese

Atagi, Shinji

Atallah, Ehab

Atanasov, Georgi

Atenafu, Eshetu

Atkins, Michael

Atkinson, M.J.

Atoui, Ali

Atrash, Shebli

Attoub, Samir

Atwal, Dinesh

Au Yeung, Shiu Lun

Auberger, Patrick

Audenet, Francois

Auguste, Aviane

Augusto Real Martinez, Carlos

Aukema, Tjeerd S.

Auliac, jean bernard

Aune, Dagfinn

Aung, Winn

Aushev, Vasily

Autorino, Riccardo

Auvinen, Eeva

Avčin, Tadej

Avigan, David

Aviki, Emeline

Avtanski, Dimiter

Awan, Farrukh

Awasthi, Shivanshu

Awick, Elizabeth A.

Awlia, Alaa

Ayanian, John Z.

Ayanlaja, Abiola

Ayaz, Mazhar

Aydin, Funda

Aytekin, Aydin

Azadnajafabad, Sina

Azale, Telake

Aziz, Mohammad Azhar

Azizi‐Tabesh, Ghasem

Azkona, Eider

Azoulay, David

Azoulay, Laurent

Azuma, Koichi

Azzi, Jacques

Ba, Yi

Baade, Peter

Baba, Hideo

BaBa, Shingo

Baba, Yoshifumi

Babei, Masoud

Babiera, Gildy

Babiker, Hani

Baccarani, Michele

Bach, Peter

Bachy, Emmanuel

Back, Anthony

Back, Michael

Badami, Sunil

Badani, Ketan

Badgwell, Brian

Badrising, Sushil

Bae, Ja Seong

Baek, Jung Hwan

Baena‐Cañada, José

Baenziger, Julia

Baez‐Ortega, Adrian

Bafandeh Zendeh, Mostafa

Bagheri, Nader

Bahadoran, Zahra

Bai, Chunmei

Bai, Fan

Bai, Fumao

Bai, Han

Bai, Hua

Bai, Jinbing

Bai, Jinfeng

Bai, Li‐Yuan

Bai, Tao

Bai, Xiaoyu

Bai, Xue

Bai, Xueli

Bai, Yidong

Bai, Yongrui

Baichoo, Shakuntala

Bail, Jennifer

Bainbridge, Matthew

Baiocchi, Gian Luca

Baiocchi, Glauco

Bajaj, Rajat

Baker, Allison

Baker, Justin

Baker, Laurence

Bakhshandeh, Haleh

Bakitas, Marie

Bakouny, Ziad

Bakre, Manjiri

Bakshi, Sameer

Balasubramanian, Poonkuzhali

Balasubramanian, Sriram

Balboni, Tracy A.

Balcacer, Patricia

Baldacci, Simon

Baldari, Sergio

Baldauf, Jean‐Jacques

Baldini, Capucine

Baldini, E.

Baldo, Cristiani

Baldwin, Mike

Balermpas, Panagiotis

Baljevic, Muhamed

Balkrishnan, Rajesh

Balkwill, Fran

Ballas, Leslie

Balmes, John

Baloch, Zubair W.

Baloch, Zulqarnain

Bamias, Aristotelis

Bamodu, Oluwaseun Adebayo

Banchs, Jose

Bandak, Mikkel

Bandapalli, Obul

bandini, marco

Bandini, Marco

Bandla, Aishwarya

Banegas, Matthew P.

Banerjee, S. K.

Bang, Yeonghak

Bang, Yung Jue

Bang, Yung‐Jue

Bange, Erin

Bangmin, Han

Bannon, Finian

Bansal‐Travers, Maansi

Bao, Bo‐Ying

Bao, Huijing

Bao, Ting

Bao, Yi

Bao, Zhaoshi

Barabe, Frederic

Barabutis, Nektarios

Baracos, Vickie

Baradaran, Behzad

Barata, Pedro

Baratloo, Alireza

Baratto, Lucia

Barba, Maddalena

Barbagallo, Cristina

Barbaret, Cecile

Barber, Kimberly

Barbera, Lisa

Barbieri, Antonio

Barbosa‐Morais, Nuno

Barbour, Andrew

Barcenas, Carlos

Barchowsky, Aaron

Barciszewska, Anna‐Maria

Barco, Stefano

Bardia, A.

Bareja, Rohan

Barkauskas, Donald

Barlev, Nickolai A.

Barnaghi, Payam

Barnes, Benjamin

Barnes, Geoffrey D.

Barni, Sandro

Barnie, Prince Amoah

Baron, Frederic

Baron, Frédéric

Baroni, Ronaldo H.

Barr, Frederic

Barr, Paul

Barranco Lampón, Gilberto Israel

Barrett, Donal

Barrett, Nadine

Barrett, Tyler

Barrington, Robert

Barrios, Carlos Henrique

Barron, Thomas

Barros, Bruna

Barros, Marilisia

Barroso‐Sousa, Romualdo

Bartelink, Harry

Bartels, Stephan

Bartlett, Nancy L.

Bartoletti, Daniela

Bartoszewski, Rafal

Bartula, Iris

Barua, Ishita

Barucci, A.

Barzi, Afsaneh

Barzilai‐Birenboim, Shlomit

Basch, Ethan

Basha, Mohammad Abd Alkhalik

Bashir, Nasir

Bashir, Qaiser

Basho, Reva

Basile, Umberto

Basolo, Fulvio

Basra, MuHammad Asim Raza

Bassan, Renato

Basset‐Seguin, Nicole

Bassetti, Michael

Bassi, Claudio

Bast, Robert

Bastami, Milad

Bastola, Dhundy R.

Basu, Anirban

Basu, Partha

Basu‐Roy, Upal

Batista, Alzir A.

Batra, Atul

Batra, Jyotsna

Batra, Surinder K.

Battista, Serena

Battisti, Nicolò

Batura, Deepak

Baudry, Anne‐Sophie

Bauer, Thomas L.

Bauersachs, Rupert

Baugh, Christopher W.

Baulande, Sylvain

Bauman, Jessica

Baumann, Brian

Baumeister, Philipp

Baumert, Brigitta G.

Baumstarck, Karine

Baussano, Iacopo

Baust, Katja

Bausys, Augustinas

Bautista, Francisco Jose

Baxi, Shrujal

Baxter, Nancy

Baxter, Robert C.

Bayar, Goksel

Baydogan, Seyda

Bayih, Wubet Alebachew

Baylin, Syephen

Baymon, Damarcus

Baysal, Bora E.

Baz, Rachid

Bazan, Jose

Bazarbashi, Shouki

Bazzi, Angela R.

Beachler, Daniel

Beale, David

Beasley, Mary Beth

Beau, Anna‐Belle

Beaubrun‐Renard, Murielle

Beaulieu, Jean‐François

Beaumont, Jennifer

Beaver, Julie

Beck, Alain

Becker, Daniel J.

Becker, Heiko

Becker, Jurgen

Beckwith, Heather

Bedada, Nagasa Dida

Bedard, Angela

Bedke, Jens

Beebe‐Dimmer, Jennifer

Beekman, Aartjan T. F.

Been, Lukas

Beernaert, Kim

Beets‐Tan, Regina

Beg, Shaham

Beggs, Andrew

Beharee, Nitish

Behring, Michael

Bei, Jin‐Xin

Beije, N.

Beisland, Christian

Bejide, Ronald

Bekaii‐Saab, Tanios

Bekelman, Justin

Bel, A.

Belani, Chandra

Belch, Andrew

Belfer, Inna

Belibasakis, Georgios

Belka, Claus

Belkora, Jeffrey

Bell, Christopher

Bell, Jennifer

Bellavance, Emily

Belliere, Julie

Bellini, Edoardo

Bellis, Susan L.

Bello, Ibrahim

Bellon, Jennifer

Bellone, Matteo

Belotti, Angelo

Beltrán, Angela

Benacerraf, B. R.

Ben‐Aharon, Irit

Benard, Vicki

Bence, Alta

Benderra, Marc‐Antoine

Ben‐Eliyahu, Shamgar

Benetatos, Leonidas

Benezery, Karen

Bengel, Jürgen

Benitez, Javier

Benjamin, Robert

Bennedsgaard, Kristine

Bennett, Antonia V.

Bennett, Derrick A.

Bennett, John

Bensalah, Karim

Bensenor, Isabela M.

Benson, Al

Benson, Charlotte

Benson, Jacqueline

Benson, Sven

Bentrem, David

Bentrem, David J.

Benusiglio, Patrick

Benz, Rudolf

Beppu, Shintaro

Bera, K.

Berano Teh, Jennifer

Berardi, Rossana

Berbari, Elie F.

Berchuck, Jacob

Berciano‐Guerrero, Miguel‐Angel

Berg, Carla J.

Berger, Ann

Berger, Barry M.

Berger, Nathan

Bergerot, Cristiane

Bergh, Anders

Bergin, Rebecca

Bergmann, Lothar

Bergmark, Regan

Bergquist, Henrik

Bergquist, John

Bergqvist, Jenny

Bergsland, Emily

Berindan‐Neagoe, I.

Beriwal, Sushil

Berkhof, Johannes

Berkman, Amy

Berman, Benjamin P.

Bernabe, Eduardo

Bernard, Stephen

Bernard, Virginie

Bernardi, Simona

Bernard‐Pierrot, Isabelle

Bernards, René

Berndt, Nikolaus

Bernhardt, Denise

Bernhart, Stephan

Bernhart, Stefan

Bernt, Kathrin M.

Berrigan, David

Berruti, Alfredo

Berthold, Frank

Bertolaccini, Luca

Bertoli, Sarah

Bertolo, Riccardo

Berton, Stefania

Bertoni, Francesco

Bertram, Ceyda

Bertrand, Kim

Berwick, Mariann

Besmer, P.

Besselink, Dr. M.G.H.

Bethea, Traci

Beutler, Andreas

Beutler, Andreas S.

Bex, Axel

Bexiga, Catarina

Beynon, Christopher

Bhak, Rachel

Bhandarkar, Nikhil

Bhardwaj, Nina

Bhat, Ambarish P.

Bhat, Mohammad

Bhatelia, Khyati

Bhatia, Aarti

Bhatia, Ankush

Bhatia, Shailender

Bhatia, Smita

Bhatla, Neerja

Bhatnagar, Sushma

Bhatt, Madan Lal

Bhattacharjee, Anukana

Bhattacharya, Resham

Bhattacharya, Rituparna

Bhattacharyya, Arinjita

Bhojwani, Deepa

Bhowmick, Debajit

Bhujwalla, Zaver M.

Bhuket, Taft

Bi, Guoli

Bi, Rui

Bi, Zhuofei

Bian, Jifeng

Bian, Shelly

Bian, Shelly X.

Bian, Xiuhua

Bian, Xiu‐Hua

Bian, Xiu‐wu

Bian, Yiying

Bian, Yuan

Bian, Yun

Bian, Zehua

Bianchi, Paolo

Bianci, Pedro

Bianco, Roberto

Bianconi, Francesco

Biau, J.

Bibault, Jean‐Emmanuel

bibeau, frederic

Bibi, Rashida

Biccler, Jorne

Bidard, Francois‐Clement

Bidoli, Paolo

Bidram, Maryam

Bidstrup, Pernille

Bieber, Christiane

Biering, Karin

Biester, Torben

Bikle, Daniel

Bilal, Muhammad

Bilani, Nadeem

Bilen, Mehmet

Bilen, Mehmet A.

Bilger, Angelika

Bilheem, Surichai

Biliatis, Ioannis

Bilici, Ahmet

Biller, Armin

Biller, Leah

Billingham, Lucinda

Bin, Yuan Le

Binder, Hans

Bini, Federica

Binswanger, Ingrid A.

Biondi, Alberto

Biran, Noa

Birchmeier, Walter

Birmaher, Boris

Biron, Vincent

Bish, Rebecca

Bishara, Jihad

Bishop, David Timothy

Bisoffi, Marco

Biswal, Bijesh Kumar

Biswal, Shyam S.

Biswas, Bivas

Bitencourt, Almir

Bjartell, Anders

Bjelic‐Radisic, Vesna

Bjerkreim, Anna

Bjorge, Tone

Black, Peter

Black, Peter C.

Blackstone, Eugene H.

Blair, Steven N.

Blake, Kelly

Blake, Paul

Blanchard, Pierre

Blanck, Oliver

Blandino, Giovanni

Blank, Stephanie

Blay, Jy

Blazeby, Jane

Blazer, Marlo

Bleijenberg, Gijs

Bleile, MaryLena

Bleyer, Archie

Blinder, Victoria

Bloch, Orin

Bloemers, Jos

Blomqvist, Carl

Bluethmann, Shelley

Blum, Joanne

Blum, Mariela

Blumenthal, Deborah T.

Blumenthal, Gideon M.

Bober, Sharon L.

Bobic Rasonja, Mihaela

Boccardo, Enrique

Boccia, Ralph

Bocharova, Iryna

Bock, C.‐Thomas

Bock, Cathryn

Boddouhi, Bahram

Bode, Leticia

Bodelon, Clara

Bodilsen, Jacob

Boeck, Stefan

Boehmer, Leigh

Boeing, Heiner

Boer, Judith

Boer, Monique L. Den

Boers‐Doets, Christine

Boffa, Daniel

Boffetta, Paolo

Bogaards, Hans

Bogowicz, Marta

Boilly, Benoni

Boise, Lawrence

Boissel, Nicolas

Boland, Angela

Boldrini, Luca

Bolin, Liu

Bolland, Fiona

Bollschweiler, Elfriede

Bolton, Damien

Boman, Krister

Bombard, Yvonne

Bonanno, Laura

Bond, Stewart

Bonde, Anders N.

Bonde, Jesper

Bonetto, Andrea

Bonewald, Lynda

Bongiovanni, Massimo

Boni, Christian

Bonifacio, Massimiliano

Bonin, Serena

Bonnefoy, Nathalie

Bonnema, Steen

Bonomi, Philip

Bonvin, Alexandre M. J. J.

Bood, Zarah

Booka, Eisuke

Boot, Annemieke

Booth, Chris

Booth, Christine N.

Borad, Mitesh

Borazanci, Erkut

Borges do Nascimento, Israel Júnior

Borghesi, Andrea

Borjan, Jovan

Borlak, Jürgen

Borniger, Jeremy C.

Borno, Hala

Bornschein, Jan

Boros, Adriana

Borowski, D. W.

Borre, Michael

Borrello, Maria Grazia

Borse, Vikrant

Borsellino, Nicolò

Borthakur, Gautam

Borzoueisileh, Sajad

Bos, Monique

Bosch, Francesc

Bosch, T. Van den

Bosdriesz, Evert

Bosenberg, Marcus W.

Bosetti, Christina

Bosma, Madeleen

Bosma, Nicholas

Bosse, Tjalling

Bossis, Guillaume

Bossuyt, Patrick

Bossuyt, Patrick M. M.

Botta, Laura

Botteman, MF

Botter, Sander M.

Botticelli, Andrea

Bottinor, Wendy

Botto, Rossana

Bouabdallah, Reda

Bouberhan, Sara

Boucai, Laura

Bouchart, Christelle

Bouchez, Clémentine

Bou‐Dargham, Mayassa

Bouffet, Eric

Bougas, nicolas

Boughey, Judy

Boukheris, Houda

Bourhis, Jean

Bourke, Liam

Bourmaud, Aurélie

Bourne, Tom

Bourquin, Jean‐Pierre

Bours, Martijn

Boussion, Hélène

Bouvet, Michael

Bouvier, Veronique

Bowler, Scott

Bowles, Daniel

Boye, Kjetil

Boyer, Michael

Boyers, Dwayne

Boyle, Theresa

Boyraz, Gokhan

Boytcheva, Svetla

Bozkaya, Yakup

Bozzetti, Federico

Bracci, Paige M.

Bradbury, Penelope A.

Brade, Anthony M.

Bradford, Andrea

Bradley, Cathy

Bradshaw, Patrick

Braga, Sonia

Brahmbhatt, Rushin

Brahmer, Julie R.

Braicu, Elena Ioana

Brain, Etienne

Brajuskovic, G.

Brakenhoff, Ruud

Branagan, Andrew

Branciforte, Bruno

Brandi, Giovanni

Brandt, Heather

Brasier‐Lutz, Pascale

Brasme, Jean‐François

Brassil, Kelly

Brasso, Claus

Braun, Ilana

Braunstein, Lior

Braunwald, Eugene

Bravi, Francesca

Bravo, Ignacio

Bray, Freddie

Braybrooke, Jeremy

Brayer, Jason

Brazil, Kevin

Breccia, Massimo

Brecht, Ines

Brehmer, Marianne

Brenes, Maria

Brennan, Paul

Brenner, Benjamin

Brenner, Hermann

Brenner, Michael

Brentjens, Renier

Bresson, Catherine

Brest, Patrick

Bretthauer, Michael

Brevi, Arianna

Brevik, Thea

Bria, Emilio

Bridgewater, John

Bridle, Byram W.

Briganti, Alberto

Brillanti, Stefano

Brillet, Pierre Yves

Brinkman, Tara M.

Brinton, Louise A.

Bristow, Robert E.

Britton, Heidi

Broaddus, Russell

Brock, Katharine

Brockmeier, Ulf

Broder, Michael

Brodszky, V.

Broek, Frank

Brooks, Gabriel

Broom, Alex

Brotkin, Samuel

Brouwers, Melissa

Brown, Austin

Brown, Joshua

Brown, Joshua D.

Brown, Kevin

Brown, Sherry Ann

Brown, Simon

Brown, Taurean

Brown, Thomas

Brown‐Glaberman, Ursa

Brownstein, Naomi

Bruera, Eduardo

Brufsky, Adam

Brugarolas, James

Brugge, Joan S.

Bruix, Jordi

Bruna, Jordi

Brundage, Michael

Brunello, Antonella

Bruner, Deborah Watkins

Brunetto, Maurizia Rossana

Brungs, Daniel

Brusselaers, Nele

Bryere, Josephine

Bu, Ning

Bu, Shizhong

Bu, Wenbo

Bu, Yan‐Zhi

Buas, Matthew

Bubendorf, Lukas

Bubnoff, Dagmar

Buch, Kristian

Buchanan, Daniel

Buchbinder, Elizabeth

Buchler, Tomas

Buchsbaum, Donald J.

Buckanovitch, Ronald

Buckhaults, Phillip

Buckland, Genevieve

Bucknall, Tracey

Buckstein, Michael

Buckstein, Rena

Buendia, Marie Annick

Bueni‐Martinez, Elena

Buergy, Daniel

Buffart, Laurien

Bugajski, Marek

Buijs, S.B.

Buijsen, Jeroen

Bukowinski, Andrew

Bull, Caroline

Bull, Caroline F.

Bulliard, Jean‐Luc

Bullinger, Lars

Bundhamcharoen, Kanitta

Bunduc, Stefania

Bunnell, Rebecca E.

Burbano, Rommel

Burbos, Nikolaos

Burchill, Susan A.

Burdett, Sarah

Burge, Fred

Burger, Jan A.

Burgess, Andrew

Burgess, Melissa

Burillo‐Sanz, Sergio

Buring, Julie E.

Burn, John

Burnett, Alan K.

Burnett, Richard T.

Burnett‐Hartman, Andrea

Burns, Michael

Burnside, Elizabeth S.

Burnside, Melissa

Buron, Andrea

Burotto, Mauricio

Burt, Morton

Burtness, Barbara

Burton‐Chase, Allison

Buscariollo, Daniela

Busch, Susan

Busco, Susanna

Buskermolen, Maaike

Butow, Phyllis

Buwenge, Milly

Buyru, Nur

Byeon, Seonggyu

Byers, Lauren

Byrd, Kenneth

Byrne, Margaret

Byun, Eeeseung

Byun, Jinyoung

Byun, Kyung Do

Byun, Seok‐Soo

Caan, Bette

Caballero, AC

Caballero‐Diaz, Daniel

Cabanillas, Maria

Cabel, Luc

Cabibbo, Giuseppe

Cabrera‐Díaz, Silvia

Cacan, Ercan

Cacheux, Wulfran

Cacioppo, Cara

Cadet, Tamara

Cadranel, Jacques

Caetano, Patricia

Caffo, Orazio

Cagini, Lucio

Cagney, Daniel

Cahill, Declan

Cahill, Dolores

Cai, Bo

Cai, Daxia

Cai, Gengming

Cai, Guoshuai

Cai, Guoxiang

Cai, Haoyang

Cai, He‐yuan

Cai, Hongfu

Cai, Hua‐Jie

cai, jianqiang

Cai, Jinlian

Cai, Jiong

Cai, Juan

Cai, Kaican

Cai, Meijuan

Cai, Miao‐Miao

Cai, Qidong

Cai, Ruijun

Cai, Sanjun

Cai, Wenjuan

Cai, Wenli

Cai, Xianyi

Cai, Xiao‐Yan

Cai, Xin

Cai, Xinyong

Cai, Xiujun

Cai, Xu‐Wei

Cai, Yixin

Cai, Zeman

Cai, Zhigang

Caignard, Anne

Caini, Saverio

Cakir, Bekir

Cakmak, Mehmet

Calaf, Gloria

Caldeira, Daniel

Calderoni, Matilde

Califano, Raffaele

Calin, George Adrian

Calip, Gregory

Calistri, Daniele

Callander, Natalie

Calvert, Melanie

Calvisi, Diego F.

Camacho‐Rivera, Marlene

Cambier, Samantha

Campana, Luca

Campbell, Anna

Campbell, Matthew T.

Campbell, Moray

Campbell, Peter

Campbell, Robert

Campidelli, Arnaud

Campo, Flaminia

Campo, Elias

Campomenosi, Paola

Campos Veras Giorgi, Lucimara

Campos, Nicole

Camps, Carlos

Camus, Vincent

Candelaria, Myrna

Canella, Claudia

Canepa, Matilde

Canettieri, Gianluca

Canfell, Karen

Cannavicci, Angelo

Cannegieter, Suzanne C.

Canter, Robert

Cantile, Monica

Cantor, Scott

Cantrell, Sarah

Cañueto, Javier

Canzona, Mollie

Cao, Chao

Cao, Cong

Cao, Dedong

Cao, Deliang

Cao, Hongyong

Cao, Hua

Cao, Jian

Cao, Jingyu

Cao, Jinlin

Cao, Jinlong

Cao, Junyu

Cao, Ka‐Jia

Cao, Ke

Cao, Liang

Cao, Liming

Cao, Ling

Cao, Liping

Cao, Qiang

Cao, Qizhi

Cao, Ruifen

Cao, Shousong

Cao, Shuangqing

Cao, Sumei

Cao, Su‐Mei

Cao, Tiefeng

Cao, Xuan

Cao, Xu‐Chen

Cao, Yihai

Cao, Yingying

Cao, Yiqun

Cao, Yu

Cao, Yuandong

Cao, Yuepeng

Cao, Zhi

Capelletti, Martina Maria

Capelozzi, Vera

Capitanio, Arrigo

Capitanio, Umberto

Capurso, Gabriele

Caputo, Emilia

Caramel, Julie

Carbone, Carmine

Carbone, David P.

Carceller, Fernando

Cardel, Majken

Cardona, Andrés

Cardona, Andres F.

Carducci, Michael

Carey, Lisa

Carles, Joan

Carleton, Neil

Carlin, Leslie

Carlino, Matteo S.

Carlson, Jay W.

Carnahan, Leslie

Carpenter, Erica

Carpenter, Kristen

Carpten, Jon

Carr, Peter

Carr, Robert

Carraro, Dirce

Carrasco Pro, Sebastian

Carrato, Alfredo

Carrera, Cristina

Carreras, Giulia

Carrillo, José

Carroll, William

Carter, Hannah

Carter, Patricia

Cartmel, Brenda

Cartron, Guillaume

Caruso, Rosangela

Carvalho, André F.

Carvalho, Andre F.

CasadeiGardini, Andrea

Casal, Roberto F.

Casali‐da‐Rocha, José Casal

Casanova, Emilio

Casanova, Michela

Casciano, Roman

Cascinu, Stefano

Casey, Graham

Casey, Ruth

Cash, Hannes

Cashin, Peter H.

Casillas, Jacqueline

Cassano, Alessandra

Cassel, Brian

Cassidy, Richard

Cassone, Marco

Castagnola, Elio

Castel, David

Castel, Helene

Castellano, D.

Castellano, Joan

Castellanos‐Rubio, Ainara

Castells, Antoni

Castells, Mariana

Castells, Xavier

Castellvi‐Bel, Sergi

Castelvi Bel, Sergi

Castera, Laurent

Castillo Almeida, Natalia

Castillo, C. Fernandez‐Del

Castillo, Jorge

Castillo, Jorge J.

Castle, Philip E.

Caston, Nicole

Castro Junior, Gilberto

Castro, Anna

Castro, Maria G.

Cathcart, Paul

Cathomas, Richard

Cattelan, Annamaria

Catto, James

Caudle, Abigail S.

Caulin, Carlos

Cava, Claudia

Cavaillé, Mathias

Cavenee, Webster K.

Caviglia, Gian Paolo

Cavisi, Diego

Cavo, Michele

Cazap, Eduardo

Cazzola, Mario

Ceballos, Kathy M.

Ceballos‐Picot, Irene

Cebon, Jonathan S.

Cebulla, Colleen

Ceci, Francesco

Cederholm, Tommy

Celebi, Julide

Celik, Haydar

Celio, Luigi

Cella, David

Cen, Caize

Ceppi, Francesco

Ceppi, Lorenzo

Cereda, Emanuele

Cerini, Matias

Černe, Katarina

Cervantes, Andrés

Cervello, Melchiorre

Ceschin, Rafael

Cespedes, Elizabeth

Cesselli, Daniela

Ceyhan, Güralp

Cha, Jae

Chadburn, Amy

Chaddad, Ahmad

Chaft, Jamie E.

Chagoya, Alejandro

Chai, Xiangfei

Chai, Jian

Chai, Ke‐Qun

Chai, Rejie

Chai, Young Jun

Chaiyachati, Krisda H.

Chaiyakunapruk, Nathorn

Chakiryan, Nicholas

Chakraborty, Santam

Chakraborty, Sohini

Chalandon, Yves

Chalder, Trudie

Chalfant, Charles

Chambers, Carole

Chambers, Suzanne

Chamie, Karim

Chan, Aden K.

Chan, Andrew T.

Chan, Annie Waifong

Chan, Anthony

Chan, Christina

Chan, Daniel Tak Mao

Chan, David

Chan, Godfrey

Chan, Henry

Chan, Jason Y. K.

Chan, Jimmy Yu‐Wai

Chan, Justin

Chan, Kelvin

Chan, Michael

Chan, Nancy

Chan, Oscar S. H.

Chan, Stephen L.

Chan, Steve

Chan, Timothy A.

Chan, TingFung

Chan, Wing Lok

Chandra, Asish

Chandra, Dinesh

Chandran, Dhanya

Chandrasekar, Thenappan

Chandy, Mammen

Chang, Andrew

Chang, Bernard P.

Chang, Chien‐Hsing

Chang, Ching‐Mao

Chang, Chiung‐Fang

Chang, Chiz‐Tzung

Chang, Chun‐Hung

Chang, Daniel

Chang, David W.

Chang, Elaine

Chang, George

Chang, Hui

Chang, Jeffrey

Chang, Jeremy

Chang, Jianhua

Chang, Jinjia

Chang, Joseph T.

Chang, Jung Min

Chang, Kai‐Ping

Chang, Liang

Chang, Lung‐Ji

Chang, Nijia

Chang, Pengyu

Chang, Shih‐Ching

Chang, Steve

Chang, Steven

Chang, Wai Hoong

Chang, Weilong

Chang, Wenjun

Chang, Yanxin

Chang, Yih‐Hsin

Chang, Yih‐Leong

Chang, Ying‐Jun

Chang, Yoon Jung

Chang, Yu‐Jia

Chang, Yunli

Chantada, Guillermo

Chantharakhit, Chaichana

Chanvorachote, Pithi

Chao, Ce

Chao, Hann‐Hsiang

Chao, Herta H.

Chao, Joseph

Chao, Jui‐I

Chao, K. S. Clifford

Chao, Samuel

Chao, Tsu‐Yi

Chao, Wang

Chao, Yin‐Kai

Chapin, Brian

Chapman, Jennifer

Chapman, Paul

Charalambous, Andreas

Charalampakis, Nikolaos

Charette, Nicolas

Chargari, Cyrus

Chari, Aswin

Charif, Omar El

Charlson, Fiona

Charoenkwan, Kittipat

Charoenlap, Chris

Chatelut, Etienne

Chatopadhyay, Subhayan

Chatterjee, Avishek

Chatterjee, Nabanita

Chatterjee, Sanjoy

Chattopadhyay, Abanti

Chaturvedi, Pankaj

Chatzistamatiou, K.

Chatzistamatiou, Kimon

Chau, Ian

Chaudhary, Kumardeep

Chauhan, Aastha

Chauhan, Aman

Chavan, Prachi

Chavez, Julio

Chavez‐MacGregor, Mariana

Chawla, Sant

Chayama, Kazuaki

Chayanupatkul, Maneerat

Che, Guo‐Wei

Che, Xiaofang

Cheang, Maggie

Checcucci, Enrico

Check, Devon

Checketts, Jake Xavier

Chemaly, Roy F.

Chen, Yongsong

Chen Wei

Chen, Aileen B.

Chen, Amy

Chen, Ann

Chen, Baoan

Chen, Baoqing

Chen, Benjamin J.

Chen, Ben‐Kuen

Chen, Bihong T.

Chen, Bin

Chen, Bo

Chen, Bobin

Chen, Ceshi

Chen, Chang

Chen, Chi‐An

Chen, Chia‐Yu

Chen, Chien‐Chih

Chen, Chi‐Long

Chen, Chi‐Wei

Chen, Chuang

Chen, Chun

Chen, Churong

Chen, Clark

Chen, Dan

Chen, Danqi

Chen, David

Chen, Di

Chen, Dong

Chen, Erfei

Chen, Fa

Chen, Fangying

Chen, Fei

Chen, Fen

Chen, Gang

Chen, Guihua

Chen, Haiquan

Chen, Hang

Chen, Haojun

Chen, Haoyan

Chen, Hongda

Chen, Hongliang

Chen, Honglin

Chen, Hsuanyu

Chen, Huanjie

Chen, Huanwen

Chen, Jarvis T.

Chen, Jen‐Shi

Chen, Jia

Chen, Jia‐Bo

Chen, Jian

Chen, Jianbin

Chen, Jian‐Guo

Chen, Jiani

Chen, Jiapeng

Chen, Jiayan

Chen, Jia‐Yan

Chen, Jie

Chen, Jinbin

Chen, Jing‐Song

Chen, Joy

Chen, Ju

Chen, Jui‐Chieh

Chen, Jun

Chen, Junchao

Chen, Junquan

Chen, Juntian

Chen, Junxia

Chen, Kai

Chen, Kaiju

Chen, Ke

Chen, Ke‐Neng

Chen, Kexin

Chen, Kuan‐Yu

Chen, Kun

Chen, Laing

Chen, Lan

Chen, Lei

Chen, Leilei

Chen, Liang

Chen, LiangAn

Chen, Lidan

Chen, Lin

Chen, Lizhang

Chen, Luyao

Chen, Mei‐Ling

Chen, Ming

Chen, Ming‐Yuan

Chen, Minshan

Chen, Mu

Chen, Mulin

Chen, Nanhui

Chen, Nian‐Ping

Chen, Paul

Chen, Pei Jer

Chen, Pei‐Song

Chen, Peng

Chen, Ping

Chen, Puxiang

Chen, Qi

Chen, Qiang

Chen, Qianjun

Chen, Qianming

Chen, Ronald

Chen, Ruei‐Ming

Chen, Rui

Chen, Ruoqing

Chen, Sam Li‐Sheng

Chen, Shaoan

Chen, Shaw‐Ji

Chen, Shih Fang

Chen, Shih‐Ann

Chen, Shih‐Hsiang

Chen, Shinn‐Cherng

Chen, Shi‐yao

Chen, Shuai

Chen, Shuang

Chen, Shuqing

Chen, Shuzhen

Chen, Siqi

Chen, S‐Y

Chen, Szu‐Ta

Chen, Szu‐Tah

Chen, T.

Chen, Tao

Chen, Tianfeng

Chen, Tianhui

Chen, Tien‐Hsing

Chen, Tingna

Chen, Tom Wei‐Wu

Chen, Tony Hsiu‐Hsi

Chen, Wan‐Nan

Chen, Wanqing

Chen, Wei

Chen, Weichang

Chen, Wen

Chen, Wen‐Fang

Chen, Wenyu

Chen, Xi

Chen, Xian

Chen, Xiang

Chen, Xiangjin

Chen, Xianju

Chen, Xiaojin

Chen, Xiaojun

Chen, Xiaonan

Chen, Xiaosong

Chen, Xiaoyuan

Chen, Xiaozhong

Chen, Xin

Chen, Xingdong

Chen, Xing‐xing

Chen, Xinhua

Chen, xinru

Chen, Xintong

Chen, Xinye

Chen, Xin‐Zu

Chen, Xiuping

Chen, Xu

Chen, Xuguang

Chen, Xun

Chen, Y.

Chen, Yajin

Chen, Yajing

Chen, Yanming

Chen, Yansu

Chen, Yi‐An

Chen, Yinan

Chen, Yingbo

Chen, Yingtai

Chen, Yingying

Chen, Yinting

Chen, Yin‐Ting

Chen, Yi‐Ru

Chen, Yi‐Wei

Chen, Yiyi

Chen, Yong

Chen, Yongbing

Chen, Yongming

Chen, Yongqiang

Chen, Yuanyuan

Chen, Yuan‐Yuan

Chen, Yuhan

Chen, Yuh‐Min

Chen, Yun‐Ching

Chen, Zhanghan

Chen, Zhao

Chen, Zhengtang

Chen, Zhenzhong

Chen, Zhe‐Sheng

Chen, Zhihong

Chen, Zhiqiang

Chen, Zhishui

Chen, Zhiyao

Chen, Zhuo

Chen, Zihang

Cheng, Xuedi

Cheng, Alfred

Cheng, Ann‐Lii

Cheng, Bin‐Feng

Cheng, Chia‐Hsiung

Cheng, Chieh‐Lung

Cheng, Dongfeng

Cheng, Feixiong

Cheng, Guanghui

Cheng, Huaidong

Cheng, Huanqing

Cheng, Jie

Cheng, Lei

Cheng, Ming

Cheng, Quan

Cheng, Robert Y. S.

Cheng, Shao‐Yi

Cheng, Shujun

Cheng, Shuqun

Cheng, Tingyuan

Cheng, Wei

Cheng, Wen‐Fang

Cheng, Xiaolong

Cheng, Xin

Cheng, Yanxiang

Cheng, Ying‐Sheng

Cheng, Yingsheng

Cheng, Yuan

Cheng, Yufeng

Cheng, Zhen

Chenxi, Pan

Cheong, Jae Youn

Cheong, Sok Ching

Chera, Bisham

Cherbal, Farid

Cherezov, Dmitry

Cherniack, Andrew

Chernov, Andrei V.

Cherradi, Nadia

Cherry, Mohamad

Cheruvu, Vinay

Chesi, Marta

Cheung, Annie N. Y.

Cheung, Chun Hei Antonio

Cheung, Ka Shing

Cheung, Man Kit

Cheung, Matthew

Cheung, Nai‐Kong

Cheung, Yiu‐Fai

Chevreau, Christine

Chevron, Brooke

Chew, Min Hoe

Chhabra, Yash

Chi, Jen‐Tsan Ashley

Chi, Kim

Chi, Pei‐Dong

Chi, Ping

Chi, Yihebali

Chiang, Alan K. S.

Chiang, Gloria C.

Chiang, Veronica

Chiao, Elizabeth Y.

Chiappa, Corrado

Chiappella, Annalisa

Chiaravalloti, Agostino

Chiba, Mitsuru

Chida, Keigo

Chie, Eui Kyu

Chien, Chih‐Yen

Chien, Chi‐Wen

Chien, Huei‐Tzu

Chien, Li‐Nien

Chihara, D.

Chikandiwa, Admire

Chikazawa, Kenro

Chilamkurthy, Sasank

Chilcot, Joseph

Childs, Daniel

Chim, Chor Sang

Chin, Ho Jun

Chinnaiyan, Arul M.

Chino, Junzo

Chinot, Olivier

Chinsky, Ravi

Chiocca, Susanna

Chiorazzi, Nicholas

Chiorean, Gabriela

Chiou, Shu‐Ti

Chiou, Tzeon‐Jye

Chirieac, Lucian R.

Chiron, David

Chitapanarux, Imjai

Chiu, Alexander

Chiu, Chao‐Hua

Chiu, Han‐Mo

Chiu, Keith

Chiu, Michelle

Chiu, Tai‐Jan

Chloe, De Witte

Chmielowski, Bartosz

Chmura, Steve

Ch'ng, Ewe Seng

Chng, Wee‐Joo

Cho, Eun‐Kyung

Cho, Eunyoung

Cho, Eun‐Young

Cho, Hyo Jung

Cho, Hyunsoon

Cho, Jae Hee

Cho, Jae Yong

Cho, John

Cho, Juhee

Cho, Kang Su

Cho, Kathleen R.

Cho, Oyeon

Cho, Sang

Cho, Sang Kyu

Cho, Seoae

Cho, Seok‐Goo

Cho, Shih‐Feng

Cho, Sun Wook

Cho, William

Cho, William C. S.

Cho, Woo‐Cheal

Cho, Yong Beom

Cho, Young Sin

Chobanian, Nishan

Choe, Lina

Choi, Bryan D.

Choi, Chang Heon

Choi, Cheol Woong

Choi, Dong Wook

Choi, Dong‐Wook

Choi, Doo Ho

Choi, Gi Hong

Choi, Gyu‐Seog

Choi, Jae‐Ki

Choi, Jin‐Hyuk

Choi, Kang‐Yell

Choi, Kyung Un

Choi, Seung Hee

Choi, Yoon Seong

Choi, Yoonla

Chong, Matthew Alan

Chong, Tie

Chong, Wei

Choti, Michael A.

Chou, Wen‐Chien

Chou, Che‐Yi

Chou, Chia‐Hung

Chou, James

Chou, Wen‐Chi

Chou, Yu‐Ching

Chou, Yu‐Ting

Chouaid, Christos

Choudhury, Aaheli

Choudry, Haroon

Choueiri, Toni

Chouhan, Jay

Chouvardas, Panagiotis

Chow, Daniel

Chow, Edward

Chow, Eric

Chow, James

Chow, Laura Q. M.

Chow, Ronald

Chow, Wong‐Ho

Chowdhury, Sanjib

Choyke, Peter

Chris, Rini

Christakoudi, Sofia

Christalle, Eva

Christensen, Diana Hedevang

Christensen, Jesper

Christensson, Anders

Christiani, David

Christiansen, Christian Fynbo

Christiansen, Jon

Christofori, Gerhard

Christopeit, Maximilian

Chrysikos, Dimosthenis

Chu, Chi‐Ming

Chu, Daehyun

Chu, Gilbert

Chu, Liang

Chu, Ling‐Hui

Chu, Minjie

Chu, Qian

Chu, Tang‐Yuan

Chu, Tianqing

Chu, yiwei

Chu, Zhangbo

Chua, Kevin

Chua, Melvin

Chuang, Chien‐Chia

Chuang, Heng‐Chang

Chuang, Shih‐Chang

Chuang, Shih‐Sung

Chugh, R.

Chugh, Rashmi

Chun, Jaeyoung

Chun, Jerold

Chun, Kyung‐Hee

Chun, Stephen G.

Chung, Christine

Chung, Dongjun

Chung, Jing‐Gung

Chung, Jinsoo

Chung, Joo‐Seop

Chung, Ka‐Fai

Chung, Moon Jae

Chung, Woo‐Baek

Church, David

Chutipongtanate, Somchai

Ciafrè, Silvia Anna

Cianchetti, Marco

Cibas, Edmund S.

Cifra, Barbara

Cihoric, Nikola

Cioce, Mario

Ciradiello, Fortunato

Ciribilli, Yari

Ciurea, Stephan

Clancy, Thomas

Clark, Amy

Clark, Clancy J.

Clark, Jeffrey

Clarke, Blaise A.

Clarke, Christina

Clarke, Christina A.

Clarke, Megan

Clarke, Ronald J.

Clary, Alecia

Classe, Jean‐Marc

Claudia, Lanari

Clavel, Jacqueline

Clayburgh, Daniel

Clay‐Williams, Robyn

Cleeland, Charles

Clement‐Duchene, Christelle

Clements, Meredith

Clifford, Gary

Clinton, Steven

Cloos, Jacqueline

Closson, Kalysha

Cobbs, Charles

Cockwill, Alison

Coffey, John Calvin

Coffey, Robert

Cofie, Leslie

Coghill, Anna E.

Cohen, Aaron

Cohen, Alexander T.

Cohen, Ian

Cohen, Joachim

Cohen, Lorenzo

Cohen, Michael

Cohen, Seth

Cohen, Tamara

Cohen‐Cutler, Sally

Cohn, Richard

Cohrs, Austin

Colaprico, Antonio

Colas, Eva

Colditz, Graham A.

Coldman, AJ

Coldman, Andrew J.

Cole, Donald C.

Cole, Peter D.

Coleman, Craig I.

Coleman, Jonathan

Coleman, Robert

Coles, Theresa

Coletta, P. Louise

Colibaseanu, Dorin

Collado, Rosa

Collatuzzo, Giulia

Collen, C.

Colleoni, Marco

Collinge, Elodie

Colombo, Massimo

Colombo, Nicoletta

Colomer, Dolors

Colonna, Marc

Cominetti, Marcia Regina

Comins, Charles

Conant, Emily F.

Concin, Nicole

Cong, Rong

Cong, Wenming

Cong, Xianling

Conklin, Heather M.

Connolly, Gregory

Connor, Avonne

Connor, Mark

Connors, Jean Marie

Conrad, E.

Constantinescu, Stefan

Constine, Louis S.

Conteduca, Vincenza

Conti, Fabio

Cook, Daniel

Cook, Gary

Cook, Linda

Cook, Michael B.

Cooke, Colin R.

Cooke, Ian

Cool, Derek

Cooney, Kathleen

Cooper, Gregory

Cooper, Lee A. D.

Cooper, Matthew

Cooper, Ziva D.

Copeland, Laurel A.

Copp, Tessa

Coppola, Francesca

Coquet, Jean

Corcoran, Niall M.

Corcoran, Ryan

Corley, Douglas

Cormier, Janice

Cornell, Robert Frank

Cornillon, Jérôme

Cornish, Alex

Coronnello, Marcella

Corradini, Paolo

Correa, Denise D.

Corrias, Maria V.

Cortellini, Alessio

Cortes, Jorge

Cortesi, Laura

Cortinovis, Diego

Cosentino, Chiara

Cossarizza, Andrea

Costa, Luciano

Costa, Manuel Joao

Costantini, Adrien

Costantini, Massimo

Costantini, Susan

Costanzo, Giovan Giuseppe Di

Costea, Daniela

Costello, Brian

Costello, Joseph

Costi, Renato

Cote, David

Cote, Michele

Cotogni, Paolo

Cottereau, Anne‐Ségolène

Cottone, Francesco

Couch, Fergus J.

Coughlin, Steven

Coughlin, Steven S.

Counago, Felipe

Coupé, Veerle

Courneya, Kerry

Coutinho, Iolanda

Coutre, Steven E.

Cowey, C. Lance

Cox, Edward

Cox, Kim

Cozzi, Luca

Cragun, Debi

Craig, Andrew W.

Cramb, Susanna

Cramer, Paula

Crane, Tracy

Cranmer, Lee

Crawford, Jeffrey

Crawford, Rebecca

Crawley, Esther

Creagh, Em

Creighton, Chad

Cremolini, Chiara

Crescenzi, Anna

Crespo‐Sanjuan, Jesus

Cress, Rosemary

Crevenna, Richard

Crew, Katherine

Crinò, Lucio

Cripe, Timothy Peter

Criscito, Maressa

Crispen, Paul L.

Crispin‐Ortuzar, Mireia

Cristofanilli, Massimo

Crivellaro, Cinzia

Crombet, Tania

Cron, Randy Q.

Cronin, Kathy

Crooks, Bruce

Crosbie, Emma

Cross, Darren A. E.

Cruz, Nataly

Cruz‐Diaz, David

Cryan, John

Cubiella, Joaquin

Cuccia, Francesco

Cuellar, Alison

Cuello, Mauricio A.

Cuesta‐Guardiola, Tatiana

Cuevas, Carlos De las

Cui, Fusheng

Cui, Guangying

Cui, Guangying

Cui, Hongli

Cui, Jian

Cui, Jinquan

Cui, Jiuwei

Cui, Kaisa

Cui, Lin

Cui, Lining

Cui, Manhua

Cui, Pengfei

Cui, Qinghua

Cui, Ri

Cui, Rongjun

Cui, Shuxia

Cui, Shu‐Zhong

Cui, Xin‐Wu

Cullin, Nyssa

Culos‐Reed, Nicole

Cuneo, Antonio

Cuomo, Alessandra

Curiel, Tyler

Curigliano, Giuseppe

Curioni‐Fontecedro, Alessandra

Curley, Steven A.

Curran, Emily

Cury, Fabio L.

Cushman, Mary

Custers, Jose

Cutrona, Sarah L.

Cutter, David

Cuzick, Jack

Cybulska‐Stopa, Bożena

Cybulski, Cezary

Czarnecka, Anna M.

Czarnocka, Barbara

D. V. Ganesha

D. Marrelli

D. Hagstrom, Amanda

D. Sears, Dorothy

Da Pozzo, Luigi Filippo

da Silva, Benedito

da Silva, Luciane

Da'ar, Omar

Dabakuyo, Sandrine

Dachew, Berihun Assefa

Dacic, Sanja

Daddi, Niccolo

D'Agostino, Norma

Dahan, Meryl

Dahglas, Iyas

Dahiya, Rajvir

Dahlmann, Mathias

Dahlstrom, Kristina

Dai, Bo

Dai, Danian

Dai, Dong Jun

Dai, Hongying

Dai, James

Dai, Jianrong

Dai, Meng

Dai, Mushui

Dai, Penggao

Dai, Sheng

Dai, Shuang

Dai, Siqi

Dai, Wei

Dai, Xiangpeng

Dai, Xiaofeng

Dai, Yu‐Jun

Dai, Zhi‐Jun

Dalianis, Tina

Dalle, Stéphane

Dall'olio, Filippo

Damen, Johanna

Damodar, Sharat

D'Amore, Francesco

Damude, Samantha

Dan, Gheorghe‐Andrei

Dan, Han C.

Danafar, Hossein

Dandan, Xiong

D'Andrea, Elvira

Daneshmand, Siamak

Daneshmandi, Saeed

Danesi, Romano

Dang, Anh

Dang, Jun

Dang, Rui

Dang, Yiwu

D'Angelica, Michael

Daniel, Rhian M.

Daniels, Johannes M. A.

Danielson, Kirsty M.

Danis, Jean

Danziger, Samuel

Danziger, Samuel A.

Daohua, Shi

Dar, Nazir

Darb‐Esfahani, S.

Darlix, Amélie

Darragh, Teresa

Dartigeas, Caroline

Darvin, Pramod

DAS, Bhudev

Das, Chhanda

Dasari, Arvind

Dasch, Burkhard

Dasgupta, Santanu

Daste, Ghislaine

Datta, Rohini

Datta, Sandipan

Datta, Soumitra S.

Daubisse‐Marliac, Laetitia

Daud, Adil

Daugaard, Gedske

Davar, Diwakar

Davaran, Soodabeh

Daver, Naval

David, Elizabeth

Davidoff, Amy

Davidson, Brittany

Davies, Clare

Davis, Courtney

Davis, Joanne N.

Davis, Keith

Davis, Lara

Davison, JM

Davuluri, Ramana V.

Dawes, Andrew

Dawsey, Sandy

Dawson, Laura

Dawson, Laura A.

Day, Terry A.

De Angelis, Carmine

de Biase, Dario

de Bono, Johann

de Carvalho, Paulo

De Cocker, Laurens J. L.

de Crea, Carmela

de Fabritiis, Paolo

De Felice, Francesca

de Franco, Paola

De Giorgi, Ugo

De Giorgi, Vincenzo

de Gramont, Aimery

de Groot, Hester

de Jong, Mathilde

de la Chapelle, Albert

De Laurentiis, Michelino

De Marinis, Filippo

De Marzo, Angelo

De Meue, Elisabeth

De Meulenaere, Astrid

de Moerloose, Barbara

De Moor, Janet

de Munck, Linda

de Nonneville, Alexandre

De Palma, Giuseppe

de Paula, André

De Ponti, Fabrizio

De Preter, Katleen

De Rossi, A.

De Ruiter, Michiel

de Smith, Adam

de Souza, Dyego L. B.

de Vathaire, Florent

De Vos, Willem

de Vries, I Jolanda M.

de Vries, Esther

de Vries, Jolanda

de Wilt, J. H. W.

de Wit, Ronald

De, Subhajyoti

Dean, Michael

Dean, Preston

DeAngelis, Carlo

DeAngelo, Daniel J.

Deben, Christophe

Debieuvre, D.

Debily, Marie‐Anne

Debourdeau, Philippe

Deckert, Markus

Deflon, Victor

Defour, Jean‐Philippe

Defraene, Gilles

Deganello, Alberto

Degboe, Arnold

Degenhardt, Louisa J.

Dekker, Joost

Del Chiaro, Marco

Del Paggio, Joseph

Del Rivero, Jaydira

Delanaye, Pierre

Delaney, Thomas

Delea, Thomas E.

Delen, Emre

Delft, Frederik

Delgado‐Bellido, Daniel

Delgoffe, Greg M.

Delmonte, Angelo

Delnevo, Cristine

Deluca, Holly

Deluche, Elise

DeMarsh, Samantha

DeMatteo, Ronald

DeMatteo, Ronald P.

DeMichele, Angela

Demirel, Max

Demogeot, Nicolas

Dempsey, Eugene

Dempster, Martin

Denbo, Jason

Denburg, Avram

Denda, Tadamichi

Deng, Haiteng

Deng, Chu‐Xia

Deng, Guangtong

Deng, Haijun

Deng, Hong

Deng, Jihong

Deng, Meihai

Deng, Min

Deng, Qiaoling

Deng, Sisi

Deng, Teng

Deng, Wei

Deng, Weiwei

Deng, Wuguo

Deng, Yang

Deng, Yuwei

Deng, Zhenfeng

Deng, Zhimin

Denis, Gerald

Denton, Brian

Denys, Alban

Deodato, Francesco

Deotare, Uday

Depalma, Michele

DePinho, Ronald

Der, Channing J.

Derchain, Sophie

DeRouen, Mindy

DeRouen, Mindy C.

Derré, Laurent

Desai, Niyati

Desai, Pinkal

Desbois‐Mouthon, Christele

Deshields, Teresa

Deshmukh, Ashsish

Deshpande, Ravindra

Dess, Robert

Dettenkofer, Markus

Detterbeck, Frank

Dettino, Aldo

Dettogni, Raquel

Deuker, Marina

Deutsch, Alexander

Dev, Rony

Devesa, Susan

Devi, Gayathri

Devilee, Peter

Deville, Curtiland

Devillier, Raynier

DeVoe, Jennifer E.

Dey, Mahua

Dey, Biswajit

Dey, Prasanta

Dhanda, Sandeep

Dhanya, Sethumadhavannair Rajalekshmi

Dhar, Deepanshi

Dharma, Sanam

Dharmarajan, Arun

Dhillon, Haryana

Dhir, Mashaal

Dhodapkar, Kavita M.

Dhupkar, Pooja

Di Bonito, Paola

Di Carlo, Veronica

Di Cataldo, Andrea

Di Cosimo, Serena

Di Lalla, Vanessa

Di Lorenzo, Giuseppe

Di Maio, Massimo

Di Nunno, Vincenzo

Di, Li Jun

Diakowska, Dorota

Dialani, Vandana

Diamantopoulos, Panagiotis

Diamond, Jennifer

Dias de Oliveira, Natasha Favoretto

Diaz, Luis

Diaz, Luis A.

Díaz‐Gavela, Ana A.

Dick, Julia

Dicker, Adam

Dickerson, Suzanne

Diederichs, Sven

Diehn, Maximilian

Dietrich, Dimo

Dietrich, Pierre‐Yves

Dijksterhuis, Willemieke

Dimitrov, Dimiter S.

Dimopoulos, MA

Ding, Bingqian

Ding, Boni

Ding, Guangyu

Ding, Hui‐Guo

Ding, Kefeng

Ding, Peirong

Ding, Qiang

Ding, Ruoxi

ding, shangwei

Ding, Wen‐Xing

Ding, Yi

Ding, Yongfeng

Ding, Zhongxiang

Dingemans, Anne‐Marie C.

Dingley, Andrew J.

Dinnes, Jacqueline

Dinney, Colin

Dinu, Monica

Dionne‐Odom, J Nicholas

Diop, Gora

DiPippo, Vincent A.

Ditzel, Henrik

Dixit, Manjusha

Dixon, Jesse

Djidjik, Reda

D'journo, Xavier Benoit

Djulbegovic, Benjamin

Długosz‐Danecka, Monika

Do, In‐Gu

Do, Mai

Do, Young Rok

Dodd, Natalie

Doebele, Robert

Doebele, Robert C.

Doege, Daniela

Doehner, Hartmut

Dogan, Haluk

Dogra, Samrita

Doherty, Jennifer

Doherty, Mark

Dohn, Line

Doi, Hisayo

Doki, Noriko

Dolan, Mary Eileen

Dolan, Elizabeth

Dolznig, Helmut

Dombret, Herve

Domchek, Susan

Domenech, Marta

Dominici, Massimo

Dona, Maria Gabriella

Donadon, Matteo

Donahue, Timothy

Dong, Baijun

Dong, Di

Dong, Dong

Dong, Hua

Dong, Jing

Dong, Jingcheng

Dong, Jun

Dong, Le Thanh

Dong, Liang

Dong, Lihua

Dong, Lin

Dong, Pei

Dong, Pin

Dong, Qiongzhu

Dong, Sheng

Dong, Xiaoqun

Dong, Ying

Dong, Yumei

Dong, Zhe

Dong, Zhiqiang

Dongwei, Xue

Donovan, Kristine A.

Dooley, James

Dooley, Steven

Doorbar, John

Dorff, Tanya

Doroudinia, Abtin

Dorritie, Kathleen

Dorval, Michel

Dosaka‐Akita, Hirotoshi

dosReis, Susan

Dossett, Lesly

Douketis, James D.

Dowlati, Afshin

Dowling, Emily C.

Downing, Amy

Downs‐Canner, Stephanie

Dowsett, Mitch

Doyle, Lisa

Doyle, Maria B. Majella

Dp, Wu

Dragoumis, Dimitrios M.

Dranoff, Glenn

Dreij, Kristian

Dressler, Lynn

Dreveny, Ingrid

Drexler, Hans

Dreyling, Martin

D'Rhummo, Keven A.

Drilon, Alexander

Drogemoller, Britt

Droguet, Mickael

Drokow, Emmanuel

Drolet, Melanie

Dror, Vardit

Drukker, Karen

D'Souza, Anita

D'Souza, David

Du, Cheng‐You

Du, Dexiao

Du, Guobo

Du, Haining

Du, Jiajun

Du, Jianyang

Du, Mulong

Du, Peng

Du, Shasha

Du, Shenlin

Du, Tao

Du, X.

Du, Xianglin

Du, Xiaohui

Du, Xin

Du, Xinhui

Du, Xinru

Du, Yanzhi

Du, Yingying

Du, Yong

Duan, Hu‐BIn

Duan, Shao‐Bin

Duan, Xin

Duan, Zhenfeng

Duane, F. K.

Duarte Valencia, Edgar

Dubashi, Biswajit

Dubois, Manon

Dubois, Thierry

Dubourg, Laurence

Duchnowska, Renata

Ducreux, Michel

Duda, Dan

Duduyemi, Babatunde Moses

Due, Hanne

Dueholm, M.

Duenas‐Gonzalez, Alfonso

Duerksen‐Hughes, Penelope

Duflo, Suzy

Dufresne, Armelle

Duijf, Pascal

Duijf, Pascal H. G.

Duijn, Cornelia M. Van

Dulmen, Sandra van

Dulong, Sandrine

Duma, Narjust

Dumaij, Ineke

Duman, Nuriye B.

Dumas, Agnes

Dumas, Pierre

Dumitrascu, Traian

Dummer, Reinhard

Dunbar, Erin

Dunlap, Jennifer

Dunleavy, Kieron

Dunlop, Malcolm

Dunn, Joel T.

Dunn, Lara

Dunn, S. Terence

Dunst, Juergen

Dupre, Aurélien

Dupuis, L. Lee

Durando, Paolo

Durani, Urshila

Durante, Cosimo

Duraturo, Francesca

Durot, C.

Durrani, Raisa J.

Durst, Christopher R.

Dusetzina, Stacie

Dustler, Magnus

Dutt, Amit

Dutta, A.

Dutta, Pranab

Dutta, Shubham

Dutta, Soumitra

Duval, Alex

Duvic, Madeleine

Dwek, Miriam V.

Dyer, Matthew

Dyregrov, Atle

Dyson, Jane

Dyson, Julian

Dziadziuszko, Rafal

Dzimitrowicz, Hannah

Dzobo, Kevin

E. K. Holl

Eapen, Mary

Earl, Helena M.

Earle, Craig C.

Eary, J. F.

Easaw, Jay

East, James E.

Eastman, Boryana

Eberhardt, W. E.

Eberly, Lauren

Ebot, Ericka

Ebrahimabadi, Maryam

Ebrahimi, Ardalan

Ecke, Thorsten

Ector, Geneviève

Edet, Bassey

Edin, Sofia

Ediz, Caner

Edwards, Ann‐Marie

Edwards, Rhiannon

Eeles, Rosalind A.

Eertwegh, Alfonsus J. M. van den

Effah, Clement

Efferth, Thomas

Efficace, Fabio

Efstathiou, Jason

Egan, Pamela

Egeblad, Mikala

Egevad, Lars

Egle, Alexander

Ehdaie, Behfar

Ehlers, Mark

Ehninger, Gerhard

Ehrhardt, Mathew

Eidenschink, Benjamin

Eigentler, Thomas

Eigl, Bernhard

Eisfeld, Ann‐Kathrin

Ejaz, Muslima

Eke, Iris

Ekenga, Christine

El Bairi, Khalid

El Haddad, Hanine

El Koueiry, Anthony

El Osta, Badi

El Saghir, Nagi

El Sayed, Mohamed

Eladl, Ahmed

Elattar, Ahmed

Eleftheriadis, Theodoros

El‐Galaly, Tarec Christoffer

Elgebaly, Ahmed Saber

Elias, Anthony

Elinav, Eran

Elisabeth Daldrup‐Link, Heike

Elisei, Rossella

Elizabeth, Krakow

El‐Jawahri, Areej

El‐Khazragy, Nashwa

Elkholi, Islam

Elkord, Eyad

Eller, Yannick

Ellinger, Jörg

ElliotStieglitz, Elliot Stieglitz

Ellis, Mark

Elmahdy, Ahmed

Elmore, Joann G.

Elmore, Shekinah

El‐Omar, Emad M.

Eloy, Jean Anderson

Elsawy, Wael

Elsayad, Khaled

Elsayed, Inas

El‐Sayes, Nader

El‐Serag, Hashem B.

El‐Shal, Amal S.

ElShamy, Wael M.

ElSohly, Mahmoud A.

Elvin, Julia A.

Elwood, Mark

Elzahry, Mai

Emam, Sherif

Emancipator, Kenneth

Emi, Manabu

Emile, Jean‐Francois

Emmenegger, Urban

Emmerick, Isabel Cristina

Emmett, Louise

Endo, Itaru

Endo, Shinji

Enewold, Lindsey

Eng, Cathy

Eng, Kevin

Engel, Christoph

Engelhardt, Ellen G.

Engelhardt, Monika

Engelman, Jeffrey A.

English, Diana

English, Ian

Ennis, Sarah

Enomoto, Nobuyuki

Enright, Katherine

Enshaeifar, Shirin

Eom, Hyeon Seok

Epperla, Narendranath

Epskamp, Sacha

Erber, Wendy

Eri, Rajaraman

Erichsen, Rune

Ericsson, Christer

Erim, Daniel

Eroglu, Zeynep

Erovic, Boban M.

Ersbøll, Annette

Erskine, Holly E.

Eschrich, Steven

Escobar, Gabriel J.

Escribano Sotos, Francisco

Escudier, Bernard

Eskandari, Nahid

Eşkazan, Ahmet Emre

Eskens, Ferry A. L. M.

Eslami, Ahmad Ali

Eslick, G. D.

Esmaeili, Abolghasem

Esmaeili, Habibollah

Espinas, Josep

Essani, Karim

Esteller, Manel

Esteva, Magdalena

Estey, Elihu H.

Estrov, Zeev

Etienne, Gabriel

Eun, Jung Woo

Eun, Jungwoo

Evangelista, Laura

Evangelou, E.

Evans, James J.

Evans, William

Eveillard, Marion

Evenhuis, Richard

Evens, Andrew

Everts, Regula

Extermann, Martine

Ezeife, Doreen

Ezekwudo, Daniel

Ezendam, Nicole P. M.

Ezenwankwo, Elochukwu

Ezziddin, Samer

Fabbrini, P.

Fabian, Flora

Fabiani, Emiliano

Fabiani, Roberto

Fabricio, Aline S. C.

Fabris, Luca

Facchini, G.

Facchini, Gaetano

Facchini, Segio

Facon, Thierry

Fadaei, Sara

Fadul, Camilo

Faghih, Zahra

Fagotti, Anna AF

Faguet, Guy

Faiena, Izak

Fais, Stefano

Faje, Alexander

Fakhreddine, Mohamad

Fakhry, Carole

Fakih, Marwan

Falanga, Anna

Falcone, Alfredo

Falconer, Henrik

Falconi, Massimo

Falkenberg, N.

Fallah, Aria

Fallah, Mahdi

Fallara, Giuseppe

Fallon, Marie

Falo, Catalina

Famularo, Simone

Fan, Caibin

Fan, Chunhong

Fan, Dandan

Fan, Daping

Fan, Guoxin

Fan, Hong

Fan, Jia

Fan, Jinhai

Fan, Lihua

Fan, Rengen

Fan, Ruitai

Fan, RuiTai

Fan, Weifei

Fan, X.‐G.

Fan, Xiaoming

Fan, Yaqi

Fan, Yonggang

Fan, Yun

Fan, Zhimin

Fancellu, Alessandro

Fandrey, Joachim

Fang, Andrew

Fang, Baijun

Fang, Feng

Fang, John

Fang, Luxiong

Fang, Mengjie

Fang, Mingming

Fang, Songhua

Fang, Taiyong

Fang, Weiyi

Fang, Wen‐Liang

Fang, Xiaolan

Fang, Xinyi

Fang, Xuefeng

Fang, Ya

Fang, Yan

Fang, Ye‐ying

Fang, Yi

Fang, Yue

Fanqiao, Meng

Faquin, William

Faquin, William C.

Farace, Paolo

Fararouei, Mohammad

Farassati, Faris

Fares, Aline

Farias, Albert

Farioli, Andrea

Farkas, Dora

Färkkilä, Anniina

Farland, Daniel C. Mc

Farrell, C.

Farrugia, Mark

Faryabi, Robert

Farzadfar, Farshad

Fasching, Peter

Fassan, Matteo

Fassina, Ambrogio

Fathzadeh, Mohsen

Fattore, Liana

Faulkner, Sam

Faux, Steven

Fawzy, Manal

Fawzy, Manal S.

Fay, André

Fayanju, Oluwadamilola

Fayette, Jerome

Fazio, Nicola

Fazio, Vito Michele VMF

Fearon, Kenneth C.H

Fecci, Peter

Fedele, Monica

Fedewa, Stacey

Fehniger, Todd

Fehrenbacher, L.

Fehrmann, Rudolf S. N.

Fei, K.

Fei, Ma

Fei, Xiang

Fei, Xuejie

Feilotter, Harriet

Feld, Jordan

Feld, Ronald

Felderhof, Joeke M.

Feldman, Steve

Feldman, Darren R.

Feldman, Lisa

Felip, Enriqueta

Felix, Arthur

Felix, Ashley

Fellman, Bryan

Felsher, Dean W.

Feng, Bin

Feng, Chenchen

Feng, Gen‐Sheng

Feng, Ji

Feng, Jihong

Feng, Jinggao

Feng, Jun

Feng, Lei

Feng, Lin

Feng, Liyuan

Feng, Qianjin

Feng, Qianxi

Feng, Shuangwu

Feng, Tsui‐Hsia

Feng, Weiwei

Feng, William

Feng, Xiaodong

Feng, Xingyu

Feng, Xueping

Fenk, Roland

Fennell, Lochlan

Ferguson, Mark K.

Ferlay, J.

Fernandez, Rafael

Fernández‐Avilés, Francesc

Fernández‐Montes, A.

Fernandez‐Pol, Sebastian

Fernandez‐Tilapa, Gloria

Ferrando, Adolfo

Ferrara, Felicetto

Ferrari, Alize

Ferrari, Pietro

Ferrarotto, Renata

Ferreira Castro, Ana

Ferreira, Arlindo

Ferreira, Lis

Ferreri, Andres J. M.

Ferreri, Andrés José Maria

Ferretti, Elisabetta

Ferris, Robert L.

Ferro, Matteo

Ferrolino, Jose

Ferrone, Cristina

Ferroni, Patrizia

Ferrucci, Luigi

Fertig, Elana

Fessele, Kristen

Fetcko, Kaleigh

Feugier, Pierre

Feuilly, Marion

Fiano, Ryan

Ficarra, Vincenzo

Fiedler, Katja

Fiedorowicz, Anna

Fielding, Adele

Fifis, Theodora

Figueiredo, Eberval Gadelha

Figueiredo, Francisco Winter dos

Figueiredo, N.

Filetti, Marco

Filipp, Fabian

Filippatos, Gerasimos

Filippatos, Theodosios D.

Finazzi, Tobias

Finch, Emily

Fink, James R.

Finucane, Anne M.

Fiocco, Marta

Fiore, Marco

Fiore, Michael C.

Fioresi, Rita

Fiori, Cristian

Firth, Amy L.

Fisch, Margit

Fischer, Janina

Fischer, Grant

Fischer‐Valuck, Benjamin

Fischman, Aaron M.

Fish, Laura

Fisher, Carla

Fisher, Richard I.

Fisher, Robert A.

Fitch, Margaret I.

Fitzcharles, Mary‐Ann

Fitzgerald, Timothy

Fitzmaurice, Christina

Flaherty, Keith

Flahou, Bram

Flaig, Thomas

Flanagan, Adrienne

Flanagan, James M.

Flanders, Adam E.

Flechtner, Hans‐Henning

Fleming, Fergal

Fleming, Jason

Fletcher, Christopher D. M.

Fletcher, Joel

Fletcher, Jonathan

Fletcher, Jonathan A.

Flinn, Ian W.

Florea, Natalia

Flores, Efren J.

Flores, Raja

Floyd, Scott

Flukes, Stephanie

Foerster, Milena

Fogelman, David

Fokas, E.

Foldi, Julia

Folgueira, Maria Aparecida

Folkerts, Megan

Folkesson, J.

Folprecht, Gunnar

Folsom, Aaron

Fond, Guillaume

Fonkem, Ekokobe

Fonseca, Adriana

Fontes‐Sousa, Mário

Fonti, Rosa

Foo, Dominic Chi‐Chung

Forbes, Stuart J.

Force, Jeremy

Forconi, Francesco

Forero, Roberto

Foresta, Carlo

Forgeard, Nathalie

Forjaz, Gonçalo

Forouhi, Nita G.

Forschner, Andrea

Forshew, Tim

Forssell‐Aronsson, Eva

Forster, Tobias

Fossati, Nicola

Foster, Claire

Foster, Kim

Foster, Paul

Fouladi, Nasrin

Fountzilas, Elena

Fowler, Nathan

Fox, Chris

Fox, Elizabeth

Fox, Louis

Fradley, Michael

Frampton, Adam E.

Frampton, Garrett

Francis, Nick A.

Francis, Steve

Franco, Edian F.

Franco, Eduardo

Franco, Pierfrancesco

Frandon, Julien

Frankel, Wendy

Fransson, Emma

Frantellizzi, Viviana

Frayling, Ian M.

Frederick, Natasha

Freedland, Stephen

Freedman, Rachel A.

Freeman, Gordon J.

Freeman, Vincent

Freire, Guilherme

Freitas, Leandro

Fresneau, Brice

Frey, Melissa

Freyer, David

Frezza, Anna Maria

Friboulet, Luc

Frick, Kevin

Fricklas, Betsy

Fridrichova, Ivana

Friebel‐Klingner, Tara

Fried, Eiko

Frieden‐Korovkina, Victoria

Friedenreich, Christine

Friedewald, Sarah

Friedl, Thomas W.P

Friedman, Claire

Frisk, Gabriella

Fritz, Jason

Froelich, Matthias

Fröhlich, Holger

Fromm, Anja

Fronteira, Ines

Fruehauf, John

Frühwald, Michael

Frumovitz, Michael

Fu, Bin

Fu, Denggang

Fu, Deyuan

Fu, Hung‐Chun

Fu, Jianfei

Fu, Jie

Fu, Jun‐Hui

Fu, Kai

Fu, Kai

Fu, Lei

Fu, Lin

fu, liyun

Fu, Pingfu

Fu, Shunjun

Fu, Siqing

Fu, Tao

Fu, WeiHua

Fu, Wenbin

Fu, Yujie

Fu, Zhenfu

Fu, Zhenming

Fu, Zhongxue

Fuca, Giovanni

Fuchs, Charles

Fuerst, Alois

Fugazzola, Laura

Fuhui, Hiroshi

Fuji, Makoto

Fujii, Masashi

Fujii, Takeo

Fujii, Yasuhisa

Fujimoto, Daichi

Fujimoto, Naohiro

Fujimura, Taku

Fujino, Shiki

Fujishima, Fumiyoshi

Fujita, Kohei

Fujita, Shiro

Fujita, Yuka

Fujitani, Kazumasa

Fujiwara, Yoshinori

Fujiwara, Yutaka

Fukada, Junichi

Fukagawa, Takeo

Fukuda, Minoru

Fukuhara, Noriko

Fukui, Takayuki

Fukuoka, Shota

Fukuyama, Tamaki

Fulciniti, Franco

Fulda, Simone

Fuller, Peter

Fung, John

Funke, Roel

Furbetta, Niccolo

Furlong, Fiona

Furness, Kate

Furubayashi, Nobuki

Furukawa, Yoichi

Furuta, Saori

Fuse, Kyoko

Fuss, Ashley Ann

Futreal, Andrew

Gabani, Prashant

Gabrail, Nashat

Gabrilovich, Dmitry

Gabrovska, Plamena

Gacci, M.

Gadalla, Ramy

Gadgeel, Shirish

Gadiya, Mayur

Gaertner, Jan

Gagliardi, Anna R.

Gainor, Justin

Gaist, D.

Gaitanidis, Apostolos

Gajewski, Thomas F.

Galanopoulos, Athanasios

Galati, Luisa

Galetta, Domenico

Galigniana, Mario

Galizia, Gennaro

Gallagher, Rollin M.

Galldiks, Norbert

Gallego‐Ortega, David

Galli, Giorgio G.

Gallie, brenda

Gallimore, Awen

Gallo, Angela

Gallo, Marco

Galmiche, Antoine

Galon, Jerome

Galvao, Daniel

Galvez, Julio

Galvin, Angéline

Gambacorti‐Passerini, Carlo

Gamblin, T Clark

Gan, Boyi

Gan, Gregory N.

Gan, Shu

Gandaglia, Giorgio

Gandara, David

Gandemer, Virginie

Gandhi, Jatin

Gandhi, Ajeet

Gandhi, Leena

Gandhi, Nishant

Gandhi, Tejal

Gandhirajan, Rajesh Kumar

Ganesan, Prasanth

Gangi, Alexandra

Ganly, Ian

Gannot, Israel

Gansler, Ted S.

Ganti, Apar Kishor

Ganz, Patricia

Gao, Bo

Gao, Chao

Gao, Chenlin

Gao, Chunfang

Gao, Dongmei

Gao, Guang‐Xun

Gao, Guangyu

Gao, Hong‐Ye

Gao, Hui

Gao, JianJiong

Gao, Jianjun

Gao, Jia‐Rong

Gao, Li

Gao, Min

Gao, Ming

Gao, Peng

Gao, Qinglei

Gao, Shenglin

Gao, Shuichao

Gao, Wei

Gao, Xiaoning

Gao, Xin

Gao, Yanzheng

Gao, Yi

Gao, Yi‐Bo

Gao, Yingbin

Gao, Yingchun

Gao, Yong

Gao, Yongshun

Gao, Yuanhong

Gao, Zhenzehn

Gapstur, Susan M.

Garassino, Marina Chiara

Garayoa, Mercedes

Garbar, Christian

Garbuglia, Annarosa

Garcia Abos, Miriam

Garcia, Kevin

Garcia, Matthew

Garcia, Montse

Garcia‐Carbonero, Rocio

Garcia‐Escudero, Ramon

Garcia‐Manero, Guillermo

Garden, Adam

Garde‐Noguera, Javier

Garg, Madhur

Garg, Tullika

Gargano, Julia Warner

Gargano, Stacey

Garland, Sheila

Garmire, Lana X.

Garmo, Hans

Garnsworthy, Philip C.

Garon, Edward

Garrido, Melissa

Garrido‐laguna, Ignacio

Garzon, Ramiro

Gasiorowska, Emilia

Gaspard, Nicholas

Gaßler, Nikolaus

Gatalica, Zoran

Gately, Kathy

Gater, Adam

Gatta, Gemma

Gatta, Roberto

Gauchotte, Guillaume

Gaudet, Mia

Gaulard, Philippe

Gaunez, Nicolas

Gauthier, Martin

Gavin, Anna

Gavriilaki, Eleni

Gay, Francesca

Gay, Hiram

Gazzaniga, Paola

Ge, Chunlin

Ge, Hua

Ge, Jie

Ge, Minghua

Ge, Penglei

Ge, Xiaoyan

Gebauer, Bernhard

Geboes, Karen

Gebremariam, Alem

Gebski, Val

Geerts, Dirk

Gehrig, Paola

Geiger, Jessica

Gelagay, Abebaw Addis

Geller, David

Geller, Melissa

Gempt, Jens

Genden, Eric M.

Geng, Xiuwen

Gennaro, Nicolo

Gennaro, Nicolò

Gentille Sanchez, Cesar

Georgakopoulos‐Soares, Ilias

george, biju

George, Marina

George, Prema

George, Rani

George, Saby

George, Suzanne

Georgiades, Christos

Georgian, Badicu

Georgiesh, Tatiana

Gepner, Yftach

Geraghty, Dominic

Gerber, Bernd

Gerger, Armin

Gergis, Usama

Gerhäuser, Clarissa

Germain, Claire

Germain, Guillaume

Gerratana, Lorenzo

Gertsch, Jurg

Getaneh, Abraham

Getz, Gad

Geyer, Charles

Ghaderi, Abbas

Ghadjar, Pirus

Ghaedi, Kamran

Ghai, Chirag

Ghandi, Leena

Gharaghani, Sajjad

Gharahkhani, Puya Gharahkhani

Ghassemifar, Reza

Ghebrehewet, Sam

Gheibi hayat, Seyed Mohammad

Ghia, Amol J.

Ghia, Paolo

Ghidini, Antonio

Ghidini, Michele

Ghisoni, Eleonora

Gholizadeh Navashenaq, Jamshid

Ghoochani, Ali

Ghorbani, Saied

Ghosh, Jaya

Ghosh, Sankar

Ghossein, Ronald

Giaccone, Giuseppe

Giaj Levra, Niccolò

Giannella, Luca

Gianni, Cazzaniga Gianni

Gianni, Luca

Giannini, Giuseppe

Gianotti, Luca

Gibbs, Jennifer

Gibney, Geoffrey

Gibson, Michael

Gibson, S. B.

Gibson, Todd

Giebel, Sebastian

Giera, Martin

Giger, Maryellen

Gigot, Jean‐Francois

Gil Ferreira, Carlos

Gil, Ziv

Gilani, Syed

Gil‐Bazo, Ignazio

Gilbert, Alexandra

Gilbert, Jill

Giles, Graham

Gilham, David

Gilkey, Melissa

Gilks, Blake

Gill, David

Gill, Harinder

Gill, Pavandeep

Gill, Sharlene

Gillespie, Erin

Gillies, Derek J.

Gillio‐Tos, Anna

Gillison, Maura

Gillison, Maura L.

Gilmore, Nikesha

Gil‐Moreno, Antonio

Gilron, Ian

Gines, Pere

Gioia, Gerard

Giordano, Sara

Giovanella, Luca

Giovanna Elena Giraldi, Annamaria

Giovannetti, Elisa

Giraldi, Laura

Giret, Teresa

Giri, Veda

Girotra, Monica

Giuliani, Massimo

Giuliano, Armando

Giulietti, Matteo

Giurisato, Emanuele

Giuseppe, Viale

Given, Barbara

Gjermundson, Hali J.

Gkika, E.

Gkouziouta, Angeliki

Glaesmer, Heide

Glare, Paul

Glaser, Scott

Gleave, Martin

Glenn, Beth A.

Glimelius, Ingrid

Glynne‐Jones, Rob

Gnani, Daniela

Gobe, Glenda C.

Gobin, Romila

Gobitti, Carlo

Goda, Jayant

Godier, Anne

Goecks, Jeremy

Goedendorp, M. M.

Goekbuget, Nicola

Goel, Arun

Goel, Rakesh

Goepfert, Ryan

Goeppert, Benjamin

Goette, Martin

Goff, Barbara

Goffin, John

Goggins, Michael

Gogineni, E.

Goh, Vicky

Gökyer, Ali

Golatta, Michael

Gold, Jason

Gold, Jason S.

Goldberg, Jason

Goldberg, Richard M.

Goldgar, David E.

Goldhaber, Samuel Z.

Goldhaber‐Fiebert, Jeremy

Goldinger, Simone

Goldman, Jonathan

Goldsbury, David

Goldsby, Robert

Goldschmidt, Hartmut

Goldstein, Benjamin

Goldstein, Daniel

Goldstein, David

Goldszmidt, Mark

Goldvaser, Hadar

Goldwasser, François

Golfieri, Rita

Golshan, Mehra

Golubovskaya, Vita

Gomez, Daniel

Gomez‐Martin, Carlos

Goncalves, Anthony

Goncalves, Vania

Gondo, Nobuhisa

Gonen, Mithat

Gong, Aihua

Gong, Chang

Gong, Jianping

Gong, Jian‐Ping

Gong, Jie

Gong, Lan

Gong, Longlong

Gong, Ping

Gong, Xiao‐Chang

Gong, Yan

Gong, Yanbin

Gong, Yanqing

Gong, Youling

Gong, Yuehua

Gong, Yuewen

Gong, Yuqi

Gong, Zhaohui

Gong, Zhicheng

Gongzhao, Qin

Gonzalez, Raul S.

Gonzalez, S.

Gonzalez‐Perez, Abel

González‐San Segundo, Carmen

Goo, Jin Mo

Goodfellow, Paul

Goodman, Aaron

Goodman, Aaron M.

Goodman, Annekathryn

Goodman, Karyn

Goodman, Marc

Goodman, Melody S.

Goodwin, Pamela J.

Goodwin, Rachel

Goossens, Steven

Gorczynski, Reginald

Gordon, Howard

Gordon, Louisa

Gore, John

Gore, Martin

Gorenek, Bulent

Gores, Greg

Gorey, Kevin

Gorin, Norbert

Gorlick, Richard

Gorlov, Ivan

Gosain, Rohit

Goss, Glenwood D.

Goswami, Sandeep

Goswami, Srikanta

Gota, Vikram

Gotesman, Moran

Goto, Akiteru

Goto, Hiroaki

Gou, Shanmiao

Gou, Xin

Gough, Karla C.

Goujon, Stéphanie

Gourley, Charlie

Govina, Ourania

Govindan, Ramaswamy

Gowrishankar, Swarnalata

Goy, B.

Goyal, Gaurab

Gozali, Grace

Graaf, Pim de

Graber, Judith M.

Graboyes, Evan M.

Gracheva, Marina

Graetz, Ilana

Graff, Julie N.

Graham, Camila

Graham, Chris

Graham, Rondell

Gralla, Richard J.

Gramantieri, Laura

Grande, Enrique

Grani, Giorgio

Grant, Christa

Grant, William

Granzier, R.W.Y

Grasselli, Giacomo

Gratz, Iris

Grau, Maria

Graubard, Barry

Gravekamp, Claudia

Graves, Lee

Gravholt, Claus

Gray, R. T.

Gray, Stacy

Graziani, Grazia

Graziano, Francesco

Greathouse, K. Leigh

Greco, Angela

Green, Adele C.

Green, Beverly

Green, John

Green, Tamar

Greenberg, Alexandra

Greenberg, Caprice

Greenhalgh, Janette

Greenland, Philip

Greenlee, Heather

Greenup, Rachel

Greer, Joseph

Gregucci, F.

Greil, Richard

Greimel, Elfriede

Grelet, Simon

Gressin, Remy

Gri, Giorgia

Gridelli, Cesare

Griffin, Georgia

Griffin, James

Griffith, Brent

Griffith, Obi

Griffiths, Ewen

Grigorieva, Elvira

Grimshaw, Jeremy M.

Grindedal, Eli Marie

Grivas, Nikolaos

Grivas, Petros

Grobmyer, Stephen R.

Grogan, Raymon

Gromiha, M. Michael

Gronberg, Bjorn Henning

Gronchi, Alessandro

Gronowicz, Gloria

Groothuizen, Johanna

Gross, Cary

Grosse, Alexandra

Grosse, Claudia

Grosset, Christophe

Gross‐Goupil, Marine

Grossi, Francesco

Grossman, Stuart A.

Grossmann, Kenneth

Grosso, Giuseppe

Grotmol, Tom

Grover, Surbhi

Grozescu, Traian

Grubb, Anders

Gruber, Stephen B.

Grummet, Jeremy P.

Grunfeld, Eva

Gryfe, Robert

Gu, Dongying

Gu, Jun

Gu, Junjie

Gu, Ke

Gu, Lijuan

Gu, Shengqing

Gu, Tongjun

Gu, Wei

Gu, Xiaobin

Gu, Xiao‐Bin

Gu, Yang

Gu, Yuanyuan

Gu, Zhen

Guadagnolo, B. Ashleigh

Guan, Baozhang

Guan, Chao

Guan, Haixia

Guan, Jian

Guan, Jinqiu

Guan, Lianyue

Guan, Ming

Guan, Quanlin

Guan, Weiqun

Guan, Wenxian

Guan, X.

Guan, Xiaoxiang

Guan, Xin‐Yuan

Guan, Yongjun

Guancial, Elizabeth

Guang, Yang

Guarneri, Alessia

Guarneri, Valentina

Gubens, Matt

Guckenberger, M.

Guda, Chittibabu

Gudayu, Temesgen Worku

Gudiol, Carlota

Gudnason, Vilmundur

Guerra, Federico

Guglielmetti, Simone

Guh, Jih‐Hwa

Gui, Benedetta

Guidi, Gabriele

Guilbert, Jill

Guilcher, Greg

Guillem, Jose G.

Guimerà, Núria

Gujar, Shashi

Gujral, Sumeet

Gulati, Roman

Gulati, Shuchi

Guldin, Mai‐Britt

Guller, Ulrich

Gullett, Joseph

Gultekin, Murat

Gultie, Teklemariam

Gunasekera, Sanjeeva

Gunn, Christine

Gunn, G.

Gunter, Marc

Guo, Chengang

Guo, Chuanyong

Guo, Erna

Guo, Feng

Guo, Hao

Guo, Jhe‐Cyuan

Guo, Jianchun

Guo, Jun

Guo, Junming

Guo, Lan

Guo, Lanwei

Guo, Lijie

Guo, Ling

Guo, Linwei

Guo, Luopei

Guo, Mingzhou

Guo, Rong‐Ping

Guo, Shenglan

Guo, Shicheng

Guo, Shuilong

Guo, Tao

Guo, Wei

Guo, Weihua

Guo, Wendi

Guo, Xiang

Guo, Xiangqian

Guo, Xiao‐Mao

Guo, Ya

Guo, Yi

Guo, Ying

Guo, Yitian

Guo, You

Guo, Yuming

Guo, Zhu

Guo, Zong‐Sheng

Guoqiang, Xu

Gupta, Abha

Gupta, Abha A.

Gupta, Amit

Gupta, Avigeet

Gupta, Michael

Gupta, Ruta

Gupta, Sudeep

Gupta, Sumit

Gupta, Vijayalaxmi

Guren, Marianne

Gurney, James

Guru, Khurshid A.

Gutierrez, Antonio

Gutiontov, Stanley

Gutkind, J. Silvio

Guyatt, Gordon

Guzmán, Esther

Gyawali, Bishal

Ha, Eun Ju

Ha, Sang Yun

Haaland, Benjamin

Haas, Jennifer

Haas, Oscar

Haas, Rick

Habbous, Steven

Häberle, Lothar

Habib, Nagy

Habl, Gregor

Habra, Mouhammad

Habr‐Gama, Angelita

Hack, N.

Hacker, U. T.

Hackert, T.

Haddad, Peiman

Haddad, Riad

Hadkhale, Kishor

Hadziyannis, Emilia

Haese, Alexander

Haeusler, Gabrielle

Haferlach, Torsten

Hafler, David A.

Hagberg, Carin

Hagendijk, Marije

Haghi‐Aminjan, Hamed

Haghjooy Javanmard, Shaghayegh

Hagihara, Atsushi

Hagiwara, Shotaro

Hagiwara, Sumitaka

Haglund, Caj

Hagneman, Andrea

Hagoel, Lea

Hahlweg, Pola

Hahn, A.

Hahn, Andrew W.

Hahn, Erin

Hahnen, Eric

Haider, Stefan

Hait, Nitai

Haitao, Luo

Hajeer, Ali H.

Hakguder, Zeynep

Hakimi, A. Ari

Halaban, Ruth

Halabi, Susan

Halazun, Karim

Halbritter, Florian

Halfdanarson, Thorvardur

Hall, Richard

Hall, William

Halle, Mari

Haller, Heidemarie

Halloran, Stephen

Halperin, Daniel

Halperin, Jonathan L.

Ham, Won Sik

Hamadeh, Tala

Hamaguchi, Tetsuya

Hamai, Yoichi

Hameed, Meera

Hamel, Lauren

Hamid, Anis

Hamid, Omid

Hamilton, Ann

Hamilton, Betty K.

Hammarsten, Jan

Hammel, Pascal

Hammemi, Khaled

Hammer, Anne

Hammond, Ester M.

Hampel, Heather

Hamzeloo, Reza

Han, Dong‐Seok

Han, Bin

Han, Chang

Han, Chaofeng

Han, Chengbo

Han, Cheng‐Bo

Han, Chun

Han, Cong‐hui

Han, Fei

Han, Guang

Han, Guohong

Han, Jinkun

Han, Kwang Hyub

Han, Kwang‐Hyub

Han, Lei

Han, Leng

Han, Lu

Han, Minghui

Han, Paul

Han, Rui

Han, Sangyoung

Han, Suping

Han, Wei

Han, Wonshik

Han, Xiaoyan

Han, Xinwei

Han, Xuesong

Han, Yatian

Han, Yiqun

Han, Youngyih

Han, Yue

Han, Zhiyong

Hang, Dong

Hankey, Graeme J.

Hankins, Margaret

Hann, Hie‐Won

Hanna, G. G.

Hanna, George B.

Hanna, Glenn

Hanna, Nader

Hannafon, Bethany N.

Hannum, Susan

Hans, Didier

Hansel, Donna

Hansen, Aaron

Hansen, Bo Terning

Hansen, John‐Bjarne

Hansen, Ryan N.

Hanzlik, Emily

Hao, Chunyi

Hao, Dapeng

Hao, Jialing

Hao, Liguo

Hao, Min

Hao, Wende

Haque, Ariful

Haque, Md Abedul

Haque, Waqar

Hara, Masayasu

Harada, Hiroaki

Harada, Kaito

Harada, Toshiyuki

Haraldsdottir, Sigurdis

Harden, Kathleen

Hardie, Grahame

Harding, James

Hardy, Janet

Hardy, Kristina

Hargrave, Darren

Hargraves, Ian G.

Hari, Parameswaran

Harila‐Saari, Arja

Harju, Erika

Harky, Amer

Harland, Mark

Harms, Kelly

Harper, Jay

Harris, Adrian L.

Harris, Curtis C.

Harris, Holly

Harris, Jeremy

Harris, Ryan

Harrison, Claire

Harrison, Douglas

Harshman, Lauren

Hart, Nicholas

Hart, Robert

Härter, Martin

Hartlapp, Ingo

Hartman, Johan

Hartmann, Gunther

Hartmann, Steffi

Hartmann, Sylvia

Haruki, Koichiro

Haruna, Yoshimichi

Hasanov, Merve

Hasegawa, Sumitaka

Hasegawa, Yasuhisa

Hashemi, Mohammad

Hashibe, Mia

Hashiguchi, Yasunori

Hashiguchi, Yojiro

Hashii, Yoshiko

Hashim, Onn Haji

Hassan, Bass

Hassan, Imatiyaz

Hassan, Samah

Hassan, Waseem

Hassel, Jessica

Hata, Akito

Hata, Masaharu

Hata, Nobuhiro

Hatakeyama, Shingo

Hatefi, Arash

Hatt, Mathieu

Hatzis, Christos

Haubold, Johannes

Haug, Alexander

Haugen, Dagny

Hauke, Jan

Haun, Markus

Hauschild, Axel

Hauswald, Henrik

Haut, Elliott

Hawinkels, Lukas J.A.C

Hawk, Ernest

Hawkes, Eliza

Hawkins, Nikki A.

Hawkins, Robert

Hawley, S.T

Hay, Joel W.

Hay, Nissim

Hay, Roderick J.

Hayashi, Hideyuki

Hayashi, Kazuhiko

Hayashi, Tomoyuki

Hayashi, Toshinobu

Hayes, Alan

Hayes, Daniel

Hayes‐Lattin, Brandon

Haylett, Wendy J.

Hays, Ron

Hazama, Shoichi

Hazen, Stanley

Hazewinkel, Yark

He, Yi

He, Baochang

He, Baoxia

He, Bin

He, Boxue

He, Chaobin

He, Fucheng

He, Guicheng

He, Hongwei

He, Hongying

He, Hongyong

He, Huadong

He, Huarong

He, J.

He, Jian‐Zhong

He, Jie

He, Jin

He, Jing

He, Jingsong

He, Jinwei

He, Kang

He, Laichang

He, Min

He, Qifei

He, Qingnan

He, Rongquan

He, Rong‐Quan

He, Rui

He, Sirong

He, Songqing

He, Taiyu

He, Xia

He, Xianghui

He, XiaoZhou

He, Xiayun

He, Xijing

He, Xin

He, Xingkang

He, Xujun

He, Yijing

He, Yiping

He, Yizi

He, Yong

He, Yongfeng

He, Yuanmin

He, Yun

He, Yutong

He, Zhaohui

He, Zhenqiang

He, Zhen‐Yu

Healy, Claire

Heasley, Lynn E.

Heath, Jacqueline

Heavey, Susan

Hecht, Andreas

Heck, Matthias M.

Heerema, Nyla

Heffner, Jaimee L.

Hegarty, Sarah

Hegde, John

Hegi, Monika E.

Hegyi, Péter

Heideman, DaniÃ«lle A.M

Heidrich, Benjamin

Heijnsdijk, Eveline

Heilbroner, Samuel

Heim, Markus

Hein, Alexander

Heinävaara, Sirpa

Heindryckx, Femke

Heinemann, Udo

Heinemann, Volker

Heinrich, Daniel

Heinzerling, Lucie

Heldin, Carl‐Henrik

Helland, Åslaug

Helleday, Thomas

Hellmann, Matthew D.

Helmond, Noud van

Hembree, Timothy

Hemming, Alan W.

Hemmings, B.

Hemminki, Otto

Hempel, Dirk

Henderson, Louise M.

Henderson, Tara

Hendifar, Andrew

Hendriks, Lizza

Hendriks, Lizza E. L.

Hendrikse, Jeroen

Hendryx, Michael

Heng, Daniel Y. C.

Heng, Yu

Hennessy, Bryan

Henriksson, Roger

Henriquez, Mario

Henry, N. Lynn

Hens, Kristien

Henshall, Catherine

Hensley, Martee

Henson, Katherine E.

Heo, Dae Seog

Hepner, Adriana

Herbst, Roy

Herman, James G.

Hermelink, Kerstin

Hernaez, Ruben

Hernández, Greco

Hernandez‐Boussard, Tina

Herrera, Alex F.

Herrera‐Goepfert, Roberto

Herrmann, Anne

Herrmann, Sandra M.

Hersh, David S.

Hershman, Dawn

Hertz, Dan

Herzer, Kerstin

Herzig, Shoshanna

Hess, Georg

Hess, Moritz

Hessmann, Elisabeth

Hestetun, Kjersti Elvestad

Hetzel, Sara

Heuser, Christian

Heuser, Michael

Hewitt, Stephen

Heyland, Daren

Heymach, John

Hiasa, Yoichi

Hickey, Ryan

Hicks, Rodney

Hida, Tomoyuki

Hida, Toyoaki

Hieken, Tina J.

Higashi, Takahiro

Higgins, Kristin

Hihara, Jun

Hijioka, Susumu

Hildesheim, Allan

Hill, Andrew G.

Hill, Brian

Hill, Deirdre

Hill, Kevin P.

Hillemanns, Peter

Hillen, Marij

Hills, Robert K.

Hilpert, Felix

Hindi, Nadia

Hinds, Pamela

Hinoi, Takao

Hinton, Benjamin

Hinz, Andreas

Hirakawa, Tsuneaki

Hiramatsu, Kosuke

Hiraoka, Atsushi

Hirashima, Tomonori

Hirayama, Alexandre

Hirayama, Kazuhiro

Hirayama, Ryuichi

Hird, Amanda

Hirose, Yuichi

Hiroshima, Kenzo

Hirshoren, Nir

Hisakane, kakeru

Hisayama, Masayo

Hiscock, R. J.

Hitchcock, Kathryn E.

Hitchcock, Ying

Hitz, Felicitas

Hiwase, Devendra

Hjelmstedt, Sofia

Ho, Alan

Ho, Cheryl

Ho, P. Shing

Ho, Sheng‐Yow

Ho, Thai

Ho, Yuh‐Shan

Hobbs, Gabriela

Hoch, Stephan

Hochhauser, Daniel

Hockenberry, Marilyn

Hodak, Emmilia

Hodge, Allison

Hodi, Steve

Hodul, Pamela

Hoefnagel, Sanne

Hoelscher, Arnulf H.

Hofer, Silvia

Hoffman, Richard

Hoffman, Robert

Hoffmann, Tammy

Hoffmeister, Michael

Hofman, Véronique

Hofmann, Bradley

Hofmann, Wilma

Hofste, Lisa

Hofstetter, Wayne

Hofvind, Solveig

Hogendorf, Piotr

Hohenberger, P.

Hohenegger, Martin

Hohloch, Karin

Høilund‐Carlsen, Poul

Hojjat‐Farsangi, Mohammad

Holden, William

Holdhoff, Matthias

Holick, Michael F.

Holingue, Calliope

Hollebecque, Antoine

Hollenhorst, Peter C.

Holm, Matilda

Holmberg, Leona A.

Holmen, Sheri

Holmes, Frankie

Holmes, Jarrod P.

Holsinger, Chris

Homayounfar, Kia

Honavar, Santosh G.

Honda, Keigo

Hondermarck, Hubert

Hong, Bumsik

Hong, Julian

Hong, Peng

Hong, Seung‐Mo

Hong, Shaodong

Hong, Sung Joon

Hong, Sung Kyu

Hong, Tingting

Hong, Yang

Hong, Ying

Hong, Yong‐kil

Hong, Young‐Rock

Honma, Yoshitaka

Honzawa, Katsu

Hood, Leroy

Hoogendijk, Raoull

Hoogland, Aasha

Hoonakker, Peter

Hooper, Lee

Hoover, Robert N.

Hopkins, Ashley

Hoppe, Bradford S.

Hopper, John L.

Horibe, Keizo

Horiguchi, Shigeru

Horita, Yosuke

Horn, L.

Horn, Leora

Horng, Jiun‐Lin

Horowitz, Amir

Horowitz, David P.

Horowitz, Netanel

Horstmann, Martin A.

Hortobagyi, Gabriel

Hoshida, Yujin

Hosing, Chitra

Hoskin, Peter

Hoskins, Kent

Hosoda, Kei

Hosomi, Yukio

Hoster, Eva

Hotta, Katsuyuki

Hou, Baohua

Hou, Jinlin

Hou, Lei

Hou, Lijun

Hou, Ming‐Chih

Hou, Ru

Hou, Yingyong

Houghton, Peter J.

Houlston, Richard

Hourigan, Christopher S.

Houssami, Nehmat

Houvast, Ruben D.

Hov, Johannes

Hoven, Emma

Hoverman, J.

Howard, A. Fuchsia

Howell, Debra

Howell, Doris

Howell, Elizabeth

Howlader, Nadia

Hoy, Sheridan M.

Hoyer, Juliane

Hoyo, Catherine

Hozaka, Yuto

Hrstka, Roman

Hruban, Ralph H.

Hruby, George

Hsia, Renee Y.

Hsiao, Kuei‐Yang

Hsiao, Michael

Hsieh, Jason Chia‐Hsun

Hsieh, Chia‐Hsun

Hsieh, James J.

Hsieh, Ming‐Han

Hsieh, Po‐Fan

Hsieh, Yi‐Hsien

Hsu, Chia‐Lang

Hsu, Chih‐Hung

Hsu, Han‐Shui

Hsu, Hsung‐Ling

Hsu, Hui‐Ping

Hsu, John T. ‐A

Hsu, Jun‐Te

Hsu, Keng‐Fu

Hsu, Li

Hsu, Li‐Sung

Hsu, P. ‐K

Hsu, Po‐Kuei

Hsu, Ren‐Jun

Hsu, Yi‐Chiang

Hsu, Yi‐Chiung

Hsueh, Hsueh

Hu, Bingren

Hu, Chaosu

Hu, Chen

Hu, Cheng

Hu, Chuan

Hu, Dongsheng

Hu, Frank

Hu, Frank B.

Hu, Fujun

Hu, Fulan

Hu, Fuyan

Hu, Gang

Hu, Han Jie

Hu, Hao

Hu, Hongjie

Hu, Jianda

Hu, Jian‐Kun

Hu, Jichang

Hu, Jieping

Hu, Jiying

Hu, Junwu

Hu, Ke

Hu, Kenneth

Hu, Minggen

Hu, Mingqiu

Hu, Nan‐Bin

Hu, Qinyong

Hu, Raymond T. C.

Hu, Shaoshan

Hu, T.

Hu, Wangxiong

Hu, Wei

Hu, Xianglin

Hu, Xin

Hu, Xing‐Sheng

Hu, Xueqing

Hu, Ya‐Ling

Hu, Yan

Hu, Yanfeng

Hu, Yi

Hu, Yongcheng

Hu, Yu

Hu, Yunxiang

Hu, Yu‐Wen

Hu, Zhenbo

Hu, Zhenhong

Hu, Zheyu

Hu, Zhibin

Hu, Zhiyang

Hua, Baojin

Hua, Chung‐Ching

Hua, Keqin

Hua, Lei

Hua, Qingquan

Hua, Yi‐Jun

Hua, Yunpeng

Hua, Zheng

Huang, Ao

Huang, Chang‐Ming

Huang, Chao‐Yuang

Huang, Cheng

Huang, Chia‐Yen

Huang, Chien‐Ning

Huang, Chuan

Huang, Chuiguo

Huang, Da

Huang, Dan

Huang, Dingzhi

Huang, Emina

Huang, Eng‐Yen

Huang, Fei

Huang, Feng‐Ting

Huang, Gang

Huang, Guanqun

Huang, Guichuan

Huang, Hai

Huang, Hai‐Neng

Huang, Haiwen

Huang, He

Huang, Hecheng

Huang, Hsien‐Da

Huang, Huai‐Hsuan

Huang, Ji

Huang, Jian

Huang, Jiayi

Huang, Jing

Huang, Jiwei

Huang, Jun

Huang, Junting

Huang, Junxing

Huang, Kai

Huang, Kun

Huang, Kuo‐Hung

Huang, KW

Huang, Lu

Huang, Peng

Huang, Pintong

Huang, Qi

Huang, Qian

Huang, Qiang

Huang, Qichao

Huang, Qingyuan

Huang, Raymond

Huang, Ruoqing

Huang, Shaoli

Huang, Sheng‐Fang

Huang, Shenglin

Huang, Shiang‐Fu

Huang, Shuang

Huang, Shu‐Pin

Huang, Tao

Huang, Tianji

Huang, Tonghai

Huang, Tsui‐Chin

Huang, Tze‐Sing

Huang, Wenqi

Huang, Xin yu

Huang, XinWei

Huang, Xiong

Huang, Xuefeng

Huang, Ya‐jie

Huang, Yan

Huang, Yangqing

Huang, Yanqi

Huang, Yen‐Ming

Huang, Yi‐Hsiang

Huang, Yiqiang

Huang, Yongsheng

Huang, Yongye

Huang, Youguang

Huang, Yuelong

Huang, Yuhan

Huang, Zening

Huang, Zeqing

Huang, Ze‐Xin

Huang, Zhengjie

Huang, Zilu

Huang, Zongqiang

Huangfu, Chaoji

Hubbard, Joleen

Hubbard, Rebecca

Huber, Magdalena

Hubner, Richard A.

Huchko, Megan

Huchko, Megan J.

Hudson, Melissa

Hudson, Melissa M.

Huerta, JosÃ MarÃ‐a

Huff, Wei X.

Hufgard, Jillian

Hughes, Amy

Hughes, Cathy

Hughes, Danny R.

Hughes, Timothy P.

Hugosson, Jonas

Huguet, Florence

Huguet, Françoise

Hui, David

Hui, Kam

Hui, R.

Hui, Zhouguang

Huilgol, Yash

Huillard, Olivier

Hummel, Karin

Humphries, Gerald

Hunault, Mathilde

Hundsdoerfer, Patrick

Hung, Chaohung

Hung, Chia yen

Hung, Rayjean

Hung, Rayjean J.

Hunger, Stephen

Hunter, Jonathan

Huntsman, David

Huo, Dezheng

Huo, Jiege

Huo, Junyu

Huo, Longfei

Huo, Xiaokui

Huo, Y. R.

Huppert, Laura

Hur, Chin

Hurst, Jillian

Hursting, Stephen

Hurwitz, Herbert

Husain, Zain A.

Hussain, Syed

Hussaini, Mohammad

Hussan, Maher Al

Hussein, Tawbi

Hussein, Rasha S.

Husson, Olga

Hutson, Tom

Hutt, Suzanna

Hutterer, Georg

Hüttmann, Andreas

Huyghe, Eric

Hwang, Clara

Hwang, Dae Wook

Hwang, Eu Chang

Hwang, Gyu‐Sam

Hwang, In Gyu

Hwang, Jong Ha

Hwang, Ki‐Tae

Hwang, Sang‐Gu

Hwang, Shin

Hwang, Thomas I‐Sheng

Hyung, Sujin

Hyung, Woo Jin

Iacoboni, Gloria

Iacovelli, Roberto

Iafrate, A.

Iafrate, A. John

Ianotto, Jean‐Christophe

Iavarone, Massimo

Ibdah, Jamal

Ibrahim, Joe

Ibrahim, Nageatte

Ibrahim, Nuhad

Ibrahim, Wanis

Ichikawa, Daisuke

Ichikawa, Tomohiko

Ichikawa, Wataru

Ichikawa, Yasushi

Ide, Hiroki

Ideker, Trey

Idigoras, I.

iennaco, joanne

Ifrah, Norbert

Igawa, Satoshi

Ignatenko, Natalia

IIjima, Katunori

Iinuma, Hisae

Ikawa, Hiroaki

Ikeda, Jun‐ichiro

Ikeda, Masafumi

Ikeda, Mio

Ikejima, Takashi

Ikoma, Naruhiko

Ilic, Dragan

Ilic, Milena

Ilie, Marius

Ilson, David

Im, Annie

Im, Dana

Im, Jong Pil

Im, Seock‐Ah

Imad, Shureiqi

Imai, Reiko

Imai, Yoichi

Imaizumi, Kazuyoshi

Imamura, Fumio

Imanishi, Yorihisa

Imaya, Masayuki

Imberty, Anne

Imoto, Seiya

Imperiale, Thomas F.

Ina, Kenji

Inaba, Hiroto

Inamoto, Teruo

Inamura, Kentaro

Inari, Hitoshi

Incorvaia, Lorena

Indrak, Karel

Ingels, Alexandre

Ingram, Nicola

Innocenti, Federico

Inokuchi, Junichi

Inokuchi, Koiti

Inokuchi, Shoichi

Inomata, Minehiko

Inoue, Akira

Inoue, Manami

Inoue, Tadahisa

Intermesoli, Tamara

Inturrisi, Charles

Inwards, David

Ioka, Tatsuya

Iqbal, Javeed

Iriyama, Noriyoshi

Irrnki, Krishna

Irwin, Melinda

Irwin, Meredith S.

Isaac, Krista

Isaacs, Claudine

Isaacs, Lyle

Isaacs, William B.

Isaacson, Richard

Isacke, Clare

Iseki, Hiroshi

Isenberg‐Grzeda, Elie

Ishida, Hirotaka

Ishihara, Soichiro

Ishii, Genichiro

Ishii, T.

Ishii, Taisuke

Ishikawa, Shumpei

Ishikawa, Toshiaki

Ishimi, Yukio

Ishimoto, Takatsugu

Ishioka, Chikashi

Ishioka, Junichiro

Ishitsuka, Yosuke

Ishiyama, Hiromichi

Ishizu, Tamiko

Isidean, Sandra D.

Islam, Jessica

Iso, Hiroyasu

Issa, Ghayas

Issaka, Rachel

Issels, Rolf

Ito, Jun

Ito, Kagenori

Ito, Katsuhiro

Ito, Kazuto

Ito, Ken‐ichi

Ito, Tetsuro

Ito, Toshinori

Ito, Yasuhiro

Iurlo, Alessandra

Iv, Michael

Ivanescu, Cristina

Ivy, Morgan

Iwamoto, Takayuki

Iwata, Hiromitsu

Iwata, Shintaro

Iwata, Takashi

Iyama, Shinji

Iyengar, Neil M.

Iyer, Prajish

Izano, Monika

Izumi, Kouji

Izumiya, Masashi

J. Zeh III, Herbert

J.W.L. Aerts, Hugo

Jabado, Nada

Jabagi, Marie Joelle

Jabbour, Elias

Jachetti, Elena

Jackson, Vicki A.

Jacob, Aasems

Jacob, Francis

Jacobs, Colin

Jacobs, Eric J.

Jacobs, Linda A.

Jacobs, Michael A.

Jacobsen, Steven J.

Jacobson, Kenneth A.

Jacoby, Elad

Jadidi‐Niaragh, Farhad

Jaffee, Elizabeth M.

Jager, Martine J.

Jaglowski, Samantha

jagrewala, Javed N.

Jagsi, Reshma

Jain, Abhinav

Jain, Akshay

Jain, Michael

Jain, Nitin B.

Jain, Preetesh

Jain, Rajan

Jain, Rishi

Jain, Saket

Jain, Salvia

Jain, Shanu

Jalal, Ahmed Hasnain

Jalali, Rakesh

Jalilvand, Somayeh

Jamadar, Abeda

James, Alexander

James, Benjamin

James, Francis

James, Nicole

Jameson, Michael G.

Jamy, Omer

Janakiram, Murali

janakiram, murali

Janda, Monika

Jandial, Rahul

Jandorf, Lina

Jang, Jin‐Young

Jang, Won Sik

Jansen, Jesse

Jansson, Fredrik

Jantunen, Esa

Janvilisri, Tavan

Jao, Kevin

Jardin, Fabrice

Jareid, Mie

Jarnagin, William R.

Jarosek, Stephanie

Jaspers, J. P. M.

Javadinia, Seyed Alireza

Javeri, Arash

Javle, Milind

Jayasekera, Jinani

Jee, Sun Ha

Jeltsch, Albert

Jemal, Ahmedin

Jeng, Yung‐Ming

Jenkins, Chris

Jenkins, Mark A.

Jenkins, Nancy A.

Jensen, Jorgen Bjerggaard

Jensen, Lars Henrik

Jensen, Lasse

Jensen, Roxanne

Jeon, Hyun Woo

Jeon, Jihyoun

Jeon, Min Ji

Jeon, Sang‐Min

Jeon, Seong Soo

Jeon, Yoon Kyung

Jeon, Young‐Woo

Jeong, Yong Yeon

Jeong, Chang Wook

Jeong, Keun‐Yeong

Jeong, Oh

Jeong, Seung‐Yong

Jeong, Su‐Min

Jeong, Woo Kyoung

Jerkeman, Mats

Jernström, Helena

Jeronimo, Carmen

Jeronimo, Jose

Jeschke, Udo

Jesus Alvarez‐Cubero, Maria

Jethwa, Krishan

Jeung, Hei‐Cheul

Jeys, Lee M.

Jhanwar, Shalu

Jhaveri, Jaymin

Jhawar, Sachin

Jhingran, Anuja

Ji, Chengjian

Ji, Jiafu

Ji, Jianguang

Ji, Jiansong

Ji, Juling

Ji, Kangting

Ji, Qiankun

Ji, Qing‐hai

Ji, Zhigang

Jia, Haifei

Jia, Huijun

Jia, Manman

Jia, Ruipeng

Jia, Weihua

Jia, Xiuzhi

Jia, Yanfei

Jia, Yunlu

Jia, ZhongZheng

Jian, Jimo

Jiang, B.

Jiang, Biaobin

Jiang, Changchuan

Jiang, Chunteng

Jiang, Cong

Jiang, De‐Ke

Jiang, Dewei

Jiang, Guosong

Jiang, Hongchuan

Jiang, Huiqing

Jiang, Jie

Jiang, Jingmei

Jiang, Jingting

Jiang, Jinqiong

Jiang, Jun

Jiang, Lijuan

Jiang, Maorong

Jiang, Mengjie

Jiang, Nan

Jiang, Peng

Jiang, Qingping

Jiang, Ruoyu

Jiang, Senwei

Jiang, Shuheng

Jiang, Sunfang

Jiang, Taijiao

Jiang, Tao

Jiang, Wen‐qi

Jiang, Xian

Jiang, Xiaohan

Jiang, Xingjun

Jiang, Xingming

Jiang, Yafen

Jiang, Yangming

Jiang, Yiqiu

Jiang, Yixing

Jiang, Yizhou

Jiang, YiZhou

Jiang, Yong

Jiang, Yu

Jiang, Yuanjun

Jiang, Yuming

Jiang, Yuxin

Jiang, Yuyang

Jiang, Zhengye

Jiao, Peng

Jiao, Shunchang

Jiao, Yan

Jiao, Zheng

Jiawei, Luo

Jie Tay, Matthew Rong

Jie, Wen

Jifeng, Wang

Jimenez, Julio

Jimenez, Rachel

Jin, Baiye

Jin, Chenggen

Jin, Cheng‐Hao

Jin, Feng

Jin, Fengyan

Jin, Guangfu

Jin, Hao

Jin, Haosheng

Jin, Jie

Jin, Kairui

Jin, Kideok

Jin, Mingjuan

Jin, Mingming

Jin, Xin

Jin, Yang

Jin, Zhe

Jin, Zheng

Jing, Changwen

Jing, Hongyu

Jing, H‐Y

Jing, Rui‐jun

Jingjing, Ran

Jinsong, Wu

Jitkaew, Siriporn

Jiwei, Li

Jo, Ara

Jo, Young Suk

Jobczyk, Mateusz

Jobin, Christian

Jochems, Sylvia

Jochen, Schmitt

Joensuu, Heikki

Joffe, Steven

Johanning, Gary

Johansen, Christoffer

Johansson, Bertil

Johansson, Junko

Johansson, Mattias

Johansson, Peter

John, Joseph

Johnson, Claire

Johnson, Amy J. Wagoner

Johnson, David

Johnson, Douglas B.

Johnson, Jennifer

Johnson, Jonas

Johnson, Lisa

Johnson, Miriam J.

Johnson, Rebecca

Johnson, Rhys

Johnson, Ronald

Johnston Taylor, Elizabeth

Johnston, Emily

Johnston, James

Johnstone, Peter

Johnstone, Ricky

Jokubkiene, L.

Jolly, Mohit

Jonasch, Eric

Jones, Robin

Jones, Barbara L.

Jones, Dr Robin

Jones, G.

Jones, Jeffrey

Jones, Lee W.

Jones, Louise

Jones, Patricia

Jones, Steven

Jong, Marcus C. de

Jonkers, Jos

Jonsson, Göran

Joo, Inkyung

Jordanova, E.

Jorm, Anthony F.

Jose, Wesley

Josey, Michele

Joshi, Bharat

Joshi, Monika

Joshi, Shreyas

Joshua, Anthony M.

Joslin, Charlotte E.

Joura, Elmar

Ju, Dianwen

Ju, Huai‐Qiang

Ju, Jianfeng

Ju, Shaoqing

Juan, Hsueh‐Fen

Juang, Horng‐Heng

Judson, Benjamin

Juhász, Csaba

Juillerat, Pascal

Julius, Peter

Jun, Chi Li

Jun, Dae Won

Jun, He

Jun, Jae Kwan

June, Carl H.

Jung, Andreas

Jung, Chan Kwon

Jung, Hwoon‐Yong

Jung, Keum

Jung, Klaus

Jung, Seung Il

Juraleviciute, Marina

Jurczak, Wojciech

Just, Johannes

Jutten, Barry

K, Govind

Kaakoush, Nadeem

Kaaks, Rudolf

Kaatz, Scott

Kabarriti, Rafi

Kabir, Syed Rashel

Kaczirek, Klaus

Kadara, Human

Kadia, Tapan

Kadigamuwa, Chamila Chathuranga

Kadlubar, Susan

Kadokura, Hiroshi

Kadowaki, Shigenori

Kaehler, Katharina C.

Kaemmerer, Till

Kagawa, Shunsuke

Kahl, Brad

Kahles, Andre

Kahn, Michael

Kaida, Hayato

Kaira, Kyoichi

Kaito, Akio

Kaji, Kiichiro

Kajino, Taisuke

Kajoka, Happiness

Kakeji, Yoshihiro

Kaku, Shaka

Kakudo, Kennichi

Kalantari, Faraz

Kalantar‐Zadeh, Kamyar

Kalb, Luther

Kale, Hrishikesh

Kale, Vaishnaw

Kaliki, Swathi

Kallergi, Galatea

Kalliala, Ilkka

Kalluri, Raghu

Kalnicki, Shalom

Kalousová, Marta

Kalsekar, Anupama

Kalyan, Aparna

Kamachi, Hirofumi

Kamada, Tomoari

Kamat, Ashish

Kamba, Tomomi

Kambadakone, A.

Kamble, Pravin

Kamei, Takashi

Kamel, Mohamed Gomaa

Kamel‐Reid, Suzanne

Kaminska, Bozena

Kammerer‐Jacquet, Solène‐Florence

Kamrava, Mitchell

Kamson, David

Kanaan, Ziad

Kanai, M.

Kanai, Osamu

Kanai, Yae

Kanavos, Andreas

Kanda, Mitsuro

Kanda, Tatsuo

Kandala, Sri Kamal

Kandil, Emad

Kane, Ari

Kane, Christopher

Kaneda, Atsuhi

Kaneda, Atsushi

Kaneki, Kensuke

Kaneko, Shuichi

Kanemitsu, Yukihide

Kang, Chang Hyun

Kang, Chang‐Yuil

Kang, Dae Hwan

Kang, Daehee

Kang, Gyeong Hoon

Kang, Huafeng

Kang, Hyunseok

Kang, Il Jun

Kang, Jin‐Hyoung

Kang, John

Kang, Jun

Kang, Kai

Kang, Keon Wook

Kang, Min Kyu

Kang, Minhee

Kang, Sung Gu

Kang, Sung Wan

Kang, Tiebang

Kang, Wonseok

Kang, Xindan

Kang, Yoon‐Koo

Kango, Ghazal

Kanjanapan, Yada

Kann, Benjamin

Kanno, Atsushi

Kansy, Benjamin A.

Kantarjian, Hagop

Kantidakis, Georgios

Kantidakis, Theo

Kantoff, Philip W.

Kantor, Elizabeth D.

Kanwal, Raghav

Kao, Chia‐Hung

Kao, Jia‐Horng

Kao, Li‐Ting

Kao, Yee‐Hsin

Kaouk, Jihad H.

Kapadia, Nirav

Kaphingst, Kimberly

Kapiteijn, Ellen

Kaplan, Henry G.

Kapoor, Anil

Karachunskiy, Alexander

Karam, Amer

Karamık, Kaan

Karamouzis, Michalis

Karande, Anjali

Karatas, Omer

Karayan‐Tapon, Lucie

Karczmar, Greg

Karia, Pritesh

Karia, Pritesh S.

Karim, Mohammad

Karim, Sajjad

Karim‐Kos, Henrike E.

Kariyama, Kazuya

Kärkkäinen, Emmi

Karlitz, Jordan

Karlsson, Per

Karnon, Jonathan

Karpf, Adam

Karrman, Kristina

Karslioglu, Aydan

Karslioglu French, Esra

Karsono, Ramadhan

Karthigaiselvi, Murugesan

Karthik, Bommanan

Kasahara, Mureo

Kaseb, Ahmed

Kasenda, Benjamin

Kashif, Muhammad

Kashiwabara, Kosuke

Kashyap, Mehr

Kasi, Anup

Kasper, Bernd

Kasper, Stefan

Kasprzak, Aldona

Kassam, Alisha

Kastner, Christof

Kastrinos, Fay

Kasubova, Ivana

Kasurinen, Aaro

Katada, Chikatoshi

Katakami, Nobuyuki

Katano, Harutaka

Kataoka, Kazunori

Kataoka, Naoyuki

Kataoka, Tatsuki

Katapodi, Maria

Katayama, Ryohei

Kater, Arnon

Kather, J.

Kather, Jakob Nikolas

Kato, Katsuhiko

Kato, Koji

Kato, Shingo

Kato, Shumei

Kato, Terufumi

Katodritou, Eirini

Katoh, Hiroyuki

Katsui, Kuniaki

Kattan, Michael

Katz, Steven

Katzenellenbogen, Rachel

Kaufman, Jonathan

Kaur, Kulvinder

Kawabe, Naoto

Kawada, Kenji

Kawaguchi, Kazunori

Kawaguchi, Takumi

Kawaguchi, Tomoya

Kawahara, Akihiko

Kawai, Akira

Kawai, K.

Kawaji, Hitomi

Kawakami, Hisato

Kawakami, Takeshi

Kawamoto, Teruya

Kawamura, Yuki I.

Kawasaki, Yoshihide

Kaye, Alan

Kaye, James

Ke, Jin

Ke, Chongwei

Ke, Yang

Keagan, Theresa

Keam, Bhumsuk

Keating, Nancy

Keessen, Marianne

Kehl, Kenneth

Keil, Kimberly

Keil, Vera

Keller, Charles

Keller, Karsten

Kelley, Katie

Kelley, Michael J.

Kelly, John D.

Kelly, Matthew

Kemeny, Nancy

Kemp, Graham

Kempen, Pauline

Kendall, Bradley

KenKnight, Bruce

Kenmotsu, Hirotsugu

Kenne Sarenmalm, Elisabeth

Kennecke, Hagen

Kennedy, Fiona

Kenny, Liz

Kent, Erin

Kent, Tara

Kenzik, Kelly

Kenzik, Kelly M.

Kerachian, Mohammad Amin

Kerckhove, Nicolas

Kerlikowske, Karla

Kern, Peter

Kerr, Stephen J.

Kerschbaumer, J.

Kesarwala, Aparna

Keschner, Yonatan

Kesler, Shelli R.

Kessel, Kerstin

Kesselheim, Aaron S.

Kesselheim, Jennifer

Kessler, Ronald

Keung, Emily

Key, Timothy

Key, Timothy J.

Keyomarsi, Khandan

Khakoo, Salim I.

Khalaf, Inaam

Khaled, Hussein

Khan Niazi, Muhammad Khalid

Khan, Fary

Khan, Gazala

Khan, Imran

Khan, Md. Wasim

Khan, Naazneen

Khan, Shah Zeb

Khan, Wasim

Khankari, Nikhil K.

Kharel, Sanjeev

Kharfan‐Dabaja, Mohamed

Kharrazi, Hadi

Khasraw, Mustafa

Khattry, Naveen

Khattry, Navin

Khaw, Kay‐Tee

Khazaee‐Pool, Maryam

Khetan, Vikas

Khoo, Athena Ming‐Gui

Khorana, Alok

Khosravi‐Farsani, Meysam

Khoury, Joseph

Khoury, Muin

Khubchandani, Jagdish

Khushalani, Nikhil

Kiang, Karrie M.Y

Kiani, Alexander

Kibriya, Muhammad

Kickingereder, Philipp

Kidd, Elizabeth A.

Kiemeney, Lambertus

Kieseker, Genevieve

Kiesslich, Tobias

Kiessling, Arndt‐Holger

Kietthubthew, Suparp

Kihara, Akio

Kikawa, Yuichiro

Kikkawa, Naoko

Kilander, Carl

Kim, Yeul‐Hong

Kim, Dong Wook

Kim, Beom Kyung

Kim, Byung Soo

Kim, Casey

Kim, Chang Gon

Kim, Choung‐Soo

Kim, Daniel W.

Kim, Dennis (Dong Hwan)

Kim, Do Young

Kim, Dong Wook

Kim, Duck‐Woo

Kim, Edward

Kim, Eun‐Kyung

Kim, Haeryoung

Kim, Haesook T.

Kim, Hak Jae

Kim, Hark

Kim, Hwiyoung

Kim, Hyeon Hoe

Kim, Hyeong Ryul

Kim, Hyeong Su

Kim, Hyeong‐Rok

Kim, Hyosong

Kim, Hyun

Kim, Hyun S.

Kim, Hyun Soo

Kim, Hyung Jun

Kim, Hyung Suk

Kim, Hyung‐Ho

Kim, Hyung‐Ryong

Kim, Inho

Kim, Jae‐Weon

Kim, Jean J.

Kim, Jee Soo

Kim, Jeong Goo

Kim, Jeong Hye

Kim, Jeongseon

Kim, Ji Eun

Kim, Jin Hyoung

Kim, Jin Seok

Kim, Jinyoung

Kim, Jong Gwang

Kim, Jong Man

Kim, Joo Sung

Kim, Joseph

Kim, Junyup

Kim, Katherine

Kim, Kevin

Kim, Ki‐Hun

Kim, Kun Hyung

Kim, Kwang‐Youn A.

Kim, Kyoung‐Mee

Kim, Kyu Sun

Kim, Kyubo

Kim, Kyung Keun

Kim, Michelle

Kim, Mi‐Sook

Kim, Nam Kyu

Kim, Richard

Kim, Sang Gyun

Kim, Sang Wun

Kim, Sanguk

Kim, Sang‐We

Kim, Se Hoon

Kim, Se Hyun

Kim, Seok‐Jun

Kim, Seon Hahn

Kim, Seung

Kim, Shana

Kim, Song Cheol

Kim, Soon Sun

Kim, stefano

Kim, Stefano

Kim, Sun Wook

Kim, Sung

Kim, Sung Hoon

Kim, Sung Joo

Kim, Sung‐Hwan

Kim, Sung‐Yong

Kim, Sun‐Whe

Kim, Tae Hyuk

Kim, Tae Hyun

Kim, Won Bae

Kim, Won Gu

Kim, Won Seog

Kim, Woo Ho

Kim, Woorim

Kim, Wun‐Jae

Kim, Yong Bae

Kim, Yong saeng

Kim, Yoon Jun

Kim, Yoonkeun

Kim, Youn Wha

Kim, Young Sun

Kim, Young Tae

Kim, Youngmee

Kim, Yu Jung

Kim, Yun Hak

Kimmelman, Alec

Kimondo, Faustini

Kimura, Shinya

Kimura, Tatsuo

Kimura, Tomoki

Kin, Toshifumi

Kinahan, Karen

King, Allison

King, Madeleine

King, Martin T.

King, Tricia

Kinoshita, Ichiro

Kinross, James

Kinuya, Seigo

Kipps, Thomas

Kiran, Nooti

Kirby, Anna

Kirchberger, Michael Constantin

Kircher, Sheetal

Kirchhof, Paulus

Kirchhoff, Anne

Kirov, Krassen

Kirschner‐Schwabe, Renate

Kirste, Simon

Kisely, Stephen

Kishan, Amar U.

Kishore, Uday

Kitagawa, Yuko

Kitahara, Cari M.

Kitai, Ryuhei

Kitajima, Kazuhiro

Kitano, Hiroyuki

Kitano, Shigehisa

Kitao, Akio

Kitatani, Kazuyuki

Kitchener, Henry

Kitpanit, Sarin

Kittles, Rick

Kiyokawa, Nobutaka

Kiyono, Tohru

Kiyota, Naomi

Kizaki, Masahiro

Kjaer, Susanne K.

Klaassen, Corné H. W.

Klaassen, Zachary

Klafke, Nadja

Klarenbeek, Bastiaan

Klassen, Anne

Klassen, Giseli

Klassen, Zach

Klatte, Tobias

Klauschen, Frederick

Kleespies, Axel

Klein, Hans‐Ulrich

Klein, Nicola

Kleinerman, Ruth A.

Klemp, Jennifer

Klempner, Samuel

Klikuchi, Yoshihiro

Klimstra, David

Kline, Cassie

Klingebiel, Thomas

Klingelhöfer, Doris

Klinkhammer‐Schalke, Monika

Klosky, James L.

Klumpers, Marije

Klusmann, Jan Henning

Kluza, Jérome

Knackstedt, Thomas J.

Knebel, Phillip

Knegt, Robert J. de

Knoop, Hans

Ko, Jennifer S.

Koay, Eugene

Kobayashi, Hatasu

Kobayashi, Hisataka

Kobayashi, Kunihiko

Kobayashi, Noritoshi

Kobayashi, Satoshi

Kobayashi, Tadao

Kobayashi, Takashi

Kobayashi, Tsuyoshi

Kobetz, Erin

Kobrin, Sarah

Kocak, B.

Koch, Anne

Kochenderfer, James N.

Kocher, Keith E.

Kochi, Mitsugu

Kodaira, Takeshi

Koebel, Martin

Koerber, Stefan

Koerkamp, Bas Groot

Koga, Yoshikatsu

Kogawa, Takahiro

Kogiso, Tomomi

Koh, Iemasa

Koh, Jaemoon

Koh, Kyung‐Nam

Koh, Youngil

Kohanbash, Gary

Kohei, Fujita

Köhler, Jens

Kohn, Christine

Kohno, Kenji

Kohno, Tadasu

Kohno, Takashi

Koie, Takuya

Koivikko, Mika

Kojima, Satoko

Kojima, Takashi

Kojima, Yasushi

Koksal, Hakan

Kolay, Sourav

Kole, Adam

Kolesnikova, Maria

Kolios, Christopher

Kollmannsberger, Christian

Kolodney, Joanna

Komaki, Ritsuko

Komaromy, Miriam

Komatsu, Shuhei

Komatsu, Tetsuya

Komatsu, Y.

Komiyama, Tomoyoshi

Komlodi‐Pasztor, Edina

Komohara, Yoshihiro

Komori, Takashi

Konda, Bhavana

Kondo, Shunsuke

Kondo, Takeshi

Kondo, Tetsuo

Kondziolka, Douglas

Kong, Chuize

Kong, Chung‐Yin

Kong, Daliang

Kong, Guang‐yao

Kong, Jin‐Liang

Kong, Jun

Kong, L.

Kong, Sun‐Young

Kong, Wei

Kong, Weimin

König, Marton

Konishi, Kazuo

Konishi, Yusuke

Konopleva, Marina

Konstantopoulou, Irene

Kontandreopoulou, Christina‐Nefeli

Koo, Ja Seung

Kooby, David

Koong, Albert C.

Koopman, M.

Kopetz, E. Scott

Kopnin, Pavel

Kopparapu, Prasad

Korc‐Grodzicki, Beatriz

Korde, Neha

Korenstein, Deborah

Korpanty, Grzegorz J.

Korszun, Ania

Kosaka, Takeo

Koschmieder, Steffen

Koshy, Matthew

Kostouros, Efthymios

Kosugi, S.

Kotani, Haruru

Kotronoulas, Grigorios

Kourelis, Taxiarchis

Kourie, Hampig Raphael

Kourlaba, Georgia

Kowalska, Aldona

Kowalski, Luiz Paulo

Kowalsky, L.

Koyama, Nobuyuki

Kozlowski, Piotr

Kozuki, Ryotaro

Krabbe, Laura‐Maria

Krag, Aleksander

Krahn, Murray

Krajden, Mel

Krakauer, Nir

Krakstad, C.

Kravitz, Richard

Kreimer, Aimee R.

Kreth, Friedrich‐Wilhelm

Kretzmer, Helene

Kridel, Robert

Kriplani, Anuja

Kris, Mark

Krishnamurthy, Sudha

Krishnan, Amrita

Krishnan, Bhavani

Kristensen, Bjarne W.

Kristensen, Gunnar

Kristiansen, Glen

Krivtsov, Andrei

Kroemer, Guido

Kroenke, Candyce

Kroiss, Matthias

Krok‐Schoen, Jessica

Kronenberg, Florian

Krug, Sebastian

Krumholz, Harlan M.

Kruse, Gina R.

Kruser, Timothy

Kryczek, Ilona

Krzyzanowska, Monika

Ku, Ja Hyeon

Kuan, Edward C.

Kuan, W. S.

Kuang, Ming

Kuba, Katharina

Kubicek, Greg

Kubik, Mark

Kubo, Makoto

Kubo, T.

Kubota, Kaoru

Kubota, Nobuko

Kuca, Kamil

Kuche, Kaushik

Kudo, Atsushi

Kudo, Masatoshi

Kudo, Shin‐ei

Kuerer, Henry

Kuhnt, Susanne

Kulkarni, Girish

Kullmann, Frank

Kulozik, Andreas

Kulshrestha, Arpita

Kuma, Akiko

Kumar, Alan Prem

Kumar, Arun

Kumar, Ashish

Kumar, Lalit

Kumar, Naresh

Kumar, Shaji K.

Kumar, Sunil

Kung, Che‐Pei

Kunimasa, Kei

Kunisaki, Chikara

Kuniyasu, Hiroki

Kuntz, Karen

Kunutsor, Setor K.

Kunwar, Amit

Kunz, Pamela

Kunzmann, Volker

Kuo, Chenk

Kuo, Sung‐Hsin

Kuranaga, Yuki

Kurata, Takayasu

Kurderer, Nicole

Kurian, Allison

Kurkowiak, Małgorzata

Kurnit, Katherine

Kuroda, Junya

Kürten, Cornelius

Kurtenbach, Stefan

Kurtovic‐Kozaric, Amina

Kuru, Bekir

Kuruvilla, John

Kurzer, Jason

Kurzrock, Razelle

Kushi, Lawrence

Kutyifa, Valentina

Kuwano, Hiroyuki

Kuznar‐Kaminska, Barbara

Kuzuya, Teiji

Kwak, Jin

Kwan, Kenny Yat Hong

Kwok, Hoi‐Hin

Kwok, Xinjin

Kwok, Yamkam

Kwon, Jae Hyun

Kwon, Jae‐Yung

Kwon, Minsuk

Kwong, Yok‐Lam

Kwong, Dora

Kwong, Yok Lam

Kyle, Robert

Kypta, Robert

Kypta, Robert M.

Kyrgiou, Maria

La Vecchia, Carlo

La, Elizabeth M.

Labaki, Chris

Laban, Simon

Labbe, Catherine

Labrie, Nanon

Lachowie, Curtis

Lacroix, Ludovic

Lacroix, Olivia

Lacy, Jill

Lad, Deepesh

Ladabaum, Uri

Ladanyi, Marc

LaDuca, Holly

Laelago, Tariku

Lafranconi, Alessandra

Lagergren, Pernilla

Laghi, Luigi

Laheru, Daniel

Lai, Alvina

Lai, Benjamin Chung Howe

Lai, Bin

Lai, Chao‐Lun

Lai, Chester

Lai, CL

Lai, Dongming

Lai, Mei‐Shu

Lai, Ming‐Derg

Lai, Paul BS

Lai, Quirino

Lai, Shicong

Lai, Xiao‐Ying

Lai, Yiming

Laios, Alexandros

Laird, Peter W.

Lairson, David

Lakhman, Y.

Lakomy, David

Lakshmanaswamy, Rajkumar

Lakshminarayan, Ramya

Lal, Girdhari

Lam, Vincent

Lam, Alfred K. ‐Y

Lam, David C. L.

Lam, Miranda

Lam, Thomas B. L.

Lam, Wai‐Ching

Lamb, Alastair

Lamb, Benjamin W.

Lamb, Edmund J.

Lambertini, Matteo

Lambrechts, Diether

Lampasona, Vito

Lampertico, Pietro

Lamy, Thierry

Lan, Dong

Lan, Qing

Lan, Xun

Lan, Yu

Lan, Yuan‐Tzu

Lan, Yuqing

Lanas, Angel

Lancia, A.

Lanciano, Rachelle

Landercasper, Jeffrey

Landy, Rebecca

Lang, Jinyi

Lang, Julie

Langan‐Evans, Carl

Langbecker, Danette

Langdon, Simon

Lange, Jane

Lange, Natascha M. de

Langenau, David M.

Langevin, Scott

Langhorst, Jost

Lankarani, Kamran B.

Lanktree, Matthew B.

Lantz, Anna

Lanza, Giovanni

Lao, Lixing

Lao, Xiang‐Ming

Laprise, Catherine

Larkin, James

Larson, David W.

Larsson, Anna‐Maria

Larsson, Catharina

Larsson, Susanna

Larue, Ruben T. H. M.

Lash, Timothy

Latonen, Leena

Lau, Clara

Lau, Francis

Lau, Wan Yee

Lau, Wan‐Yee

Lau, Yun‐Fai C.

Laubach, Jacob P.

Launoy, Guy

Laurberg, Søren

Lauren, Fontana

Laurent, Camille

Lavrenkov, Konstantin

Law, Calvin

Law, Chi Kin

Law, Matthew

Law, Wai‐Lun

Lawler, Sean

Lawlor, Peter

Lawrenson, Ross

Lawrie, Theresa A.

Lawson, Michelle

Lazar, Alex

Lazar, Ann A.

Lazarus, Hillard M.

Le Cesne, Axel

le Coutre, P. D.

Le Gouill, Steven

Le Guin, Claudia

Le Rhun, Emilie

Le, Linda Van

Leach, Corinne

Leal, Viola

Leandersson, Karin

Lebbe, Celeste

Lebeck Lee, Cody

LeBlanc, A. de Moreno de

LeBlanc, Erin S.

LeBlanc, Thomas

LeCun, Yann

Lee, Hae‐Ock

Lee, Agnes

Lee, Alexander

Lee, Bae Hoon

Lee, Catherine

Lee, Chang Geol

Lee, Chee

Lee, Chia‐Hwa

Lee, Cho‐Rok

Lee, Christoph

Lee, Dong Gun

Lee, Dong‐Gun

Lee, Dongjun

Lee, Eun Jig

Lee, Gao

Lee, Garrick D.

Lee, Grace

Lee, Hak Jong

Lee, Han Chu

Lee, Han Hong

Lee, Hans

Lee, Hansang

Lee, Ho

Lee, Ho Sup

Lee, Hsu‐Tung

Lee, Hye Seung

Lee, Hye‐Seung

Lee, Hyunju

Lee, I‐Cheng

Lee, Jae Cheol

Lee, Jae Jun

Lee, James

Lee, Jang‐Ming

Lee, Jeeyun

Lee, Je‐Hwan

Lee, Jeong Eon

Lee, Jeong Hyun

Lee, Jeong‐Hoon

Lee, Jeong‐Ok

Lee, Ji Ye

Lee, Jong Hoon

Lee, Ju‐Yeun

Lee, Juyong

Lee, Ki Hyeong

Lee, Kwang‐Won

Lee, Kyung Jong

Lee, Kyung Soo

Lee, Kyuwan

Lee, Lawrence

Lee, Mangyeong

Lee, Min Woo

Lee, Nancy Y.

Lee, Ping‐Chin

Lee, Pyng

Lee, Rheun‐Chuan

Lee, Sang Soo

Lee, Sang‐Soo

Lee, Sang‐Woo

Lee, Seok

Lee, Seung Jin

Lee, Seung Soo

Lee, Shin Yup

Lee, Shin‐Wha

Lee, Soo Chin

Lee, Sung

Lee, Sung Koo

Lee, Victor Ho Fun

Lee, WonChul

Lee, Woo Jin

Lee, Yew Kong

Lee, Yong

Lee, Yong Jae

Lee, Yong‐Jae

Lee, Young‐Kyun

Leelahavanichkul, Asada

Leelawat, Kawin

Leen, Liao

Leenders, William

Lees, Jacqueline A.

Leeuw, Irma M. Verdonck‐de

Lefevre, Jeremie

Legge, Francesco

Legood, Rosa

Leguisamo, Natalia

Lehrer, Eric

Lehrman, David

Lei, Mingxing

Lei, Shu‐Feng

Lei, Wenbin

Lei, Zhe

Leibowitz, Raya

Leighl, Natasha

Leijenaar, Ralph T. H.

Leite, Valeriano

Leitzmann, Michael

Lemoine, Antoinette

Leng, Zhengwei

Leng, Guangxian

Leng, Xuefeng

Lennon, Anne Marie

Lentsch, Eric J.

Lenz, Heinz

Lenz, Heinz‐Josef

Leonard, John

Leone, Gustavo

Leone, José

Lepique, Ana Paula

Leprieur, Etienne Giroux

Leprivier, Gabriel

Lerner, Seth P.

Lerro, Catherine C.

Lertbutsayanukul, Chawalit

Leshno, Moshe

Lester‐Coll, Nataniel H.

Letai, Anthony

Leung, Euphemia

Leung, Gilberto Ka‐Kit

Leung, Wai Keung

Leval, Laurence de

Levesque, Mitchell P..

Levey, Andrew S.

Levi, Francis

Levine, Deena

Levis, Mario

Levy, Antonin

Levy, Douglas E.

Levy, Jerrold H.

Lew, Michael W.

Lewin, Alan

Lewin, Jeremy

Lewis, Denise

Lewis, John H.

Lewis, Mark

Lewis‐Thames, Marquita

Leyh‐Bannurah, Sami‐Ramzi

Li, Xiaoying

Li li., Pui‐Kai

Li, Baohua

Li, Baosheng

Li, Bingkun

Li, Binglu

Li, Bo

Li, Boqi

Li, Cai

Li, Chao

Li, Chaofeng

Li, Chen

Li, Chengcheng

Li, Chengwen

Li, Chengyun

Li, Chong

Li, Chuanming

Li, Chuanxing

Li, Chunquan

Li, Chunyan

Li, Cong

Li, Cui

Li, Dan

Li, De‐Jia

Li, Deming

Li, Depei

Li, Dinggang

Li, Donghui

Li, Fan

Li, Fang

Li, Faping

Li, Fazhan

Li, Gong‐Hui

Li, Guang

Li, Guanghui

Li, Guangyu

Li, Guanzhang

Li, Guojun

Li, Guoxin

Li, Hailiang

Li, Hai‐Liang

Li, Haixin

Li, Haobo

Li, Haojiang

Li, Haoran

Li, Hao‐yong

Li, Hefei

Li, Hengli

Li, Hongdan

Li, Honglin

Li, Hongwei

Li, Hongyu

Li, Hua

Li, Hui

Li, Huili

Li, Ji

Li, Jia

Li, Jian

Li, Jiancheng

Li, Jianhao

Li, Jianpeng

Li, Jianxue

Li, Jing

Li, Jingao

Li, Jin‐Gao

Li, Jingwen

Li, Jinluan

Li, Jinyun

Li, Juan

Li, Jun

Li, Kai

Li, Kang

Li, Kejia

Li, Kezhen

Li, Kunkun

Li, Kun‐Kun

Li, Laisheng

Li, Lei

Li, Le‐Qun

Li, Li

Li, Liang

Li, Lifeng

Li, Lingchang

Li, Lingchao

Li, Lun

Li, Madeline

Li, Man

Li, Mei

Li, Menghui

Li, Mengmeng

Li, Mengshi

Li, Mengyuan

Li, Min

Li, Ming

Li, Mingshan

Li, Min‐Min

Li, Na

Li, Nan

Li, Ni

Li, Ning

Li, Pan

Li, Pei‐Nan

Li, Qi

LI, QI

Li, Qian

Li, Qiang

Li, Qiao‐Qiao

Li, Qing

Li, Qingguo

Li, Qiu

Li, Qiyuan

Li, Qun

Li, Roger

Li, Rong

Li, Rutian

Li, Shaoying

Li, Shau‐Hsuan

Li, shengjie

Li, Shengjun

Li, Shengping

Li, Shijun

Li, Shikang

Li, Shuai

Li, Shuben

Li, Shufeng

Li, Sinead

Li, Sin‐Syue

Li, Tengcheng

Li, Ting

Li, Tong

Li, Tsai‐Chung

Li, Tuan‐Jie

Li, Wang

Li, Wei

Li, Wei Li

Li, Wenbo

Li, Wen‐Fei

Li, Wen‐Xing

Li, Xia

Li, xiancheng

Li, Xiang

Li, Xiangchun

Li, Xianglan

Li, Xiangping

Li, Xianming

Li, Xiao

Li, Xiaobo

Li, Xiaodong

Li, Xiaoyou

Li, Xingchen

Li, Xingrui

Li, Xinxing

Li, XiuShen

Li, Xuebing

Li, Xuejun

Li, Xueli

Li, Xue‐Nong

Li, Xue‐Song

Li, Xu‐qi

Li, Yafang

Li, Yajuan

Li, Yan

Li, Yang

Li, Yangle

Li, Yangqiu

Li, Ye‐Xiong

Li, Yimei

Li, Yin

Li, Yingbin

Li, Yingshan

Li, Yirong

Li, Yitong

Li, Yong

Li, Yongning

Li, Yongsheng

Li, Yueyao

Li, Yu‐hong

Li, Yujuan

Li, Yuling

Li, Yu‐ming

Li, Yuqiang

Li, Zai‐bo

Li, Zhanzhan

Li, Zhaoping

Li, Zhen

Li, Zheng

Li, Zhi‐Cheng

Li, Zhi‐Ming

Li, Zhi‐Qiang

Li, Zhiqin

Li, Zhiyong

Li, Zhongrong

LI, Zhouxiao

Li, Zhuang

Liabsuetrakul, Tippawan

Lian, Jean

Liang, Aibin

Liang, Baosheng

Liang, Changhong

Liang, Chao

Liang, Chaofeng

Liang, Chun

Liang, Cuishan

Liang, Geyu

Liang, Hailun

Liang, Han

Liang, Helen

Liang, Hua

Liang, Jiaqi

Liang, Jie‐ying

Liang, Jing

Liang, Li

Liang, Liming

Liang, Shaobo

Liang, Shide

Liang, Ting‐Bo

Liang, Tong

Liang, Weifeng

Liang, Wenhua

Liang, Xiaoliang

Liang, Yingjie

Liang, Yizhi

Liang, Zi‐Qian

Liao, Chengyu

Liao, Joseph C.

Liao, Lian‐Di

Liao, Linda M.

Liao, Ning

Liao, Qi

Liao, Quan

Liao, Rui

Liao, Wangjun

Liao, Xiwen

Liao, Yongde

Liao, Zhongxing

Liaw, Yun‐Fan

Libra, Massimo

Licht, Sofie de Fine

Lichtensztajn, Daphne

Lichtenthal, Wendy

Licitra, Lisa

Licitra, LIsa

Lie, Hanne C.

Liebisch, Gerhard

Liebovitz, David M.

Liefers, Gerrit‐Jan

Lien, Tonje G.

Lieske, John

Lieu, Christopher

Ligibel, Jennifer

Lijfering, Willem

Lim, Bora

Lim, Chaeseong

Lim, Do Hoon

Lim, Geok

Lim, Hui Jun

Lim, Jong‐Baeck

Lim, Joon Seok

Lim, Ka Young

Lim, Michael

Lim, Renelle

Lim, Soon Thye

Lim, Taekyu

LIM, Tian‐Zhi

Lim, Young‐Suk

Lin, Ai‐Hua

Lin, Bei

Lin, Chang‐Sheng

Lin, Chenyu

Lin, Chi

Lin, Chien‐Chung

Lin, Chung‐Ying

Lin, Chunhua

Lin, Chunqing

Lin, Dang

Lin, De‐Chen

Lin, Dongxin

Lin, Fan

Lin, Han‐Chieh

Lin, Hao‐Yu

Lin, Hengzhou

Lin, Huan‐Xin

Lin, Huipin

Lin, Jessica

Lin, Jian

Lin, Jing

Lin, Jingrong Lin

Lin, Jiumao

Lin, Ju‐Li

Lin, Junzhong

Lin, Kai‐Yuan

Lin, Li

Lin, Lili

Lin, Mingzhi

Lin, Peggy

Lin, Po‐Han

Lin, Qian

Lin, Qin

Lin, Qizhan

Lin, Rui

Lin, Shao

Lin, Sheng‐Fung

Lin, Shih‐Min

Lin, Steven

Lin, Tianxin

Lin, Wei

Lin, Xiangan

Lin, Xiaojun

Lin, Xiao‐Jun

Lin, Xiaoning

Lin, Xu

Lin, Yansong

Lin, Yao

Lin, Yazhou

Lin, Ying

Lin, Yingsong

Lin, Yun Pei

Lin, Yung‐Chang

Lin, Yu‐Tsai

Lin, Zhiguo

Lindenauer, Peter K.

Linder, Nicolas

Linderholm, Barbro

Lindor, Noralane M.

Lindquist, David

Lindstrom, Annika K.

Ling, Hong

Ling, Sunbin

Ling, Zhi‐qiang

Ling, Zhi‐Qiang

Ling, Zhou‐Gui

Linn, Sabine

Linnebacher, Michael

Linton, Kim M.

Liou, Douglas

Lipitz‐Snyderman, Allison

Lipkin, Steven

Lippman, Scott

Lirdprapamongkol, Kriengsak

List, Alan F.

Litton, Jennifer K.

Liu, Angela Hui‐Chia

Liu, Baorui

Liu, Ben

Liu, Bian

Liu, Bing

Liu, Bing‐Ya

Liu, Bolin

Liu, Chang

Liu, Changan

Liu, Changzheng

Liu, Chen

Liu, Chengxin

Liu, Chen‐Hua

Liu, Cunzhi

Liu, Dai

Liu, Daihong

Liu, Dapeng

Liu, Delong

Liu, Deruo

Liu, Erqi

Liu, Fayu

Liu, Fen

Liu, Fengyong

Liu, Gao

Liu, Geoffrey

Liu, Guangwei

Liu, Guoyuan

Liu, Han

Liu, Hanyang

Liu, Hao

Liu, HaoSheng

Liu, Hong

Liu, Hongde

Liu, Hongfei

Liu, Houbao

Liu, Huanliang

Liu, Ji

Liu, Jia

Liu, Jiameng

Liu, Jiaqi

Liu, Jiayong

Liu, Jie

Liu, Jihong

Liu, Jue

Liu, Jun

Liu, Junhua

Liu, Juying

Liu, Kai

Liu, Kaichao

Liu, Kan

Liu, Kuangzheng

Liu, L.

Liu, Lei

Liu, Leping

Liu, Liang

Liu, Ling Tian

Liu, Lingxiang

Liu, Linhua

Liu, Lizhi

Liu, Lizhou

Liu, Meina

Liu, Meng‐Zhong

Liu, Michael

Liu, Min

Liu, Ming

Liu, Mingming

Liu, Mingyao

Liu, Minling

Liu, Mulin

Liu, Na

Liu, Ning

Liu, Ningbo

Liu, Panpan

Liu, Peishu

Liu, Peng

Liu, Ping

Liu, Qi

Liu, Qiang

Liu, Qingguang

Liu, Qiuping

Liu, Ran‐yi

Liu, Rong

Liu, Sai‐Lan

Liu, Shaohua

Liu, Shengchun

Liu, Shengwei

Liu, Shing‐Hwa

Liu, Shuaihong

Liu, Shumei

Liu, Shun

Liu, siping

Liu, Songbai

Liu, Songran

Liu, Songyang

Liu, Stephen

Liu, Tao

Liu, Tongjun

Liu, Wanli

Liu, Wei‐Hsiu

Liu, Wen‐Shan

Liu, X. Shirley

Liu, Xiangli

Liu, Xiangqi

Liu, Xiaoan

Liu, Xiaofeng

Liu, Xiaoguang

Liu, Xiaoming

Liu, Xiaoqi

Liu, Xiaowen

Liu, Xiaoxue

Liu, Xing

Liu, Xinpeng

Liu, Xinru

Liu, Xuejiao

Liu, Y.

Liu, Yang

Liu, Yanjun

Liu, Yan‐ling

Liu, Yao

Liu, Yibing

Liu, Yi‐Fei

Liu, Yi‐Nan

Liu, Yong

Liu, Yongda

Liu, Yu

Liu, Yufei

Liu, Yun

Liu, Yunpeng

Liu, Zaiyi

Liu, Zelong

Liu, Zeming

Liu, Ze‐Xian

Liu, Zeyi

Liu, Zhao‐Qian

Liu, Zheng

Liu, Zhenzhen

Liu, Zhifeng

Liu, Zhi‐gang

Liu, Zhihua

Liu, Zhiqiang

Liu, Zhiwei

Liu, Zhixiong

Liu, Zhong

Liu, Zhuang

Liu, Zhuqing

Liu, Zuqiang

Livhits, Masha J.

Livieris, Ioannis

Livingston, J.

Livingston, Patricia

Livyatan, Ilana

Ljungberg, Borje

Llanos, Adana

Lloberas, Nuria

Lo Re, Vincent

Lo, Chung‐Ming

Lo, Hui‐Wen

Lo, Kwok‐Wai

Lo, Regina Cheuk‐Lam

Lo, Simon S.

Lo, Y. M. Dennis

Locati, Laura

Loch, Tillmann

Loeb, Mark

Loehrer, Elizabeth

Logan, Brent

Logan, Jessica

Loggers, Elizabeth

Loghavi, Sanam

Logie, Natalie

Loh, Kah Poh

Løhmann, Ditte

Lohmann, Philipp

Löhr, Johannes‐Matthias

Lomberk, Gwen

London, Daniel A.

Long, Georgina V.

Long, Haixia

Long, Hao

Long, Jirong

Long, Yaqiu

Longatto‐Filho, Adhemar

Longhi, Alessandra

Lonial, Sagar

Looijenga, Leendert

Lookstein, Robert A.

Loonen, J.J

Loong, Herbert

Loosen, Sven

Lopa, Samia

Lopes, Gilberto

Lopes da Silva, Laercio

Lopes, Daiana Silva

Lopez, Alan D.

Lopez, Evangelina

Lopez, Juanita S.

Lopez‐Martin, Jose A.

Loprinzi, Charles

Loquai, Carmen

Lordick, Florian

Loree, Jonathan

Lorem, Geir

Lorgelly, Paula

Lorigan, Paul

Loriot, Yohann

Lossos, Izidore S.

Lotan, Yair

lotfinejad, Parisa

Lotze, Michael

Lou, Neng

Lou, Pei Jen

Lou, Weiyang

Lou, Wenhui

Lou, Yanyan

Louis, Daniel Z.

Louis, Petra

Loupakis, F.

Loupakis, Fotios

Lovat, Francesca

Lovett, Josep M.

Lovšin, Ema

Low, Donald

Lowe, Kimberly

Lowy, Andrew M.

Lozano, Rafael

Lu, Bin

Lu, Bingjian

Lu, Congxiao

Lu, Donghao

Lu, Fengmin

Lu, Guangming

Lu, Hongguang

Lu, Hongtao

Lu, Hu

Lu, Hung‐I.

Lu, Huoquan

Lu, Jiachun

Lu, Jiade

Lu, Jian

Lu, Jianping

Lu, Jingli

Lu, Jin‐Jian

Lu, Jun

Lu, Kai

Lu, Lingeng

Lu, Qingchun

Lu, Renquan

Lu, Rongl

Lu, Sen

Lu, Shelly

Lu, Shouliang

Lu, Shun

Lu, Taixiang

Lu, Tianzhu

Lu, Wei

Lu, Xiaojie

Lu, You

Lu, Yuangang

Lu, Yuntao

Lu, Zhenhai

Lu, Zhenzhang

Lu, Zhihao

Lucas Jr., John

Lucas, Aimee L.

Lucci, Anthony

Ludwig, Michelle

Luffer‐Atlas, Debra

Lugnier, Celine

Lugowska, Iwona

Ługowska, Iwona

Lui, Su

Lukaski, Henry

Lukaszuk, Robert F.

Luke, Jason

Lukong, Kiven Erique

Luminari, S.

Lun, Liming

Lunet, Nuno

Lunning, Matthew

Luo, Baoming

Luo, Chunli

Luo, Dakui

Luo, Danmeng

Luo, Dong‐Hua

Luo, Guanghong

Luo, Hao

Luo, Hui

Luo, Jiing‐Chyuan

Luo, Jun

Luo, Junhang

Luo, Lin

Luo, Minglei

Luo, Nan

Luo, Peng

Luo, Qingquan

Luo, Sheng

Luo, Shixian

Luo, Shuhong

Luo, Tianqi

Luo, Xiao

Luo, Yongwen

Lupak, Oleksandra

Lupichuk, Sasha

Lupo, Philip

Luthra, Rajyalakshmi

Lutz, Philipp

Luu, Hung

Lu‐Yao, Grace

Luzzago, Stefano

Lv, Changlong

Lv, Tangfeng

Lv, Xiaohong

Lykke, J.

Lyko, Frank

Lyman, Gary

Lynch, Conor C.

Lynge, Elsebeth

Lyon, Robert

Lyu, Hui

Lyu, Jianxin

Lyu, Jing

Lyu, Jun

Lyu, Ning

M. El‐Haggar, Sahar

Ma, Aixia

Ma, Chao

Ma, Chaoqun

Ma, Cynthia

Ma, De‐hua

Ma, Dong

Ma, Haochuan

Ma, Hong

Ma, Hongxia

Ma, Hui‐Yan

Ma, Jiachi

Ma, Jianlin

Ma, Ji‐chun

Ma, Jie

Ma, Jun

Ma, Liansheng

Ma, Rong

Ma, Shaohui

Ma, Shenglin

Ma, Wen‐Lung

Ma, Xiang

Ma, Xiao‐Chi

Ma, Xiaoli

Ma, Xiaotu

Ma, Xuelei

Ma, Xuezhen

Ma, Yanlei

Ma, Yifei

Ma, Ying

Ma, Zhiyao

Ma, Zhongliang

Ma, Zhuo

Maatouk, Imad

Macchia, Gabriella

Macedo, G.

Macedo, Filipa

Machalek, Dorothy A.

Machida, Yuichi

Macia, Sonia

Macias, Rocio I. R.

Mack, Jennifer

Mackall, Crystal L.

Mackay, Helen

Mackay, Tara

Maclean, Elizabeth

MacMillan, Connor

Maculaitis, Martine

Macuzic, Ivana

Madabhushi, Anant

Madan, Ankit

Mader, Luzius

Madhusudan, Srinivasan

Madney, Youssef

Madrigal‐Burgaleta, Ricardo

Maeda, Hideki

Maeda, Hiromichi

Maeda, Osamu

Maemondo, Makoto

Maeng, Chi Hoon

Maggard Gibbons, Melinda

Magnano, Laura

Magne, Nicolas

Magnusson, Maria K.

Maguire, Rebecca

Mahabir, Somdat

Mahajan, Vandana

Mahale, Parag

Mahapatra, Manoranjan

Mahé, Isabelle

Maher, Eamonn

Maheswari, Amita

Mahipal, Amit

Mahmood, Safrunnisa

Mahmoud, Omar

Mai, Antonello

Mai, Hai‐Qiang

Maiello, Evaristo

Mailankody, Sham

Mailankody, Sharada

Mainprize, James Gordon

Maisonneuve, Patrick

Maithel, Shishir

Maity, Ranjan

Majhail, Navneet

Mak, Lung Yi

Mak, Nai Ki

Makarem, Nour

MAKHANI, SARAH

Makhnoon, Sukh

Maki, Robort

Makita, Chiyoko

Malagola, Michele

Malagon, Talia

Malapelle, Umberto

Malavasi, Fabio

Małecka‐Wojciesko, Ewa

Maleki, Zahra

Malhotra, Chetna

Malhotra, Hemant

Malhotra, Jyoti

Malhotra, Pankaj

Malik, Astha

Malik, Prabhat S.

Malka, David

Malleo, Giuseppe

Malo, Teri L.

Malone, Eoghan

Maloney, David

Malouf, Gabriel

Malouf, Gabriel G.

Malumbres, Marcos

Mammadov, Emin

Mamo, Zerihun Berhanu

Mamounas, Eleftherios P.

Manabe, Osamu

Manatakis, Dimitrios K.

Manchanda, Ranjit

Mandal, Amrita

Mandala, Mario

Mandati, Vinay

Manderbacka, Kristiina

Manegold, Christian

Manfredi, Angelo

Mangana, Joanna

Mangia, Anita

Mangone, Lucia

Maniadakis, Nikos

Maniar, Kruti P.

Manikandan, Dhanushkodi

Mannaerts, Christophe K.

Manne, Upender

Manning, H. Charles

Mano, Max

Manoharan, Shanmugam

Mansfield, Aaron S.

Mansfield, Kyle D.

Manson, JoAnn

Mansour, Bassel

Mansour, John

Mao, Jia‐Ding

Mao, Jianhua

Mao, Jun

Mao, Kai

Mao, Li

Mao, Min

Mao, Minjie

Mao, Qinsheng

Mao, Xiangming

Mao, Yanping

Mao, Yilei

Mao, Yuan

Marabese, Mirko

Marangoni, Elisabetta

Marano, Luigi

Marasco, Giovanni

Marcato, Paola

Marchak, Jordan Gilleland

Marchegiani, Giovanni

Marchesi, Federica

Marchesi, Francesco

Marchetti, Lucia

Marchevsky, Alberto M.

Marchioni, M.

Marco, Ladetto

Mardinoglu, Adil

Mardis, Elaine

Mareschal, Julie

Margolis, Karen

Margulis, Vitaly

Marijnen, Corrie A. M.

Marini, Simone

Marino, Jennifer L.

Marinovich, Luke

Marins, Mozart

Mariotto, Angela

Mariya, Tasuku

Markar, Sheraz

Markowetz, Florian

Markowitz, Lauri E.

Marks, Hendrik

Marks, Lawrence

Marley, Andrew R.

Marra, Marco

Marrelli, Daniele

Marrer, Emilie

Marschalek, R.

Marschner, Norbert

Marshall, Deborah

Marshall, John

Marta, Gustavo

Martelli, Maurizio

Martens, Joost

Martin Batista, Silvia

Martin, Alberto

Martin, Alexandra

Martin, Anne‐Celine

Martin, Chelsea K.

Martin, Jennifer H.

Martin, Michelle

Martin, Miguel

Martin, Robert

Martin, Thomas

Martín‐Broto, Javier

Martinez, Maria

Martínez‐Chantar, María Luz

Martínez‐Pérez, Carlos

Martini, Maurizio

Martino, Massimo

Martin‐Orozco, Elena

Maru, Girish

Maruyama, Dai

Marx, Alexander

Masai, Kyohei

Masaki, So

Masaki, Tsutomu

Mascarenhas, John

Mascaux, Celine

Mascitti, Marco

Masi, Gianluca

Masih‐Khan, Esther

Mason, Casey

Masoudi‐Nejad, Ali

Massad, Leslie S.

Massari, Francesco

Massarweh, Nader

Massion, Pierre P.

Master, Viraj

Masters, Thao

Mastropasqua, Mauro

Mastropasqua, Rodolfo

Masuda, Norikazu

Matboli, Marwa

Materin, Miguel

Mathevet, Patrice

Mathew, Porunelloor

Mathur, Karan

Mathy, Jon

Matias‐Guiu, Xavier

Mato, Jose

Matos, L.

Matos, Leandro Luongo

Matosevic, Sandro

Matsubara, Daisuke

Matsuda, Koichi

Matsuda, Tomohiro

Matsui, Hidenori

Matsukuma, Susumu

Matsumoto, Koji

Matsumoto, Shigemi

Matsumoto, Toshihiko

Matsumura, Itaru

Matsumura, Noriomi

Matsumura, Yasuhiro

Matsunaga, Shigeo

Matsuo, Keitaro

Matsuo, Koji

Matsuura, Norihiro

Matsuyama, Hideyasu

Matsuzaka, Masashi

Mattavelli, Davide

Matthias, Julia

Mattiello, Amalia

Mattiucci, Gian Carlo

Matulonis, Ursula

Maugeri, Andrea

Maugeri, Marco

Maugeri‐Saccà, Marcello

Maughan, Tim

Maula, Sanna

Maura, Francesco

Maura, Geric

Maurer, Shanthi

Maurer, Tobias

Mauro, Michael

Mavridis, Konstantinos

May, Anne

May, Brian

May, Peter

May, Taymaa

Mayden, Kelley D.

Mayer, Arnulf

Mayer, Deborah K.

Mayinger, Michael

Mayland, Catriona

Mazibuko, Thandeka

Mbuagbaw, Lawrence

McAleer, Mary Frances

McArthur, Grant

McArthur, Grant A.

McBride, Mary L.

McCaffrey, Nikki

McCarter, Kristen

McCaw, Tyler

McClanahan, Terrill K.

McCleary, Nadine

McClellan, Mark

McCray, Alexa

McCullough, Marji

McDermott, David

McDermott, Michael W.

McDonald, Brenna C.

McDonald, Jasmine

McDonald, Kerrie L.

McDougall, Jean

McFarland, Daniel Curtis

McGlynn, Katherine

McGrail, Kimberlyn

McGregor, Iain

McGrowder, Donovan

McGuire, Colleen

McGuirk, Joseph

McIntire, Patrick J.

McKay, James

Mckay, Rana

McKenzie, Fiona

McKinn, Shannon

McLish, Laten

McMillan, Diana

McMullin, Mary

McMurray, A.

McNamara, Mairead

McNamara, Mairead G.

McNeer, Jennifer L.

McTyre, Emory

McWilliams, Robert

Meade, Cathy

Mechoulam, Raphael

Mechta‐Grigoriou, Fatima

Meckes, David

Medeiros, L.Jefferey

Medhi, Bikash

Meeker, Caitlin

Mego, M.

Megwalu, Uchechukwu

Megwalu, Uchechukwu C.

Mehanna, Hisham

Mehdipour, Fareshteh

Mehdizadeh, hamed

Mehnert, Anja

Mehnert, Janice

Mehnert, Janice M.

Mehrolhassani, Mohammad Hossein

Mehrotra, Ravi

Mehta, Anuj B.

Mehta, Sunali

Mei, Heng

Mei, Shenglin

Mei, Zubing

Meier, Friedegund

Meine, Timo

Meirovitz, Amichay

Meiser, Bettina

Meissner, Markus

Mejean, Arnaud

Mejia‐Arangure, Juan Manuel

Mekkawy, Ahmed

Melaku, Yohannes Adama

Melcher, Alan

Melchior, Nicole

Melero, Ignacio

Melichar, Bohuslav

Melin, Beatrice

Melisi, Davide

Mendoza, Tito

Menegaux, Florence

Menendez, Carolyn

Meng, Jialin

Meng, Lirong

Meng, Su

Meng, Tong

Meng, Xiangjiao

Meng, Xiangkai

Meng, Xiang‐Wei

Meng, Xiang‐Yu

Meng, Xue

Meng, Yue

Menon, Manoj B.

Menon, Usha

Mensah, George A.

Mercadante, Sebastiano

Mercieca‐Bebber, Rebecca

Merdan, Selin

Merigo, Jose M.

Merkelbach‐Bruse, Sabine

Merks, Johannes H. M.

Merle, Philippe

Merli, Michele

Mertens, Ann C.

Merz, Lauren

Mesa, Ruben

Mesa, Ruben A.

Mesker, W.E

Messiou, Christina

Metro, Giulio

Metzeler, Klaus

Metzeler, Klaus H.

Meyer, Alberto

Meyer, Anne‐Marie

Meyer, Ashley

Meyer, Christoph

Meyer, Larissa

Meyers, Bryan

Meyers, Rebecka

Meza, Rafael

Meza‐Menchaca, Thuluz

Mezquita, Laura

Mezzanzanica, Delia

Mg, Xiangdi

Mi, Xinlei

Mi, Yingchang

Mian, Hira

Mian, Omar

Miao, Beiping

Miao, Dong‐liu

Miao, Hongming

Miao, Xiangyang

Miao, Xiang‐Yang

Miao, Xiaoping

Miao, Yanwei

Miao, Yi

Miaskowski, Christine

Micaux Obol, Claire

Michael, Natasha

Michaelso, M. Dror

Michaud, Dominique

Michel, Gisela

Michelis, Fotios

Michlewski, Gracjan

Michon, Jean

Michos, Erin

Micozzi, Alessandra

Middeldorp, Jaap

Middleton, William

Midorikawa, Yutaka

Migliorini, Denis

Migowski, Arn

Mikami, Kazuya

Mikesell, Lisa

Miki, Yasuhiro

Miki, Yuichiro

Mikołajczyk, Tomasz

Mikoshiba, Takuya

Miksad, Rebecca A.

Milano, Michael

Milano, Michael Thomas

Milbury, Kathrin

Milella, Michele

Milicic, Biljana

Millar, Morgan

Miller, Amanda

Miller, Anthony

Miller, C. Ryan

Miller, C.A

Miller, Crispin

Miller, David

Miller, Jacqueline

Miller, Kimberly

Miller, Robert

Miller, Vincent A.

Mills, Anne M.

Mills, Gordon B.

Mills, Ian

Mills, James L.

Mimori, Koshi

Min, Chang‐Ki

Min, Kyung Hoon

Min, Le

Minami, Seigo

Minami, Yosuke

Minamiguchi, Sachiko

Minamiya, Yoshihiro

Minato, Koichi

Minaya, Miguel

Minchenko, Oleksandr

Minervini, Andrea

Minig, Lucas

Minin, Vladimir

Minna, John

Mino‐Kenudson, Mari

Minsky, Bruce

Mir, Carmen

Mir, Maria

Mir, Olivier

Mir, Snober

Mirabeau‐Beale, Kristina

Mirgh, Sumeet

Mirshafiey, Abbas

Mirza, Mansoor R.

Mirza, Mansoor Raza

Mishra, Gita D.

Mishra, Mark

Mishra, Nitish Kumar

Misra‐Hebert, Anita

Missel, Malene

Mistro, Annarosa Del

Mita, Monica M.

Mitani, Sohei

Mitchell, Duane

Mitchell, Tara

Mitin, Timur

Mitra, Abhisek

Mitrovic, Bojana

Mittal, Navakirti

Mittal, Vivek

Mittempergher, Lorenza

Mittmann, Nicole

Miura, Koichi

Miura, Satoru

Miyakawa, Akifumi

Miyake, Hideaki

Miyamoto, Takeshi

Miyashita, Mitsunori

Miyata, Naoteru

Miyauchi, Akira

Miyauchi, Eisaku

Miyawaki, Yutaka

MIyazawa, Masaki

Miyazono, Kohei

Miyoshi, Hiroaki

Miyoshi, Norikatsu

Mizoe, Jun‐etsu

Mizokami, Atsushi

Mizushima, Noboru

Mlak, Radoslaw

Mlak, Radosław

Mo, Hao‐Yuan

Mo, Luxia

Mo, Qianxing

Mo, Xiaofei

Moadel, Alyson

Moazezi, Zoleika

Mocellin, Simone

Modest, Dominik P.

Modi, Parth

Modrek, Aram

Mody, Kabir

Moehler, Markus

Moenig, Stefan

Moertl, Simone

Moffatt, Miriam F.

Moghaddam, Seyed Javad

Mognato, Maddalena

Mohamed, ASR

Mohamed, Mervat S.

Mohamed, Rela

Mohammadpour, Hemn

Mohammed, Altaf

Mohammed, Kahee

Mohammed, Kahee A.

Mohan, Babu

Mohan, Giridhar

Mohan, Helen M.

Mohan, Viswanathan

Mohindroo, Chirayu

Mohiuddin, Md

Mohler, James

Mohr, Peter

Mohty, Mohamad

Moineddin, Rahim

Moise, Pierre

Mok, Tony

Mokdad, Ali

Molassiotis, Alex

Molderings, Gerhard

Molena, Daniela

Molife, Lulama R.

Molinaro, Jessica

Moll, Herwig

Möller, Birgit

Møller, Pål

Molnar, Bela Peter

Momose, Syuji

Moncayo, Gerald

Mondul, Alison

Monelli, Filippo

Monie, Daphne

Monje, Michelle

Monnereau, Alain

Monreal, Manuel

Montagna, Emilia

Montagut, Clara

Monteith, Gregory

Montella, Maurizio

Montemurro, Filippo

Montero‐Conde, Cristina

Montesinos, Pau

Montesinos‐Rongen, Manuel

Montillo, Marco

Montori, Victor M.

Montorsi, Francesco

Montoto, Silvia

Montuenga, Luis

Monzen, Satoru

Moodley, Jennifer

Moodley, Shaylan

Moon, Dae Hyuk

Moon, Dominic H.

Moon, In Seok

Moon, Jong Ho

Moon, Kyung Chul

Moons, Karel G. M.

Moore, Assaf

Moore, Elizabeth

Moore, Steven C.

Moorman, Anthony V.

Moorthy, Srikant

Mor, Gil

Morabito, Alessandro

Morain, Stephanie

Morales, Rafael

Moran, Brendan

Morana, Giovanni

Morandi, Luca

Morariu, Elena

Moreno, Lucas

Moreno‐Indias, Isabel

Moreno‐Jiménez, Sergio

Moretti, Ugo

Morgan, David

Morgan, Gareth J.

Morgan, Paul

Morgans, Alicia

Morganstern, Dan

Morgenstern, Daniel

Mori, Keiichiro

Mori, Masaki

Mori, Seiichi

Mori, Taisuke

Moriggl, Richard

Moriguchi, Michihisa

Morimoto, Hiroaki

Morimoto, N.

Morin, Ryan

Moris, Lisa

Morishita, Asahiro

Morita, Tatsuya

Morizane, C.

Moroni, R. M.

Morosi, Carlo

Moro‐Sibilot, Denis

Morris, Alan

Morris, Don

Morris, James

Morris, John

Morris, Kevin V.

Morris, Luc G.T

Morrison, Jo

Morrissette, Jennifer J. D.

Morschhauser, Franck

Morse, Elliot

Morse, Lee

Morse, Michael

Mortezaee, Keywan

Morton, Gerard

Morton, Lindsay

Morton, Rachael L.

Moscat, Jorge

Moser, Justin

Mosquera, Juan Miguel

Moss, Haley

Moss, Jennifer

Mossman, Karen L.

Moss‐Morris, Rona

Mota, Jose Mauricio

Motoyama, Satoru

Mott, David

Mott, Louisa

Motwani, Shveta

Motzer, Robert

Mougalian, Sarah

Moul, Judd

Mousa, Shaker

Mousli, A.

Mowla, Seyed Javad

Moyers, Justin

Moynihan, Meghan

Moyses, Raquel

Moysich, Kirsten

Mravec, Boris

Mroz, Thomas

Mrozek, Krzysztof

Msaouel, Pavlos

Mu, Lan

Mucci, Lorelei

Muckaden, Mary Ann

Mücke, Martin

Mudduluru, Giridhar

Mueller, Rolf

Muendlein, Axel

Muffly, Lori

Mugavero, M. J.

Mughal, Amreen

Muhsen, Ibrahim

Mukherjee, Priyabrata

Mukohara, Toru

Mulcahy, Mary

Mulder, Fritz

Mulhall, John

Mulholland, Paul

Muller, David C.

Müller, Volkmar

Mulligan, Stephen

Mullighan, Charles

Mulrooney, Daniel

Multhoff, Gabriele

Mun, Mingyon

Mundell, Niamh

Munene, Gitonga

Munoz‐Erazo, Luis

Munsell, Mark

Munshi, Hidayatullah G.

Munzone, Elisabetta

Murad, M.

Murakami, Hirokazu

Murakami, Naoya

Murakawa, Yasuhiro

Murata, Michio

Murata, Shinichi

Murata, Shin‐ichi

Murhekar, Kanchan

Murphy, Barbara

Murphy, Adam

Murphy, Declan G.

Murphy, James

Murphy, Neil

Murray, Christopher

Murray, Christopher J. L.

Murray, Nigel

Murtagh, Fliss E. M.

Murthy, Prithvi B.

Murtola, Teemu J.

Murty, Vundavalli

Muscarella, Peter

Musher, Ben

Mussetti, Alberto

Mustian, Karen

Mustjoki, Satu

Muthiah, Arun

Mutter, Robert

Muzaffar, Jameel

Muzii, Ludovico

Mydin, Rabiatul Basria S. M. N.

Myers, J.

Myklebust, Mette Pernille

Myrehaug, Sten

Na, Dong Gyu

Na, Hee Young

Na, Rong

Na‐Bangchang, Kesara

Nabavizadeh, Behnam

Nabeshima, Kazuki

Nabhan, Fadi

Nabors, Burt

Naci, Huseyin

Nadella, Sandeep

Nadkarni, Girish N.

Nadler, Eric

Nag, Shona

Nagahara, Akihito

Nagai, Hirokazu

Nagai, Kazuki

Nagakawa, Yuichi

Nagane, Motoo

Nagano, Hideki

Nagao, Shoji

Nagar, Himanshu

Nagarajan, Priyadharsini

Nagasaka, Takeshi

Nagashima, Fumio

Nagata, Chisato

Naghavi, Arash

Naghavi, Mohsen

Nagino, Masato

Nagle, Christina

Nagoor, Noor Hasima

Nagore, Eduardo

Nagorney, David M.

Nagrath, Sunitha

Nagy, Ádám

Nahata, Leena

Naik, Aanand D.

Naing, Aung

Nair, Reena

Nair, Velu

Naito, Sei

Naito, Yoichi

Naito, Yoshiki

Naito, Yuji

Najima, Yuho

Nakagawa, Hidetoshi

Nakagawa, Kazuhiko

Nakagawa, Masao

Nakagawa, Shigeki

Nakagawa, Tohru

Nakagawara, Akira

Nakahama, Kenji

Nakahara, Yoshiro

Nakai, Ryuichiro

Nakajima, Takahiro

Nakajima, Takahito

Nakalembe, Miriam

Nakamoto, Yuji

Nakamura, Haruhiko

Nakamura, Masahiko

Nakamura, Naoya

Nakamura, Nobuhiko

Nakamura, Shigeo

Nakamura, Yusuke

Nakanishi, Yoshito

Nakano, Masahito

Nakaoka, Shinji

Nakashima, Kazuhisa

Nakatani, Fumihiko

Nakayama, Goro

Nakayama, Haruhiko

Nakayama, Takeo

Nakayama, Yujiro

Nakazawa, Atsuko

Nam, Chung Mo

Nam, Do‐Hyun

Nam, Eun Ji

Nam, Joo Hyun

Nam, Kwangwoo

Nam, Robert

Nam, Seok Jin

Nam, Seungyoon

Namazov, Ahmet

Nanji, Sulaiman

Naoki, Katsuhiko

Napel, Sandy

Napoli, Alessandro

Naraev, Boris

Narain, Kanwar

Naranbhai, V.

Narasimhaiah, Deepti

Narayanasamy, Ganesh

Narazaki, Taisuke

Narla, Goutham

Naspro, R.

Nassar, Aziza

Nathan, Hari

Nathan, Paul

Natkunam, Yasodha

Naudin, Sabine

Navaneethabalakrishnan, Shobana

Navari, Rudolph M.

Navi, Babak

Nayar, Ritu

Nazzani, Sebastiano

Nduom, Edjah K.

Neale, Rachel

Neale, Rachel E.

Neamati, Nouri

Necchi, Andrea

Nedjadi, Taoufik

Nedrow, Jessie

Neesse, Albrecht

Negri, Eva

Negrier, Sylvie

Neilan, Tomas

Nelson, Blessie Elizabeth

Nelson, Cathrine

Nelson, Rebecca

Nephew, Ken

Nephew, Kenneth P.

Nephew, Lauren

Neri, Luca M.

Ness, Kirsten K.

Neuman, Heather

Neupane, Prakash

Neupane, Subas

Neuwelt, Edward

Neuzillet, Cindy

Neuzillet, Y.

Nevanlinna, Heli

Newcomb, Polly

Newton, Robert

Newton‐Bishop, Julia

Neyns, Bart

Nezami, Behtash

Ng, Chi‐Fai

NG, Ho‐Keung

Ng, Lui

Ng, Siew Chien

Ng, Simon SM

Ng, Siok Bian

Ng, Sweet Ping

Ng, Xinyi

Nghiem, Paul

Ngiam, Kee Yuan

Ngo, Thuy Trang

Nguyen, Anvy

Nguyen, Paul

Nguyen, Toan

Ni, Caifang

Ni, Jiali

Ni, Liwei

Ni, Shujuan

Ni, Zhong

Nichols, Hazel

Nichols, Kim

Nickerson, Michael

Nickols, Nicholas

Nicolai, Nicola

Nicolazzo, Chiara

Nie, Mingming

Nie, Yongzhan

Niedzielski, Joshua S.

Niedzwiecki, Donna

Nielsen, Torsten

Niemann, Carsten

Niesvizky, Ruben

Nieto, Maria

Niikura, Hitoshi

Niikura, Naoki

Niinivirta, Marjut

Niino, Daisuke

Nikiforov, Yuri E.

Nikolaou, Christoforos

Nikpour, Parvaneh

Nilsen, Marci L.

Nilsson, Lena

Nilsson, P. J.

Nimitrungtawee, Natthaphong

Nimptsch, Katharina

Nindrea, Ricvan Dana

Ning, Gang

Ning, Guang

Ning, XIao

Nio, Koki

Nipp, Ryan

Niraula, Saroj

Nishibuchi, Ikuno

Nishida, Toshirou

Nishihori, Taiga

Nishikawa, Hiroyoshi

Nishikawa, Shinpei

Nishimura, Masaharu

Nishino, Ryuhei

Nishio, Kazuto

Nishio, Shin

Nishizawa, Yuji

Niu, Junqi

Niu, Ningning

Niu, Tianye

Niu, Wenquan

Niu, Xiaomin

Niu, Xinhao

Niu, Yang

Niu, Yu‐Na

Noale, Marianna

Noble, Mark

Nobuo, Suzuki

Nock, Matthew

Nock, Nora

Nodeh, Mohammad

Noepel‐Duennebacke, Stefanie

Noguchi, Rei

Noguchi, Takafumi

Noh, Dong‐Young

Noh, Joon Hwa

Nohl, Sung Hoon

Nong, Sikai

Nooka, Ajay

Norat, Teresa

Nordstrom, Tobias

Normanno, Nicola

Noronha, Vanita

Nortje, Nico

Nosho, Katsuhiko

Nossiter, Julie

Nourbakhsh, Mitra

Noushmehr, Houtan

Novara, Giacomo

Novello, Silvia

Nowak, Kai

Nowak, Rebecca G.

Nowakowski, Francis S.

Ntekim, Atara

Nugent, Shannon

Nuhn, Philipp

Numakura, Kazuyuki

Nunez, Carlos

Nussenbaum, Brian

Nuti, Marianna

Nuttall, Elisabeth

Nuyts, Sandra

Nyante, Sarah

Nygard, Mari

Nyman, Ulf

Nyqvist, Jenny

Nyrop, Kirsten

O'Regan, Kevin

Oakley‐Girvan, Ingrid

Oancea, Elena

Obama, Kazutaka

Obeng‐Gyasi, Samilia

Oberai, A.

Oboite, Joan

O'Brien, Susan

Ocampo, Miguel Michel

Ochiai, Atsushi

Ocio, Enrique

O'Connell, Ryan

Odejide, Oreofe

Odermatt, Alex

Odo, Daniel Bogale

O'Dorisio, M Sue

Oeffinger, Kevin

Oettle, Helmut

Offit, Kenneth

Offner, Fritz

Ofuji, Kazuya

Ogasawara, Sadahisa

Ogata, Dai

Ogata, Kei

Ogata, Suguru

Ogata, Takashi

Ogawa, Mikako

Ogawa, Seishi

Ogbo, Felix Akpojene

Ogilvie, Gina

Ogino, Atsuko

Ogino, Shuji

Oguma, Junya

Ogunwobi, Olorunseun

Ogura, Michinori

Oh, Byeongsang

Oh, Chang‐Mo

Oh, Do‐Youn

Oh, Heung‐Kwon

Oh, Hyun‐Mee

Oh, Jae Hwan

Oh, Jong Jin

Oh, Pok Ja

Oh, Pok‐Ja

Oh, Sung Yong

Ohama, Hideko

Ohanian, Maro

Ohashi, Kadoaki

Ohashi, Yasuo

Ohgami, Robert

Ohmachi, Ken

Ohmori, Tohru

Ohneberg, Kristin

Ohri, Nitin

Ohshima, Kengo

Ohuchi, Noriaki

Oing, Christopher

Ojerholm, Eric

Ojha, Rohit P.

Okabayashi, Koji

Okada, Keiko

Okada, M.

Okada, Morihito

Okafuji, Hirofumi

Okami, Kenji

Okamoto, Haruko

Okamoto, Hiroshi

Okamoto, Takeshi

Okamoto, Tatsuro

Okamura, Hitoshi

Okano, Naohiro

Okano, Shinji

Okato, Atsushi

Okayama, Hirokazu

Oki, Eiji

Oki, Yasuhiro

Okimoto, Kenichiro

Okodo, Mitsuaki

Okolie, Elvis

Okonogi, Noriyuki

Okuda, Shigeo

Okugawa, Kaoru

Okugawa, Yoshinaga

Okuyama, Hiroyuki

Okuyama, Toru

Olawaiye, Alexander B.

Oldan, Jorge

Olesen, Jonas Bjerring

Oliveira, Sergio C.

Oliveira, Carla

Oliveira, Junea

Oliveira, Paula Alexandra

Oliver, Francisco

Oliver, Antony W.

Olivotto, Ivo

Olofsson‐bagge, Roger

Olsen, Catherine

Olshan, Andrew

Olson, Kristine

Olsson, Maria

Olszewski, Adam

Olver, Ian

Omae, Kenji

O'Mara, Tracy

Omarini, Claudia

Omede, Paola

Omofuma, Omonefe

Omran, M.

omran, mohamed

Omuro, Antonio

Onal, Cem

Onate‐Ocana, Luis F.

Ondrey, Frank

O'Neal, Richard

Onega, Tracey

O'Neill, Kim

O'Neill, Luke A. J.

Onesti, Concetta Elisa

Ong, Choon Kiat

Ong, Wee Loon

Ono, Akira

Ono, Kana

Ono, Masayoshi

Ono, Yuko

Onukwugha, Eberechukwu

Oo, Thein

Ooba, Akihiro

Oppong, Bridget

Orbach, Daniel

Ording, Anne

Ordu, Cetin

O'Reilly, Eileen

O'Reilly, Séamus

Oren, Ohad

Orfao, Alberto

Orgel, Etan

Orimo, Akira

Orio, Peter F.

Oriol, Mathieu

Orlowski, Robert

Orsey, Andrea

Orsulic, Sandra

Ortega, Cinzia

Ortiz, Ana

Osada, Hiroyuki

Osazuwa‐Peters, Nosayaba

Oscorbin, Igor

Oshima, Takashi

Osman, Iman

Osman, Mona

Osman, Yasser

Ost, Piet

Ostergaard, Anna

Ostoros, Gyula

Ostroff, Jamie

Ostrowska, Beata

Ostrowski, Jerzy

Ostuzzi, Giovanni

Ostwal, Vikas

Osumi, Hiroki

Ota, Ichiro

Ota, Takayo

Otero, Hansel J.

Othman, Ahmed

Othman, Ahmed E.

Otsuji, Eigo

Otsuka, Atsushi

Otsuka, Motoyuki

Ott, Patrick A.

Otten, Hans‐Martin

Ottensmeier, Christian H.

Otterson, Greg

Otto, Mario

Ou, Sai‐Hong Ignatius

Oudkerk, Matthijs

Ouyang, Jun

Ouyang, Peilin

Øvlisen, Andreas

Ow, Andrew

Owattanapanich, Weerapat

Owen, Carolyn

Owens, Douglas K.

Oxnard, Geoffrey R.

Oya, Mototsugu

Oza, Amit

Ozaki, Akihiko

Ozaki, Iwata

Ozanne, Elissa

Ozawa, Tsuyoshi

Ozer, Byram

Özgüzer, Alp

Ozpolat, Bulent

Ozturk, Erhan Arif

Ozturk, Ma

Pace, Leonardo

Pace, Nathan Leon

Pace, Thaddeus W. W.

Pacey, Simon

Pacheco‐Barcia, Vilma

Pachynski, Russell

Padayachee, Eden

Pagano, Livio

Page, Alexander

Page, Ray

Pai, Reetesh K.

Paice, Judith

Paik, Soonmyung

Pajic, Marina

Pajtler, Kristian

Pak, Kyoungjune

Pakpour, Amir H.

Pal, Sumanta

Pal, Tuya

Palacios‐Cena, Domingo

Palaniappan, Latha

Palanisamy, Viswanathan

Palassini, Elena

Palazzo, Juan

Palesh, Oxana

Paley, Carole

Palicharla, Vivek

Palin, Kimmo

Palma, Giuseppe

Palmer, Joshua

Palmer, Nynikka R.

Palmer, Steven

Palmerini, Emanuela

Palmieri, Giuseppe

Palmirotta, Raffaele

Palumbo, Antonio

Pan, Guangdong

Pan, Guangrui

Pan, Hao

Pan, Hen‐Chih

Pan, Jianwei

Pan, Jing‐Hua

Pan, Jingxuan

Pan, Qiufeng

Pan, Qiuzhong

Pan, Shaokang

Pan, Shiyang

Pan, Wensheng

Pan, Wen‐Tao

Pan, Xiaofen

Pan, Yi

Pan, Yida

Pan, Yun‐long

Pan, Zhizhong

Pan, Zihao

Pan, Zongfu

Panageas, Katherine

Panchal, Hina

Pande, Mala

Pandi, Narayanan Sathiya

Pandolfi, Pier Paolo

Pang, Da

Pang, Lihong

Pang, Qing

Pang, Qingsong

Pang, Ting

Pang, Xiongwen

Pang, Xiufeng

Pang, Yu‐Yan

Pangas, Stephanie

Pan‐Hammarstrom, Qiang

Paniccia, Alessandro

Pant, Shubham

Pantheon, Nike

Pantuck, Allan

Papachristou, Nikolaos

Papadakos, Janet

Papadimitrakopoulou, Vali

Papadopoulos, Efthymios

Papadopoulos, Kyriakos P.

Papaleo, Elena

Papatheodoridis, George V

Papavassiliou, Athanasios

Papaxoinis, George

Papotti, Mauro

Paradis, Valérie

Parajuli, Parash

Pardini, Barbara

Pare, Peter D.

Parekh, Samir

Parganiha, Arti

Parham, Sendi

Parikh, Purvish

Parikh, Rahul

Paris, Pamela

Park, Hee Chul

Park, Bernard J.

Park, Chul

Park, Do Hyun

Park, Do Joong

Park, Do Youn

Park, Elyse

Park, Hannah

Park, Henry

Park, Hyunjin

Park, In Ah

Park, In Ja

Park, Inkeun

Park, Jae Kil

Park, Jae Young

Park, Jeong‐Yeol

Park, Ji Eun

Park, Jinhee

Park, Jong

Park, Jong Kuk

Park, Jun Chul

Park, Keunchil

Park, Leeza

Park, Peter

Park, Richard Chan Woo

Park, S. Y.

Park, Sang‐Jae

Park, So‐Yeon

Park, Sue K.

Park, Su‐Hyung

Park, Wan Beom

Park, Won

Park, Yikung

Park, Yong

Park, Yong Gyu

Park, Young Hoon

Park, Young Joo

Park‐Min, Kyung‐Hyun

Parmar, Ambica

Parracchio, Letizia

Parrella, Paola

Parsa, Andrew T.

Parsons, Helen

Partap, Sonia

Parvathaneni, Upendra

Paskett, Electra

Pasparakis, Manolis

Pasquali, Sandro

Passamonti, Francesco

Pasternak, Bjorn

Pasticier, Gilles

Pastori, Daniele

Patel, Alpa

Patel, Keval

Patel, Keyur

Patel, Kirtesh

Patel, Manal I.

Patel, Manali I.

Patel, Mihir

Patel, Shilpa

Pathak, Jyotishman

Pathak, Yashwant V.

Pathy, Sushmita

Patierno, Steven

Patil, Dattatraya

Patil, Chandragouda R.

Patil, Chirag G.

Patra, Krushna

Patra, Samir

Pattanapanyasat, Kovit

Patti, Caterina

Paty, Jean

Paty, Philip B.

Paul M., Schneider

Paul, Rahul

Paulos, Chrystal

Paulson, Kelly

Paulsson, Johan

Pause, Arnim

Pavel, Marianne

Pavlick, Anna

Pavlova, Sarka

Pavlovic, Natasa

Pawlik, Timothy

Paydas, Semra

Payne, H.

Payne, Judith

Paz‐Ares, L.

Paz‐Ares, Luis

Pearce, Edward J.

Pearlman, Rachel

Pearson, Alexander

Pecoraro, Angela

Peeters, Marc

Peg, Vicente

Pehalova, Lucie

Pei, Hailong

Pei, Haiping

Pei, Jun‐Peng

Pei, Lin

Peleg Nesher, Sharon

Pellati, Federica

Pellegrina, Diogo

Pellissier, James

Pemmaraju, Naveen

Peng, Changliang

Peng, Cheng

Peng, Guangyong

Peng, Hao

Peng, Jian

Peng, Jianhong

Peng, Jun

Peng, Junjie

Peng, Junsheng

Peng, Kun‐Wei

Peng, Liubao

Peng, Min

Peng, Qing

Peng, Sui

Peng, Tzu Rong

Peng, Wei

Peng, Zhi‐gang

Pennell, Nathan

Penney, Kathryn

Pentheroudakis, George

Perales, Miguel‐Angel

Perduca, Vittorio

Pereira, Deidre

Pereira, Marina Alessandra

Perez, Edith A.

Perez, Giselle K.

Perez, Rodrigo

Perez‐Cornago, Aurora

Perez‐Plasencia, Carlos

Pergam, Steven

Pericay, Carles

Permuth, Jennifer

Perna, Fabiana

Perner, Susanne

Perol, Maurice

Perou, Charles M.

Perrone, Francesco

Perrot, Aurore

Pertwee, Roger

Peters, Anthea

Peters, Katherine

Peters, Mary Linton

Peters, Solange

Peters, Ulrike

Peterson, Susan

Petkovska, Iva

Petralia, Giuseppe

Petreaca, Ruben

Petrick, Jessica

Petrillo, Marco

Petrosino, Joseph

Petrou, Stavros

Pevet, Paul

Peymani, Maryam

Peyrade, Frédéric

Pezzullo, Luciano

Pfeffer, Ulrich

Pfister, Christian

Pfister, S.

Pfluger, Thomas

Pfreundschuh, Michael

Phelan, Rachel

Philip, Arun

Philip, Philip

Philips, Anne

Phillips, David

Phillips, Phoebe

Phillips, Tycel

Phillips, Wayne A.

Philpot, Lindsey

Phipps, Sean

Piao, Daxun

Piao, Haozhe

Piccardo, Arnoldo

Picci, Piero

Piché, Alain

Pichler, Herbert

Pichler, Martin

Pickles, Tom

Picozzi, Vincent

Picozzi, Vincent J.

Pielkenrood, Bart

Piemontese, Simona

Pienta, Kenneth

Pierce Campbell, Christine

Pierce, Brandon

Pierce, Sherry

Pierga, Jean‐Yves

Pierobon, Massimiliano

Piessen, Guillaume

Pieters, Rob

Pietrantonio, Filippo

Pietrowska, Monika

Piggott, Carolyn

Pijnenborg, Johanna

Pilarski, Robert

Pilarsky, Christian

Pillai, Jay J.

Pillai, Radhakrishna

Pillow, Thomas

Pilon, D.

Pimenoff, Ville

Pinato, David James

Pineda, Marta

Pineros, Marion

Ping, Shuai

Ping, Zhiguang

Pinheiro, Joao

Pinheiro, Laura

Pinheiro, Paulo

Pinheiro, Paulo S.

Pinilla‐ibarz, Javier

Pinkawa, Michael

Pinlaor, Somchai

Pintelas, Emmanuel

Pinthus, J. H.

Pintilie, Melania

Pinto, Monica

Piper, Rory

Pira, Enrico

Pires, Bruno R. B.

Pirker, Robert

Piromchai, Patorn

Pirtoli, Luigi

Pisapia, Pasquale

Piscaglia, Fabio

Pischon, Tobias

Piscuoglio, Salvatore

Pisu, Maria

Pitoia, Fabian

Pixley, Fiona J.

Pizzato, margherita

Plass, Christoph

Plastaras, John

Platz, Elizabeth A.

Plesner, Torben

Plichta, Jennifer K.

Ploussard, Guillaume

Poddar, Soumya

Poggi, Alessandro

Poiret, Thomas

Polanco, Patricio

Polesel, Jerry

Politi, Mary

Poljak, Mario

Pollak, Kathryn

Pollak, Michael

Poller, David

Pollock, Brad

Pollock, Philip

Poluzzi, Elisabetta

Pombo‐de‐Oliveira, Maria S.

Pompe, Raisa

Pongsavee, Malinee

Ponte, Jose

Poole, Beth

Poon, Darren Ming‐Chun

Poon, DM

Poon, Michelle LM

Poort, Hanneke

Pope, Whitney

Poplawski, Tomasz

Popper, Helmut

Porcel, Jose M.

Porpiglia, Francesco

Port, Jeffrey

Porta, Camillo

Portell, Craig

Porter, James R.

Portillo, Isabel

Portugal, Liana C.L

Posner, Marshall

Poston, Graeme J.

Postow, Mike

Potenza, Leonardo

Pothuri, Bhavana

Pottel, Hans

Potter, John

Pöttgen, Christoph

Poulsen, Mads

Poultsides, George A.

Pour, Ludek

Pourahmad, Jalal

Pourshams, Akram

Powe, D.G

Powell, Arfon

Powell, Charles

Power, Chris

Prabash, Kumar

Prabhash, Kumar

Prada, Carlo

Prada, Diddier

Prada, Francesco

Pradere, Benjamin

Prado, Carla

Prasad, Birbal

Prats‐Uribe, Albert

Prayongrat, Anussara

Prebet, Thomas

Preda, Veronica

Preiksaitis, Jutta

Preisser, Felix

Press, Michael

Press, Robert

Prestwich, Robin

Prete, Francesco

Preudhomme, Claude

Preusser, Matthias

Prigerson, Holly

Prins, Martin

Pritchard, Antonia

Pritchard, Colin C.

Prizment, Anna

Prochazka, Vit

Prochownik, Edward V.

Procopio, Giuseppe

Proietti, Marco

Prologo, J. David

Prosperi, Jenifer R.

Protani, Melinda

Provero, Paolo

Psutka, Sarah

Psyrri, Amanda

Pu, Yeong‐Shiau

Pu, Zening

Pucciarelli, Gianluca

Pugalendhi, Pachaiappan

Puglisi, Fabio

Puglisi, Lisa

Pui, Ching‐Hon

Puig, Susana

Pujol, Miquel

Pukkala, Eero

Punglia, Rinaa

Punnett, Angela

Punyadeera, Chamindie

Puram, Sidharth

Puri, Varun

Purohit, Rituraj

Puspitasari, Irma

Pusztai, Lajos

Putignani, Lorenza

Putter, Hein

Puvanesarajah, Samantha

Puzanov, Igor

Qi, Feng

Qi, Hongbo

Qi, Lu‐Nan

Qi, Mingran

Qi, Ruiqun

Qi, Shaohai

Qi, Shouliang

Qi, Xiaolong

Qi, Xiaowei

Qian, Chao‐nan (Miles)

Qian, Da

Qian, Danwen

Qian, Feng

Qian, Guo‐Feng

Qian, Kaiyu

Qian, Xiaojun

Qian, Yaowen

Qian, Zenghui

Qiang, Guangliang

Qiang, Jinwei

Qiao, Bin

Qiao, Jie

Qiao, Qiao

Qiao, Youlin

Qin, Changjiang

Qin, Chao

Qin, Hongyan

Qin, Jianmin

Qin, Na

Qin, Shukui

Qin, Wenxin

Qin, Xue

Qin, Yanmei

Qin, Ya‐Zhen

Qin, Yigying

Qingxi, Yue

Qiu, Jiangdong

Qiu, Lihua

Qiu, Mantang

Qiu, Meng

Qiu, Qingtao

Qiu, Shuang‐Jian

Qiu, Su

Qiu, Sufang

Qiu, Wu

Qiu, Xiao‐Qiang

Qiu, Yuanzheng

Qiu, Yudong

Qiu, Zeting

Qiu, Zhixin

Qu, Hong

Qu, Jinrong

Qu, Kai.

Qu, Pengpeng

Qu, Xiaofei

Qu, Xiujuan

Qu, Ying

Qu, Yunhui

Quackels, Thierry

Quan, Jingzi

Quan, M. L.

Quanlin, Li

Quattrocchi, Carlo Cosimo

Queiroga, Felisbina

Querec, Troy

Quick‐Weller, Johanna

Quinn, Gwendolyn

Quinn, Julian M. W.

Quinn, Virginia P.

Quint, Wim G. V.

Quinten, Chantal

Quirke, Philip

Quiroga‐Centeno, Andrea Carolina

Quon, Harry

R, Cecilie Buskbjerg

R. I., Anu

R. O'Donnell, Ronald

R.M, Hermann

RÃ¸der, M. Andreas

Raab, Marc‐Steffen

Raben, David

Raby, Benjamin

Radaelli, Franco

Raderer, Markus

Radhakrishnan, Sabarinathan

Radke, Josefine

Radocha, Jakub

Raftery, Daniel

Raggi, Chiara

Raggi, Daniele

Raghav, Kanwal

Raghava, Gajendra P. S.

Ragragui Ouasmin, Hanan

Ragusa, Marco

Rahib, Lola

Rahman, Momotazur

Rahman, Rakhshanda Layeequr

Rahouma, Mohamed

Raine, Rosalind

Raj, Minakshi

Raj, Rishi

Rajagopalan, Anugraha

Rajan, Suja S.

Rajan, Surulivel

Rajaraman, Swaminathan

Rajendran, Venkadesan

Rajeswaran, R.

Rajkumar, S. V.

Rajkumar, V.

Rajpert‐De Meyts, Ewa

Rakha, Emad

Rakhsha, Afshin

Raldow, Ann

Ramachandran, Vasan

Ramaiah, M. Janaki

Ramakrishnan, Seshadri

Ramakrishnan, Swathi

Ramalho, Susana

Ramanathan, Ramesh K.

Ramaswamy, Bharath

Ramaswamy, Bhuvaneswari

Rambaldi, Alessandro

Ramirez de Molina, Ana

Ramirez, Amelie

Ramirez, Pedro

Ramirez, Robert A.

Ramirez‐Moya, Julia

Ramjeesingh, Ravi

Rammstedt, Beatrice

Ramondetta, Lois

Ramos, Jorge

Ramos, Jorge D.

Ramos, Rommel T. J.

Ramsenthaler, Christina

Ramsey, Scott

Ramshankar, Vijayalakshmi

Ran, Mao‐Sheng

Randhaw, Gurvaneet

Randi, Giorgia

Ranft, Andreas

Ranga, Upasana

Ranganathan, Balu

Ranganathan, Prathibha

Rangarajan, Venkatesh

Rankin, Nicole

Rankin, Nicole M.

Rao, Chinthalapally

Rao, Devika

Rao, Mahadev

Rao, Qunxian

Rao, Sheng‐Xiang

Raphael, Jacques

Rapoport, Bernardo

Rapoport, Bernardo L.

Rashed, Wafaa

Raspollini, Maria Rosaria

Rathod, Sanjay

Rau, Rachel

Rauscher, Garth

Raut, Chandrajit P.

Ravandi, Farhad

Ravaud, A.

Ravindran, Reshmi

Ravlić, Sanda

Ray‐Coquard, Isabelle

Rayne, Sarah

Raz, Abraham

Raz, Dan J.

Razak, Albiruni

Razak, Siti Razila Abdul

Razanamihaja, Noeline

Rea, Delphine

Ready, Neal

Rebers, Susanne

Reddy, Suresh

Reding, Kerryn

Redmer, Torben

Redmond, Kristin J.

Reed, Damon

Reeder‐Hayes, Katherine

Rees, PA

Reesink, Daan J.

Rees‐Punia, Erika

Reeve, Bryce

Reeves, Gillian K.

Reguart, Noemi

Reiberger, Thomas

Reichardt, Peter

Reid, Michelle

Reijnen, Casper

Reim, Daniel

Reimand, Jüri

Reinacher, Peter C.

Reiner, Anne

Reinhardt, Dirk

Reis, Patricia Pintor dos

Reis, Rui

Reis, Rui Manuel

Reiter, Andreas

Reiter, RJ

Remon, Jordi

Ren, Shancheng

Ren, Dong

Ren, Fang

Ren, Fangmei

Ren, Guoxin

Ren, Hanyun

Ren, Haoyu

Ren, Kang

Ren, Li

Ren, Min

Ren, Mingyu

Ren, Ning

Ren, Shengxiang

Ren, Shuai

Ren, Xiaohan

Ren, Xiaohui

Ren, Xuehan

Ren, Yijiu

Ren, Zefang

Ren, Zhenggang

Ren, Zheng‐Gang

Rengan, Ramesh

Reni, Michele

Renieri, Alessandra

Rentscher, Kelly

Repasky, Elizabeth

Resch, I.

Resende, Uanderson

Reshetniak, Oksana

Reulen, Raoul

Reuter‐Lorenz, Patricia A.

Reyes, Ryan

Reynolds, Kerry

Rezaee, Seyed Abdolrahim

Rezaianzadeh, Abbas

Rezende, Leandro F. M.

Rha, Koon Ho

Ri, Masaki

Riascos, Roy Francisco

Ribas, Antoni

Ribeiro, Karina

Ribeiro, Raul

Ribero, Simone

Riboli, Elio

Ricciuti, Biagio

Rice, Mikhaila

Ricevuto, Enrico

Richards, Kyle

Richardson, Lisa

Richardson, Paul

Richiardi, Lorenzo

Richter, Kimber P.

Ricks‐Santi, Luisel

Riddell, Stanley R.

Riechelmann, Rachel

Riera‐Knorreschild, Jordi

Riesco‐Eizaguirre, Garcilaso

Riethdorf, Sabine

Riffaud, Laurent

Righi, Alberto

Rigotti, Nancy

Riker, Adam

Rimassa, Lorenza

Rimm, Eric B.

Rinaldi, Sabina

Ring, Kari

Ringuette Goulet, Cassandra

Rini, Brian

Rini, Brian I.

Rink, M.

Rinke, Anja

Riquelme, Ismael

Ris, M. Douglas

Risch, Harvey

Ritch, Chad

Rittscher, J.

Rivas, Carol

Rizki, Humna

Rizvi, Naiyer A.

Rizvi, Sumera

Rizzari, Carmelo

Rizzo, Davide

Rizzo, Francesca

Ro, Jae Y.

Roa, Juan Carlos

Robar, James L.

Robbins, Anthony

Robbins, Jared

Roberson, Mya

Robert Cormier, Robert Cormier

Roberto, Michela

Roberts, Andrew

Roberts, David D.

Roberts, Neil

Robertson, John

Robertson, Keith

Robesti, Daniele

Robetorye, Ryan

Robien, Kim

Robijns, Jolien

Robinson, Andrew

Robinson, Clifford

Robinson, David

Robinson, David L.

Robinson, Whitney

Robison, Katina

Robison, Leslie L.

Robles, Ana I.

Robles, Joanna

Robson, Craig

Rocha, Flavio

Rochigneux, Philippe

Röcken, Christoph

Rockwood, Kenneth

Rocque, Gabrielle

Rodgers, Jessica R.

Rodin, Danielle

Rodríguez, Amaia

Rodriguez, Analiz

Rodriguez, Elizabeth

Rodriguez, Luis A Garcia

Rodriguez‐Aguayo, Cristian

Rodríguez‐Alfonso, B.

Rodriguez‐Galindo, Carlos

Rodríguez‐Lobato, Luis‐Gerardo

Roeder, F.

Roeder, Falk

Roesch, Alexander

Roessler, Stephanie

Roger, Veronique L.

Rogers, Craig G.

Rogers, Hazel

Rogers, Kerry

Rogers, Laura Q.

Roh, Jong‐Lyel

Rohan, Thomas

Rohr‐Udilova, Nataliya

Roikjaer, O.

Rojas, Ana

Rolfo, Christian

Rollo, Francesca

Roman, Eve

Romano, Alessandra

Romao, Luisa

Romejko‐Jarosinska, Joanna

Romer, Georg

Romero Béjar, Jose Luis

Romero, Sally

Romesser, Paul

Romieu, Isabelle

Ronckers, Cecile M.

Rondot Radío, Pedro José Carlos

Ronellenfitsch, Ulrich

Rong, Lijun

Rong, Zhili

Roos, Goran

Rosa, Fausto

Rosa, Marilin

Rosa, Nicola

Rosado, Juan

Rosario, Pedro Weslley

Rosati, Gerardo

Rosato, Antonio

Roscoe, Joseph A.

Rose, Melanie

Rose, Peter G.

Rosell, Rafael

Rosen, Neal

Rosenberg, Aaron

Rosenberg, Abby

Rosenberg, Ari

Rosenberg, Jonathan

Rosenberg, Steven A.

Rosenfeld, Michael G.

Roser, Katharina

Rosiello, Giuseppe

Rosillon, Dominique

Ross, Joseph S.

Ross, Paul

Rossell, Nuria

Rossi, Antonio

Rossi, Esther Diana

Rossi, Giovanni

Rossi, Sabrina

Rostila, Mikael

Rostomily, Robert

Roszik, Jason

Rota, M.

Rotblat, Barak

Roth, Gregory A.

Roth, Michael

Rotolo, Nicola

Rotte, Anand

Rotter, Varda

Rottey, Sylvie

Roukos, Dimitrios

Roumeguere, T.

Rousseau, Benoit

Rousseaux, Sophie

Routbort, Mark

Roxburgh, Campbell

Roy, Hemant

Roy, Pascal

Roychoudhury, Susanta

Royle, Gary

Rozen, Steven George

Ru, Heng

Ru, Huang

Ru, Kun

Ruan, Jian

Ruan, Jichen

Ruan, Jingjing

Ruan, Min

Ruan, Shanming

Ruane‐McAteer, Eimear

Rubens, Muni

Rubin, Brian

Rubin, Stephen

Rubinson, Douglas

Rubio, Isabel T.

Rubio, Marie Thérèse

Rudd, Kristina E.

Rudin, Charles

Rudzinski, Erin

Ruecker, Frank

Rueegg, Corina S.

Ruff, Paul

Ruffin, Mack

Rugeles, Maria T.

Rugo, Hope

Rui, Ma

Rui‐lan, Wang

Ruiz, Emily S.

Rumman, Marufa

Rundek, Tatjana

Rundle, Andrew

Rush, Nancy

Russi, Elvio G.

Russo, Annapina

Russo, Antonio

Russo, Antonio Giampiero

Russo, Ethan B.

Ruszniewski, Philippe

Rutka, James T.

Rutter, Carolyn M.

Ruzgar, Nensi Melissa

Ruzzene, Maria

Ruzzo, Annamaria

Ryan, Aoife

Ryan, Casey

Ryan, Elizabeth

Rybicki, Benjamin

Rydén, Isabelle

Rydzewska, Larysa

Rye, Morten

Rymkiewicz, Grzegorz

Ryoo, Baek‐Yeol

Rytting, Michael

Ryu, Ji Kon

Ryu, Yon Ju

Sa, Mousa

Sa, Jason

Saarela, Olli

Saarto, Tiina

Saba, Nabil

Sabari, Joshua

Sabatino, Susan A.

Sabik, Lindsay M.

Sabol, Maja

Saburi, Ehsan

Sacco, Assuntina

Sacco, Rodolfo

Sacher, Adrian

Sadar, Marianne D.

Sadashivadu, Gundeti

Sadeghi‐Naini, Ali

Sadelain, Michel

Sadik, Ahmed

Sadilkova, Lenka

Sadowski, Elizabeth

Saeki, Hiroshi

Saeki, Issei

Saffari, Mohsen

Sagar D Sardesai, Sagar

Saglio, Giuseppe

Saha, Biswarup

Saha, Subbroto

Saha, Subbroto Kumar

Sahebghadam Lotfi, Abbas

Sahgal, Arjun

Sahin, Ibrahim

Sahin, Kazim

Sahoo, Debashis

Sahoo, Ranjit

Saieg, Mauro

Saigusa, Susumu

Saikia, Tapan

Saito, Hiroaki

Saito, Ryuta

Saiyang, Li

Saka, Hideo

Sakaeda, Toshiyuki

Sakai, Ken

Sakakibara, Ayako

Sakamoto, Naoko

Sakhuja, Swati

Sakurai, Hideyuki

Sakwe, Amos M.

Sala, Maria

Saladzinskas, Zilvinas

Salah, Samer

Salama, April

Salama, Joseph K.

Salami, Siamak

Salami, Simpa

Salas, Eduardo

Salati, Massimiliano

Salcedo, Jonathan

Salcher, Maxmillian

Saleem, Imran

Saleh, O.

Salehi‐Abargouei, Amin

Salehi‐Mazandarani, Sadra

Salem, Aliasger

Salem, Hanin

Salerno, Elizabeth

Saliby, Renee Maria

Salinaro, Julia

Salles, Gilles

Salmasi, Amirali

Salo, Jonathan

Salo, Tuula

Salomon, Joshua A.

Saltz, Leonard

Salvatore, Lisa

Salvi, Samanta

Salvia, Roberto

Sam, Mohammad Reza

Samadani, Ali Akbar

Samadani, Uzma

Samawi, Haider

Sameni, Mansoureh

Samoni, Sara

Sampath, Sagus

Sampetrean, Oltea

Sampson, John

Samuel, Cleo

Samuels, Brian

Samuels, Courtney

Samur, Mehmet Kemal

Samy, A. M.

Sanchez‐Salas, Rafael E.

Sancho, Juan Manuel

Sandecka, Viera

Sander, Birgitta

Sanderson, Eleanor

Sandhu, Shahneen

Sandler, Dale

Sandler, Robert S.

Sandoval, José Luis

Sanfilippo, Kristen

Sanft, Tara

Sang, Xinting

Sang, Xin‐Ting

Sangsin, Apiruk

Sang‐Youel, Park

Sankaranarayanan, Rengaswamy

Sanli, Oner

Sannigrahi, Malay

Sano, Daisuke

Sanoguera‐Miralles, Lara

Sansonetti, Philippe J.

Santa Mina, Daniel

Santa‐Maria, Cesar Augusto

Santana, Victor

Santangelo, Gabriella

Santisteban, Pilar

Santoro, Angela

Santos‐Iglesias, Pablo

Saponara, Maristella

Saraceni, Francesco

Saraiya, Mona

Sarfaty, Michal

Sarian, Luis

Sarin, Rajiv

Sarkar, Chandrani

Sarkozy, Clementine

Sarkozy, Clémentine

Sartini, Davide

Sartor, Oliver

Sartore‐Bianchi, Andrea

Sasada, Tetsuro

Sasagawa, Satoru

Sasagawa, Toshiyuki

Sasaki, Koji

Sasaki, Mitsuhito

Sasaki, Takaaki

Sasaki, Takashi

Sasaki, Yasushi

Sassaki, Ligia Yukie

Sastre, J.

Sata, Masafumi

Satake, Hironaga

Satchi‐Fainaro, Ronit

Sato, Hideyo

Sato, Kazuhito

Sato, Keiko

Sato, Mariko

Sato, Yuichi

Sato, Yusuke

Satoh, Taroh

Satoh, Toyomi

Satou, Akira

Satouchi, Miyako

Saussele, Susanne

Sauvageau, Guy

Sauvageau, Martin

Sauvaget, Catherine

Savage, Sharon A.

Savai, Rajkumar

Savarino, Edoardo

Savas, Laura

Savas, Sevtap

Savona, Michael

Sawabe, Michi

Sawada, Akihisa

Sawaki, Masataka

Sawicki, Gregory S.

Saydam, Guray

Sayer, Elinore

Saykin, Andrew

Sboner, Andrea

Scalise, Carly

Scaria, Vinod

Scarpa, Aldo

Scarpa, Marco

Scarpa, Melania

Scarpi, Emanuela

Scarsbrook, Andrew

Scartozzi, Mario

Scelo, Ghislaine

Schaapveld, Michael

Schadendorf, Dirk

Schaefer, Niklaus

Schafer, R.

Schagen, Sanne

Schalken, Jack

Schapira, Lidia

Scheel, Andreas H.

Scheffler, Matthias

Schein, Jeff

Scheithauer, Werner

Schenker, Yael

Scherer, Dominique

Scherer‐Rath, Michael

Schetelig, Johannes

Schiffman, Mark

Schiller, John

Schiller, Killian

Schilling, Bastian

Schittenhelm, Jens

Schlaak, Max

Schlaepfer, IR

Schlecht, Nicolas

Schleiermacher, Gudrun

Schlesinger, Sabrina

Schlom, Jeffrey

Schmid, Marianne

Schmid, Maximilian

Schmidinger, Manuela

Schmidt, Andrew

Schmidt, Brian L.

Schmidt, C. Max

Schmidt, Carl R.

Schmidt, Daniel

Schmidt, Henrik

Schmidt, Marjanka K.

Schmidt, Susanne

Schmidt, Thomas

Schmidt‐Wolf, Ingo

Schmidt‐Wolf, Ingo GH

Schmit, Stephanie

Schmitt, Fernando

Schmitz, Norbert

Schmutzler, Rita

Schneeweiss, Sebastian

Schneider, Brian

Schneider, Jennifer

Schnermann, Martin J.

Schnitzer, Moritz

Schnoll, Robert

Schoder, Heiko

Schofield, Penelope

Scholl, Isabelle

Schonberg, Mara

Schootman, Mario

Schott, Sarah

Schousboe, John T.

Schrag, Deborah

Schramm, Dominik

Schraw, Jeremy

Schreiber, Stefan

Schrock, Alexa

Schrump, David S.

Schuetz, Philipp

Schuh, A.

Schulman, S.

Schulte, Fiona S. M.

Schulte, Johannes H.

Schultz, Nikolaus

Schumacher, Fredrick

Schunemann, Holger J.

Schuster, Gene

Schutz, Fabio

Schvartsman, Gustavo

Schwabe, Robert

Schwartz, Kendra

Schwartz, Lisa

Schwartzbaum, Judith

Schwartzberg, Lee

Schwarz, Julie

Schwebel, David C.

Schwedhelm, Carolina

Schwenkglenks, Matthias

Schwingshackl, Lukas

Scoggins, Charles R.

Scognamiglio, GiosuÃ¨

Scorilas, Andreas

Scorsetti, Marta

Scott, David

Scott, Rodney

Seagle, Brandon‐Luke

Sebastian, Aimy

Sebastiani, Federica

Sedokani, Amin

See, Lai‐Chu

Seeley, Dugald

Segel, Joel

Segelov, Eva

Segnan, Nereo

Segrin, Chris

Sehgal, Kartik

Seibert, Julia

Seidel, Christoph

Seifart, Ulf

Seifert‐Klauss, Vanadin

Seiler, Annina

Seiler, Roland

Seino, Kenichiro

Seishima, Jun

Seitz, Stephan

Seki, Akihiro

Seki, Ekihiro

Seki, Naohiko

Seki, Nobuhiko

Sekino, Yohei

selek, ugur

Self, Wesley H.

Selmer, Christian

Semczuk, Andrzej

Semple, Cherith

Senan, Suresh

Sendi, Parham

Sendur, Mehmet A. N.

Seneviratne, Sanjeewa

Seng, Jingjing

Senghore, Thomas

Sengupta, Aditya

Senkomago, Virginia

Senkus, Elzbieta

Senore, Carlo

Senter, Leigha

Senter, Peter D.

Seo, Dong Wan

Seo, Ill Young

Seo, Jeong‐Sun

Seo, Kwon‐Il

Seo, Seong Il

Seo, Sung wook

Seo, Susan

Seo, Woo‐Keun

Seoane, Joan

Seow, Choon Sheong

Seow, Choon‐Sheong

Seppala, Jan

Sequist, Lecia

Serebruany, Victor

Serizawa, Hiroshi

Serody, Jonathan

Serpa, Jacinta

Serra, Massimo

Serrano, Cesar

Sert, Fatma

Serth, Jürgen

Sesto, Mary E.

Seth, Tulika

Sethi, Gautam

Seto, Masao

Seto, Yasuyuki

Seufferlein, Thomas

Sevdalis, Nick

Sewell, William

Seymour, John

Sgambato, Alessandro

Sha, Dan

Shabason, Jacob

Shafrin, Jason

Shah, Bijal

Shah, Binay Kumar

Shah, Gunjan

Shah, Jatin

Shah, Manish

Shah, Nina

Shah, Nisha

Shah, Rachna

Shaikh, Mushfiq

Shaikh, Talha

Shair, Kathy H. Y.

Shalata, Walid

Shan, Guangliang

Shan, Jingxuan

Shan, Li

Shan, Yan‐Shen

Shanafelt, Tait D.

Shang, Changzhen

Shang, Jun

Shankar, Eswar

Shao, Genze

Shao, Chunlin

Shao, Fang

Shao, Guoguang

Shao, Jianyong

Shao, Shiqing

Shao, Shujuan

Shao, Xianhao

Shao, Zengwu

Shao, Zhimin

Shao, Zhi‐Ming

Shapiro, Charles

Shapiro, Gilla

Sharaf, Ravi

Sharbeen, George

Shariat, Shahrokh

Shariat, Shahrokh F.

Sharma, Amod

Sharma, Atul

Sharma, Dipali

Sharma, Hari Shanker

Sharma, Sumit

Sharp, Rebecca

Sharp, Stephen J.

Sharpe, Daniel

Sharpe, Linda

Shastri, Surendra

Shaundfield, Sara

Shaw, Alice

Shawcross, Debbie Lindsay

Shay, Aubree

She, Jin‐Xiong

Sheahan, Kieran

Shehata, M.

Shekelle, Paul G.

Sheltzer, Jason M.

Shen, Chan

Shen, Chenyang

Shen, Chen‐Yang

Shen, Chuanlu

Shen, Feng

Shen, Fu

Shen, Guohua

Shen, Haixiang

Shen, Hongbing

Shen, Hua

Shen, Lei

Shen, Liang Fang

Shen, Liangfang

Shen, Lin

Shen, Peilin

Shen, Shaoping

Shen, Shen

Shen, Shunli

Shen, Xian

Shen, Xiaobing

Shen, Xiaoli

Shen, Yanyun

Shen, Yaqi

Shen, Ye

Shen, Yi

Shen, Ying

Shen, Yongchun

Shen, Zhanlong

Shen, Zhen‐Bin

Shen, Zhoujun

Sheng, Wang‐Huei

Sheng, Weiqi

Sheng, Yao

Shepherd, Heather

Shepherd, Frances

Sheppard, Vanessa B.

Shepshelovich, Daniel

Sher, David J.

Sher, Farooq

Sherafatian, Masih

Sherman, Mark E.

Sherratt, R. Simon

Sheth, Siddharth

Shevde, Lalita

Shi, Xianquan

Shi, Bingyin

Shi, Du

Shi, Guo‐Hai

Shi, Hai‐Bin

Shi, Hanping

Shi, Jianxiang

Shi, Ji‐Min

Shi, Jingyun

Shi, Ju‐Fang

Shi, Jumei

Shi, Keqing

Shi, Lingyan

Shi, Liye

Shi, Lizheng

Shi, Min

Shi, Qiyun

Shi, Shen

Shi, Si

Shi, Tingyan

Shi, Xianbiao

Shi, Yinghong

Shi, Yuankai

Shiba, S.

Shibata, David

Shida, Dai

Shields, Carol

Shiels, Meredith

Shien, Tadahiko

Shigeoka, Manabu

Shih, Jin‐Yuan

Shih, Ya Chen Tina

Shim, Hyo Sup

Shim, Ju Hyun

Shim, Young Mog

Shimada, Kazuyuki

Shimada, Yasuhiro

Shimazu, Taichi

Shimizu, Kimihiro

Shimizu, Nahoko

Shimizu, Satoshi

Shimizu, Yuichi

Shimoi, Tatsunori

Shimokawa, Tsuneo

Shin, Dong Wook

Shin, Hee Young

Shin, Jae‐Ho

Shin, Jung Hee

Shin, Ju‐Young

Shin, Kunyoo

Shin, Kyung Hwan

Shin, Sung Wook

Shindo, Takero

Shinozaki, Eiji

Shinto, Ajit

Shiota, Masaki

Shioyama, Yoshiyuki

Shiozawa, Toshihiro

Shipley, Janet M.

Shirabe, Ken

Shirai, Katsuyuki

Shirakawa, Yasuhiro

Shirazipour, Celina

Shiroishi, Mark S.

Shiroyama, Takayuki

Shlipak, Michael

Shmulevich, Illya

Sho, Masayuki

Shong, Young Kee

Shore, Neal

Short, Nicholas

Shortt, J.

Shouval, Roni

Showalter, Timothy N.

Shpall, Elizabeth

Shrikande, Shailesh

Shu, Jian

Shu, Jiang

Shu, Xiao Ou

Shukla, Sanjeev

Shukla, Sudhanshu Kumar

Shulman, David

Shum, Hoi Ping

Shuman, Marc

Si, Yi

Si, Yiran

Sibon, David

Sica, Antonello

Siddiqi, Mushtaq A.

Siddique, Hifzur

Siddiqui, Imran

Siddiqui, Mohummad Minhaj

Siddiqui, Salahuddin

Siddiqui, Sumaira

Sidey‐Gibbons, Chris J.

Sidiropoulou, Zacharoula

Siebenhuener, Alexander

Siegel, Rebecca

Siegmund, Kimberly

Siemens, D. Robert

Siena, Salvatore

Sier, Cornelis

Sigala, Sandra

Sigel, Keith

Signoretti, Sabina

Signorovitch, James

Sikander, Mohammed

Silberfein, Eric

Silk, Ann

Silove, Derrick

Silva, Amélia M.

Silva, Oscar

Silveira, Augusta

Silver, Michelle I.

Silverman, Lewis

Silvestris, Nicola

Simmons, Christine

Simms, Kate

Simon, Greg

Simon, Gregory E.

Simon, Itamar

Simon, Michael

Simons, Martijn

Simpson, Richard

Sims, Andrew

Sina, Christian

Sineshaw, Helmneh M.

Singal, Amit G.

Singer, S.

Singh, Arjun

Singh, Naveena

Singh, Navneendra

Singh, Pankaj

Singh, Parminder

Singh, Pawan

Singh, Preet Paul

Singh, Rajvinder

Singh, Ratnakar

Singh, Sheila K.

Singh, Sunny R. K.

Singhal, Sunil

Sinicrope, Frank

Sinsky, Christine A.

Sirinukunwattana, Korsuk

Sise, Meghan

Sitarz, Robert

Sittig, Dean F.

Siu, Lillian

Sivapiragasam, Abirami

Siwawa, Portia

Skaali, Tone

Skaane, Per

Skala, Melissa C.

Skarphedinsson, Gudmundur

Skinner, Heath

Skoetz, Nicole

Skoulidis, Ferdinandos

Skriver, Charlotte

Slack, Frank

Slattery, Martha

Slavin, Thomas

Sledge, George W.

Śledziński, Paweł

Sleight, Alix

Sleijfer, Stefan

Sloan, Erica K.

Sloane, Bonnie

Slyskova, Jana

Smaldone, Marc

Small, Eric J.

Smani, Tarik

Smedby, Karin

Smetana, Karel

Smets, Ellen M. A.

Smit, Amelia K.

Smit, Egbert

Smit, Egbert F.

Smit, Linda

Smith, Adam J. de

Smith, Angela

Smith, Benjamin

Smith, Brendan

Smith, David

Smith, Ellen

Smith, Jennifer

Smith, Malcolm A.

Smith, Megan Alexandra

Smith, Robert

Smith, Sian

Smith, Sonali M.

Smith, Sophia

Smith, Webb

Smith‐Graziani, Demetria

Smyth, Elizabeth

Smyth, Elizabeth C.

Snaebjornsson, Petur

Snaman, Jennifer

Snell, Russell G.

Sniadecki, Marcin

Snider, James

Snook, Adam E.

Snyder, Jeremy

Snyper, Nana Yaa

Soares, Flvia Moreira

Soares, Heliosa

Soares, Leonardo

Sobotka, Stanislaw

Sodergren, Mikael H.

Soehngen, Eric

Soga, Tomoyoshi

Søgaard, Mette

Soh, Hosim

Soh, Junichi

Sohn, Jin Hee

Sohn, Sang Kyun

Solanki, Abhishek

Solano, Angela

Solano, Carlos

Solassol, Jérôme

Soliman, Sameh

Solit, David

Sollini, Martina

Solomon, Ben

Soltermann, Alex

Somaiah, Neeta

Somashekhar, S. P.

Sommariva, Michele

Sommers, Benjamin D.

Somsouk, Ma

Son, Eun Ju

Son, Ji Woon

Son, Jieun

Sondermann, Wiebke

Soneji, Samir

Sonenberg, Nahum

Song, Chuan

Song, Chuangui

Song, Ci

Song, Dianwen

Song, Dong Eun

Song, Er‐Wei

Song, Fangzhou

Song, Fengju

Song, Geum Jong

Song, Gi‐Won

Song, He

Song, Huan

Song, Jukun

Song, Kehan

Song, Kun

Song, KY

Song, Lele

Song, Myeong Jun

Song, Pan

Song, Rui

Song, Sang Hun

Song, Shaoli

Song, Shibo

Song, Shiyu

Song, Si Yeol

Song, Tianqiang

Song, Wei

Song, Wenjing

Song, Xiangping

Song, Xianrang

Song, Yong

Song, Yong‐Sheng

Song, Yongxi

Song, Yu

Song, Yuqin

Song, Zhengshuai

Song, Zhiwang

Soni, Sourabh

Sonpavde, Guru

Soo, Ross

Sood, Anil

Sood, Nitin

Sooriakumaran, Prasanna

Sørensen, Michala

Sorensen, Poul

Soria, Francesco

Sorlie, Therese

Sosa, Julie A.

Sosman, Jeffrey A.

Soto, José‐Luis

Sotomayor, Eduardo

Soukup, Tayana

Sousa, Diana Aguiar de

Southey, Melissa

Souza, Paul

Spadaccini, Marco

Spadafora, Marco

Spagnolo, Francesco

Spałek, Mateusz

Spaner, David

Spatari, Giovanna

Spatz Levine, Alison

Spaulding, Anne

Specht, Lena

Spector, Logan

Spector, Logan G.

Speers, Corey

Speich, Benjamin

Spencer, Llinos Haf

Sperati, Francesca

Sperr, Wolfgang

Spiegel, David

Spiegel, Sarah

Spiegelhalder, Kai

Spiess, Philippe E.

Spitaleri, Gianluca

Spivack, Simon

Spivak, Jerry Le Pow

Spolverato, Gaya

Spoozak, Lori

Sposito, Carlo

Spratt, Daniel

Spratt, Heidi

Spring, Kevin

Spronk, P.

Srinivasan, Radhika

Sriplung, Hutcha

Srivastava, Deo

St Germain, Diane

St John, Maie

St. Clair, Caryn

St. Clair, Daret

Stacchiotti, Silvia

Stacey, Dawn

Stadler, Rudolf

Stahl, Michael

Stahl, Michael J.

Stambaugh, Cassandra

Stanford, Janet

Stanisławek, Andrzej

Stanulla, Martin

Stark, Mitchell

Starkova, Julia

Starling, Naureen

Starmer, Heather

Starr, Timothy K.

Startsev, Vladimir

Stathopoulos, Georgios

Stavropoulos, S. William

Steeghs, Neeltje

Steele, Andrew

Steele, Megan

Steele, R. J. C.

Steenbergen, Renske

Steenbergen, Renske D. M.

Steensma, David

Stefanick, Marcia

Steffen, Annika

Steigen, Sonya

Stein, Alexander

Stein, Doron

Stein, Ulrike

Steliarova‐Foucher, Eva

Stelljes, Matthias

Stenzel, Ashley

Stenzl, Arnulf

Sterzing, Florian

Steuer, Conor

Stevanovic, Jasmina

Stevens, Jennifer

Stevenson, J. D.

Stewart, Grant D.

Stewart, Mary

Steyerberg, Ewout W.

Stier, Elizabeth

Stiggelbout, Anne M.

Stintzing, Sebastian

Stitzlein, Russ

Stitzlein, Russell

Stock, MD, Wendy

Stockmann, Christian

Stoelzel, Friedrich

Stofilova, Jana

Stoitzner, Patrizia

Stolfi, Carmine

Stolnicu, Simona

Stone, Nelson

Stone, Patrick

Stoneham, Sara

Storti, Paola

Stout, Natasha K.

Stowell, Justin

Stoyanova, R.

Strand, Fredrik

Stratigos, A. J.

Straus, David

Street, Richard L.

Streiff, Michael

Strickland, Stephen

Strickler, John H.

Strobel, Oliver

Stroomberg, Hein

Strosberg, Jonathan R.

Stuart, Elizabeth A.

Stubbs, Brendon

Stuber, Margaret

Studts, Jamie

Stupp, Roger

Sturgis, Erich

Sturm, Rick

Styczyński, Jan

Styczynski, J.

Styles, Lori

Su, Chien‐Wei

Su, Chunxia

Su, Hong

Su, Jun

Su, Liping

Su, Shi‐Bing

Su, Tung‐Hung

Su, Wu‐Chou

Su, Xiangqian

Su, Xiaohui

Su, Yunan

Su, Yu‐Ru

Su, Yuxiong

Subasri, Mathushan

Subat, Sophia

Subbiah, Vivek

Subramanian, Kritika

Suchon, Pierre

Suchorska, Wiktoria

Suda, Kenichi

Sudan, Sarabjeet

Sudore, Rebecca

Sugai, Tamotsu

Sugano, Koji

Sugarbaker, Paul

Sugaya, Akinori

Sugaya, Makoto

Sugihara, Hiroyuki

Sugihara, Kenichi

Sugisaka, Jun

Sugita, Yasuo

Sugiyama, Toshiro

Suh, Cheolwon

Suh, James H.

Sui, Jing

Sui, Weiguo

Sujenthiran, A.

Sukocheva, Olga

Sukumar, Saraswati

Sullivan, Frank

Sullivan, Daniel

Sullivan, Michael

Sullivan, Ryan J.

Sumazin, Pavel

Sun, Caixing

Sun, Can‐lan

Sun, Changgang

Sun, Chaoyang

Sun, Cheng‐Cao

Sun, Chien‐An

Sun, Chuan‐Zheng

Sun, Daju

Sun, Da‐wei

Sun, Deqiang

Sun, Guangyi

Sun, Haopeng

Sun, Hong

Sun, Hongliang

Sun, Hui

Sun, Huichuan

Sun, James

Sun, Jianguo

Sun, Jiayuan

Sun, Jia‐Yuan

Sun, Jichun

Sun, Jie

Sun, Jing

Sun, Jong‐Mu

Sun, Junhui

Sun, Jun‐Hui

Sun, Kai

Sun, Leitao

Sun, Li

Sun, Min

Sun, Ming

Sun, Mingjun

Sun, Nan

Sun, Ping‐Li

Sun, Qiang

Sun, Ranran

Sun, Rui

Sun, Shi‐Yong

Sun, Tianwei

Sun, Wei

Sun, Wei‐Liang

Sun, Xianzhi

Sun, Xiao

Sun, Xiaochun

Sun, Xiao‐Qiang

Sun, Xibin

Sun, Xin

Sun, Xue‐Song

Sun, Xueying

Sun, Xuhong

Sun, Yandi

Sun, Yifeng

Sun, Yihua

Sun, Ying

Sun, Yu

Sun, Zhaohui

Sun, Zhi‐Jun

Sun, Zhonghua

Sunaga, Noriaki

Sunagozaka, Hajime

Sunakawa, Yu

Sundahl, Nora

Sundar, Raghav

Sundar, Sudha

Sundboll, Jens

Suneja, Gita

Sung, J.S

Sung, Joseph

Sung, Ki Woong

Sung, Lillian

Suo, Chen

Sura, Gloria

Suresh, Amritha

Surette, Michael G.

Suryakiran, Pemmaraju

Sussulini, Alessandra

Sutradhar, Rinku

Suzuki, Eiichiro

Suzuki, Hidekazu

Suzuki, Hidenori

Suzuki, Kazuhito

Suzuki, Masaru

Suzuki, Mikio

Suzuki, Ritsuro

Suzuki, Sadao

Svatek, Robert

Swain, Sandra M.

Swami, Umang

Swarbrick, Alexander

Swartz, Justin

Sweeney, Carol

Sweileh, Waleed

Swiecicki, Paul L.

Swisher‐McClure, Samuel

Swoboda, David

Swords, Douglas S.

Syahir, Amir

Syaj, Sebawe

Sykes, DeLawrence

Sylvester, Richard J.

Symeonidis, Argiris

Symonds, Erin

Syngal, Sapna

Syriac, Arun

Syrigos, Kostas

Szablewski, Vanessa

Szalda, Dava

Sze, Marc

Szlosarek, P. W.

Szmit, Sebastian

Szturz, P.

Szuhai, Karoly

Szumera‐Cieckiewicz, Anna

Ta, De‐an

Taarnhøj, Gry

Tabatabaiefar, Mohammad Amin

Tabernero, Josep

Tabernero, María

Taborelli, Martina

Tabori, Nora E.

Taccioli, Cristian

Tachezy, Ruth

Tada, Toshifumi

Tadi, Surender

Tagawa, Scott

Tagawa, Tetsuzo

Tagliabue, Elda

Tagliabue, Marta

Tahara, Tomomitsu

Tai, Eric

Tai, Sheng

Tai, Yi

Tai, Yu‐Tzu

Taieb, Julien

Taigo, Kato

Taioli, Emanuela

Tai‐Seale, Ming

Taizo, Hibi

Taji, Bahareh

Tajiri, Kazuto

Tajiri, Takuma

Takabe, Kazuaki

Takada, Kazuki

Takagi, Toshio

Takahara, Taishi

Takahari, Daisuke

Takahashi, Atsushi

Takahashi, Hidenori

Takahashi, Hiroyuki

Takahashi, Koichi

Takahashi, Naoto

Takahashi, Shunji

Takahashi, T.

Takamatsu, Hiroyuki

Takami, Akiyoshi

Takata, Noboru

Takeda, Atsuya

Takefumi, Komiya

Takenoyama, Mitsuhiro

Takes, Robert

Takeshita, Takashi

Taketo, M. Mark

Takeuchi, Hiroshi

Takeuchi, Kengo

Takeya, Motohiro

Takiguchi, Yuichi

Takizawa, Jun

Taku, Nicolette

Talamantes, Sarah

Talasaz, AmirAli

Taly, Valerie

Tam, Moses

Tamai, Toshikatsu

Tamai, Tsutomu

Tamayo, Pablo

Tamiya, Akihiro

Tamiya, Motohiro

Tamura, Kazuo

Tamura, Tomohide

Tamura, Tomohiro

Tan, Aik Choon

Tan, Benjamin

Tan, Daniel

Tan, E‐Jean

Tan, Li

Tan, Lijie

Tan, Puay Hoon

Tan, Shengkui

Tan, Ya

Tanaka, Hiromasa

Tanaka, Hisashi

Tanaka, Masamitsu

Tanaka, Shinji

Tanaka, Takuji

Tanaka, Teruhisa

Tang, An

Tang, Cai‐Xi

Tang, Chih Hsin

Tang, Chung‐Ngai

Tang, Daolin

Tang, Dehua

Tang, Hailin

Tang, Hexiao

Tang, Hong

Tang, Hua

Tang, Huifang

Tang, Jiajia

Tang, Jianming

Tang, Jing

Tang, JinHai

Tang, Junfang

Tang, Lei

Tang, Min

Tang, Sangsang

Tang, Siew Tzuh

Tang, Wanxin

Tang, Weiming

Tang, Weizhong

Tang, Yanlai

Tang, Yongjian

Tang, Zhen

Tang, Zuxiong

Tani, Joji

Taniguchi, Hiroya

Tanioka, Hiroaki

Taniyama, Yusuke

Tanz, Rachid

Tao, Lei

Tao, Liyuan

Tao, Qian

Tao, Shengru

Tao, Wensi

Tao, Yiming

Tao, Yongguang

Tao, Yungan

Tao, Zhihua

Tap, William

Taplin, Mary Ellen

Taplin, Mary‐Ellen

Tarasenko, Yelena N.

Targher, Giovanni

Tarhini, Ahmad

Tariq, Amara

Tartaglia, Nicola

Tarumi, Yoko

Tasken, Kjetil

Tasneem, Abbas Ali

Tastuo, Kanda

Tatalovich, Zaria

Tateishi, Ryosuke

Tattevin, Pierre

Tauriello, Daniele V. F.

Tavana, Hossein

Tavano, Francesca

Tavassoli, Mahvash

Tavassoly, Iman

Taylor, Justin

Taylor, Marianne

Taylor, Michael

Taylor, Natalie

Taylor, Philip R.

Taylor, Rachel

Teame, Hirut

Tedeschi, Alessandra

Teepen, Jop C.

Teh, Bin

Tehrani, Hossein Abdul

Teimoori‐Toolabi, Ladan

Teishima, Jun

Teixeira, Ana L.

Teixeira, Marcela

Teixeira, Natercia

Temel, Jennifer

Temiz, Bilal Esat

Templeton, Arnoud J.

Temraz, Sally

Teng, Haibo

Teng, Honglin

Teng, Jiamin

Teng, Lisong

Teng, Zhongzhao

Tepper, Clifford

Terazawa, Tetsuji

Terezakis, Stephanie A.

Termrungruanglert, Wichai

Terpos, Evangelos

Terracciano, Luigi

Terrenato, Irene

Terry, Stéphane

Tersak, Jean

Tervonen, Hanna

Tessema, Gizachew Assefa

Tevaarwerk, Amye

Tew, Kenneth

Thakur, Nisha

Thakur, Satbir

Thall, Peter F.

Thalmann, George N.

Thambamroong, Tawasapon

Tharavichtikul, Ekkasit

Thastrup, Jacob

Thawani, Rajat

Theilacker, Christian

Thein, Hla‐Hla

Theodoratou, Evropi

Theodorescu, Dan

Theodoropoulos, Panayiotis

Thibault, Constance

Thibodeau, Stephen

Thieblemont, Catherine

Thiede, Christian

Thiery‐Vuillemin, Antoine

Thiyagarajan, Varadharajan

Thomas, Alissa

Thomas, Brian F.

Thomas, Gareth

Thomas, George V.

Thomas, Hannah

Thomas, Lewis J.

Thomas, Luc

Thomas, Xavier

Thomford, Nicholas E.

Thompson, Deborah

Thompson, Erik

Thompson, Ian

Thomsen, Reimar

Thomusch, Oliver

Thong, Melissa

Thorat, Mangesh

Thorgeirsson, Snorri S.

Thorkildsen, Joachim

Thoudam, Themis

Threatt, Stevie

Thrift, Aaron

Thumallapally, Nishitha

Thunnissen, Erik

Thurairaja, Ramesh

Thurnher, D.

Tian, Daofeng

Tian, Hui

Tian, Jie

Tian, Junqiang

Tian, Rong

Tian, Sibo

Tian, Tieshuan

Tian, Xuefei

Tian, Yantao

Tian, Zelin

Tian, Ziqiang

Tiao, Gregory

Tice, Jeffrey A.

Tieu, Alan

Tilling, Kate

Tilly, Hervé

Timofeeva, Maria

Timpson, Paul

Timsit, Marc‐Olivier

Tin, Sandar Tin

Tinay, Ilker

Tindle, Hilary A.

Ting, Angela

Tiribelli, Mario

Tirumani, S. H.

Tiseo, M.

Tiseo, Marcello

Titano, Joseph J.

Tjonneland, Anne

To, Ka‐Fai

Toben, Catherine

Tobinai, Kensei

Tocchetti, Carlo Gabriele

Todd, John A.

Toi, Yukihiro

Toiyama, Yuji

Tokuhira, Michihide

Tokuhisa, Motohiko

Tolat, Parag

Toledo, Diogo O.

Tollens, Fabian

Tollosa, Daniel

Tomao, Silverio

Tomasini, Pascale

Tomita, Naohiro

Tomita, Natsuo

Tomizawa, Daisuke

Tommasi, S.

Toner, Mehmet

Tong, Junwei

Tong, Lingying

Tong, Yuexin

Tonyali, Onder

Toohey, Kellie

Toomey, Sinead

Topatana, Win

Topolcan, Ondrej

Toraih, Eman

Torigoe, Toshihiko

Toriola, Adetunji

Tornesello, Maria Lina

Torres‐Saavedra, Pedro

Torti, Suzy V.

Tosar, Juan

Toscani, Denise

Toshima, Fumihito

Toslak, Iclal Erdem

Tost, Joerg

Tosteson, Anna N. A.

Touati, Nathan

Touboul, Cyril

Tous, Sara

Tousseyn, Thomas

Touvier, Mathilde

Tovoli, Francesco

Towle, Kevin

Townsend, Amanda

Toya, Takumi

Toyoda, Hidenori

Trabert, B.

Trajanoski, Zlatko

Tramontano, Angela

Tran, Nikki

Tran, Phuoc

Tran, Thach Duc

Tran, Thai

Trangas, Theoni

Trapero‐Bertran, Marta

Trappe, Ralf Ulrich

Travaglino, Antonio

Travis, William

Tree, Alison

Tremblay, Dominique

Tremblay, Douglas

Trent, Jonathan

Trentham‐Dietz, Amy

Trevellin, Elisabetta

Trevino, Jose

Trevisani, Franco

Triantafyllou, Konstantinos

Trigo, Jose

Trimble, Edward

Trimboli, P.

Trimboli, Pierpaolo

Trinchieri, Giorgio

Triozzi, Pierre

Tripodo, Claudio

Trock, Brunce

Troester, Melissa

Troiano, Giuseppe

Troncone, Giancarlo

Trump, Donald

Trussardi Fayh, Ana Paula

Tsai, Li‐Yu

Tsai, Chun‐Ming

Tsai, Donald E.

Tsai, Kun‐Ling

Tsai, Te‐Fu

Tsai, Tsai‐Fu

Tsai, Ying‐Huang

Tsang, Ngan‐Ming

Tsang, Richard W.

Tsao, May

Tsao, Ming‐Sound

Tschudin, Sibil

Tse, Lap Ah

Tseng, Ailun

Tseng, Chia‐Lin

Tseng, Ling‐Ming

Tseng, Yau‐Lin

Tsibulak, Irina

Tsikouras, Panagiotis

Tsilidis, Konstantinos

Tsivgoulis, George

Tsodikov, Alexander

Tsou, Yung‐Kuan

Tsourlakis, Maria

Tsubata, Yukari

Tsuchida, Keisuke

Tsuchikawa, Takahiro

Tsuchiya, Naoto

Tsuji, Akihito

Tsuji, Kunihiro

Tsujimura, Tohru

Tsukamoto, Tetsuya

Tsunoda, Shigeru

Tsurutani, Junji

Tsushima, Takahiro

Tu, Benjamin

Tu, Chih‐Yen

Tu, Haijun

Tu, Shuiping

Tubin, S.

Tuite, Catherine

Tumedei, Maria

Tung, Nadine

Tupikowski, Krzysztof

Tural, Deniz

Turan, Turgay

Turan, Umit

Turati, Federica

Turcotte, Lucie M.

Turic, Bojana

Turk, Murat

Turkbey, Baris

Turkina, Anna

Turnbull, Clare

Turner, Nicholas

Turpie, Alexander G. G.

Turpin, Anthony

Tuveson, David

Tverdek, Frank

Twigg, Joshua

Tye, Coralee E.

Tyrovolas, Stefanos

Tzaridis, Theophilos

Tzeng, Ching‐wei

Tzeng, Ching‐Wei D.

Tziatzios, Georgios

Ubels, Joske

Uchida, Mayako

Uchida, Taro

Uchino, Junji

Uddin, Faizan

Udumyan, Ruzan

Udupa, Karthik

Ueda, K.

Ueno, Hideki

Ueno, Makoto

Uetake, Hiroyuki

Ugalde, Anna

Uhara, Hisashi

Uhlén, Per

Uhlig, Annemarie

Uitterlinden, André

Ukimura, Osamu

Uldrick, Thomas S.

Ulivi, Paola

Ullal, Jagdish

Ullrich, Chrisina

Ulrich, Alexis

Ulrich, Cornelia

Um, Hong‐Duck

Umapathy, Ganesh

Umemura, Yoshie

Umeno, Hirohito

Umezawa, Rei

Unciti‐Broceta, Asier

Undas, A.

Unertl, Kim M.

Unger, Elizabeth R.

Unger, Joseph

Unger, Kristian

Unger‐Saldaña, Karla

Unnikrishnan, A.G

Unno, Tatsuya

Uno, Yoshiharu

Upadhyay, Arun

Uppaluri, Ravindra

Urabe, Masayuki

Urban, Margaret

Urbanek, Tomasz

Ureshino, Hiroshi

Urquhart, R.

Usher‐Smith, Juliet

Ushijima, Toshikazu

Usmani, Saad Z.

Ustun, Celalettin

Utikal, Jochen

Uyl‐de Groot, Carin

Vaccarella, Salvatore

Vaccaro, Carlos

Vadaparampil, Susan

Vadgama, Jaydutt V.

Vahidnezhad, Hassan

Vahrmeijer, Alex

Vaidya, Jayant S.

Vainchenker, William

Vaishampayan, Ulka

Vajda, György

Valachis, Antonis

Valdimarsdottir, Unnur

Vale, Diama

Valenti, David

Valenti, Luca

Valer, Cristina

Valiente González, Laura

Valle, Laura

Valverde, Claudia

van Dam, Coen

Van de Louw, Andry

van de Ven, Steffi

van de Wetering, Marianne D.

Van den Begin, Robbe

van den Brekel, Lieke

van den Brink, Gijs R.

van den Brink, Marcel R. M.

Van den Wyngaert, T.

van der Geest, Lydia

van der Graaf, Professor Winette

van der Graaf, Winette

Van Der Hoeven, Jacobus JM

van der Hulle, T.

van der Kolk, Marion

van der Lee, Marije

van der Pol, Simon

van der Sijde, Fleur

van der Velden, N.C

van der Wekken, Anthonie

van Diest, Paul

Van Dongen, Jacques J.M

van Eijck, Casper H.

Van Engen, Ruben

Van Gool, Stefaan W.

Van Haele, Matthias

Van Hemelrijck, Mieke

van Laarhoven, Hanneke

van Leerdam, Monique

van Leeuwen, Flora E.

Van Mater, David

van Niekerk, Dirk

van Osch, Frits

van Soest, Robert J.

Van Swearingen, Amanda

van Tilburg, Cornelis

Van Tine, Brian

van Tol‐Geerdink, Julia

van Vliet, Liesbeth

Vancleave, Janet

Vandekerckhove, Kristof

Vanderpool, Robin

Vandin, Fabio

Vandrey, Ryan

Vang, Russell

Vano, Yann‐Alexandre

Vanoli, Alessandro

Vapiwala, Neha

Varadhachary, Gauri

Vardhana, Santosha A.

Vargas, Pablo Agustin

Vargo, John A.

Varvares, Mark

Vasile, Enrico

Vasileiou, Katerina

Vasilevskis, Eduard E.

Vassella, Erik

Vastrad, Basavaraj

Vasudevan, Sanjeev

Vaughan, Thomas L.

Vauthey, Jean‐Nicolas

Vaux, David

Väyrynen, Juha

Vedovati, Maria Cristina

Veenstra, Christine

Veer, Hans Van

Veeraraghavan, Harini

Veerman, Anjo JP

Vegnenegre, Alain

Veitch, Zachary

Velasco, Guillermo

Velcheti, Vamsidhar

Velculescu, Victor

Velde, CJH van de

Velenik, W.

Velikova, Galina

Vellenga, Edo

Velmahos, Constantine

Veloso de Oliveira, Julia

Venn, Alison J.

Ventura, Marco

Ventz, Steffen

Venuti, Aldo

Vergani, Francesco

Vergara, Daniele

Verhaak, Roel

Verhaegh, Gerald

Verheij, Marcel

Verhoeff, Joost

Verhoeven, Guido

Verma, Chandan

Verma, Meghna

Verma, Sunil

Verma, Vivek

Vermaat, Joost

Vermi, William

Vermorken, Jan B.

Vermunt, Marit

Vernerey, Dewi

Vernieri, Claudio

Vernon, Sally

Versluis, Jurjen

Vesole, David H.

Viazis, Nikos

Vibert, Eric

Vicini, Frank

Vickers, Andrew

Vidal, Adriana

Vidal‐Sicart, Sergi

Videtic, GM

Vieira, Ana Rita

Viers, Boyd R.

Vijayakumar, DK

Vijayvargiya, Prakhar

Vikramdeo, Kunwar Somesh

Vilar, Eduardo

Villano, John

Villanti, Andrea C.

Villanueva, Augusto

Vimercati, Luigi

Vinciguerra, Manlio

Vineis, Paulo

Vinh‐Hung, Vincent

Viola, David

Viprey, Marie

Virgo, Katherine S.

Visco, Carlo

Viscusi, Eugene

Visekruna, Alexander

Visseren, Frank

Vissers, Pauline

Visvanathan, Kala

Viswanath, Satish

Viswanathan, A.

Vital, Marius

Vitali, Francesca

Vito, Alyssa

Vivas‐Mejia, Pablo E.

Vo, Kieuhoa

Vogel, Christine

Vogel, Rachel

Vogelzang, Nicholas

Vogtmann, Emily

Vohra, Sanah

Vohra, Sunita

Vokes, Everett

Vokinger, Kerstin

Volinia, Stefano

Volk, Robert

Vollmuth, Philipp

von Blanckenburg, Pia

von Buchwald, Christian

von Itzstein, Mitchell

Voogd, Adri

Voogd, Adri C.

Voorhees, Timothy

Vos, Theo

Vose, Julie

Voso, Maria Teresa

Voss, Amartin H.

Vranic, Semir

Vrdoljak‐Mozetic, Danijela

Vreeland, Timothy

Vrieling, Alina

Vries, Esther de

Vu, Hoang Anh

Vuong, Te

Vymetalkova, Veronika

Wada, Hisashi

Wada, Naoki

Wada, Yoshiyuki

Wadia, Roxanne

Wager, M.

Wagland, Richard

Wagner, Lars

Wagner, Michael

Wagner, Monika

Wahafu, Wasilijiang

Wahed, Amer

Wahlin, Bjorn Engelbrekt

Wainberg, Zev

Wainwright, Derek

Wainwright, Derek A.

Wakae, Kousho

Wakatsuki, Masaru

Wakefield, Claire

Wakelee, Heather

Wakimoto, Hiroaki

Wakita, Akiyuki

Wakizaka, Kazuki

Wakkee, Marliess

Walch, Gabrielle

Wålinder, Göran

Walker 2nd, William H.

Walker, Joan L.

Walkey, Allan J.

Wallace, Kristin

Wallana, Williams

Wallentin, Lars

Waller, Amy

Wallner, Lauren P.

Walraven, Iris

Walraven, Janneke

Walsh, Karin

Walter, Fiona

Walter, Roland

Walter, Thomas

Walter, Viola

Walthall, Helen

Walts, Ann E.

Wan, Changshan

Wan, Desen

Wan, Fengyi

Wan, Jinghai

Wan, Jingyuan

Wan, Junhu

Wan, Qi

Wan, Yi

Wan, Yinsheng

Wan, Zheng

Wang, Wei

Wang, Anxun

Wang, Baojun

Wang, Baoshan

Wang, Biao

Wang, Bing Yen

Wang, Biyun

Wang, Che

Wang, Chen

Wang, Chendong

Wang, Cheng

Wang, Chenyang

Wang, Chong

Wang, Chongkai

Wang, Chuanxin

Wang, Chun

Wang, Chunsheng

Wang, Cun

Wang, Cun‐Yu

Wang, Daifeng

Wang, Dan

Wang, Danbo

Wang, Daniel Y.

Wang, Dawei

Wang, Debin

Wang, Dehui

Wang, Delin

Wang, De‐shen

Wang, Dian

Wang, Dong

Wang, Dongqing

Wang, Eunice S.

Wang, Fan

Wang, Fang‐Tao

Wang, Fei

Wang, Feiran

Wang, Feng

Wang, Gang

Wang, Geng

Wang, Guangke

Wang, Guangyi

Wang, GuiYing

Wang, Guowen

Wang, Haiwei

Wang, Haiyong

Wang, Hanchu

Wang, Honghui

Wang, Honglei

Wang, Hongsheng

Wang, Hong‐yang

Wang, Huaizhi

Wang, Huaying

Wang, Hung‐Chen

Wang, Jaw‐Yuan

Wang, Jiangfeng

Wang, Jianhua

Wang, Jiani

Wang, Jian‐jun

Wang, Jian‐Liu

Wang, Jianming

Wang, Jianping

Wang, Jianqing

Wang, Jianye

Wang, Jiazhou

Wang, Jie

Wang, Jijia

Wang, Jing

Wang, Jingpu

Wang, Jingwen

Wang, Jinhua

Wang, Ju

Wang, Ju‐Ming

Wang, Jun

Wang, Junqi

Wang, Junye

Wang, Kai

Wang, Kefeng

Wang, Kui

Wang, Kun

Wang, Le

Wang, Lei

Wang, Li

Wang, Liang

Wang, Lie

Wang, Liming

Wang, Lina

Wang, Linghua

Wang, Long

Wang, Mao‐qiang

Wang, Meilin

Wang, Meiling

Wang, Meiyun

Wang, Meng

Wang, Miao

Wang, Moran

Wang, Ning

Wang, Peng

Wang, Peng Wang

Wang, Peng‐Hui

Wang, Pingyu

Wang, Qi

Wang, Qian

Wang, Qiang

Wang, Qianhui

Wang, Qiaoqi

Wang, Quanshi

Wang, Qun

Wang, Ran

Wang, Renjie

Wang, Rhu

Wang, Sanming

Wang, Senming

Wang, Shang‐Jui

Wang, Shaohua

Wang, Sheng

Wang, Shengfeng

Wang, Shengke

Wang, Shixuan

Wang, Shi‐Yi

Wang, Shouhan

Wang, Shu

Wang, Shubin

Wang, Shui

Wang, Shulian

Wang, Sitong

Wang, Sophia

Wang, Tianhu

Wang, Timothy C.

Wang, Tingan

Wang, Tong

Wang, Tzu‐Fei

Wang, Wei

Wang, Weibin

Wang, Wei‐Hu

Wang, Weilin

Wang, Wencai

Wang, Wenming

Wang, Xi

Wang, Xianbo

Wang, Xian‐Bo

Wang, Xiang

Wang, Xiangdong

Wang, Xiangyang

Wang, Xianhuo

Wang, Xiao

Wang, Xiaobo

Wang, Xiaolu

Wang, Xiaonan

Wang, Xiaoshen

Wang, Xiaosheng

Wang, Xiaoyang

Wang, Xin

Wang, Xin Wei

Wang, Xinghuan

Wang, Xinjia

Wang, Xinjun

Wang, Xipeng

Wang, Xiuli

Wang, Xuegang

Wang, Yajie

Wang, Yan

Wang, Yanhong

Wang, Yaning

Wang, Yanqing

Wang, Yanqiu

Wang, Yi

Wang, Yi‐Ching

Wang, Yifeng

Wang, Ying

Wang, Yin‐huai

Wang, Yinsheng

Wang, Yinyan

Wang, Yiqin

Wang, Yonghua

Wang, Yongtao

Wang, Yongzhi

Wang, Youxin

Wang, Yu

Wang, Yuanyu

Wang, Yu‐De

Wang, Yudong

Wang, Yuezhen

Wang, Yun

Wang, Yuying

Wang, Yuzhuo

Wang, Zengjun

Wang, Zhaoxia

Wang, Zhehai

Wang, Zhen

Wang, Zheng

Wang, Zhengge

Wang, Zhenning

Wang, Zhijie

Wang, Zhuangzhuang

Wang, Zihao

Wang, Ziheng

Wang, Ziqiang

Wang, Zi‐Xian

Wang‐Gillam, Andrea

Waning, David

Ward, Doriel

Ward, Kevin

Wardelmann, Eva

Wargo, Jennifer

Warnecke, Richard

Warner, Echo L.

Warner, Jeremy

Warner, Teddy

Warren, Anne Y.

Warth, Marco

Washington, Samuel

Wassersug, Richard

Watabe, Kounosuke

Watanabe, Masahiko

Watanabe, Junichiro

Watanabe, Masayuki

Watanabe, Satoshi

Waterkamp, Daniel

Watson, Peter

Watts, Eleanor

Way, Tzong‐Der

Waziri, Allen

Wearden, Alison

Weaver, Kathryn

Weaver, Sallie

Webb, Myfanwy

Webb, Penelope

Webber, Bryant J.

Weber, Achim

Weber, Jason D.

Weber, Sharon M.

Weberpals, Johanne

Webster, Fiona

Wedding, Ulrich

Wee, Joseph

Wee, Hwee‐Lin

Weekes, Colin

Weerts, M. J. A.

Wei, Dong‐Qing

Wei, Andrew

Wei, Botao

Wei, Changqiang

Wei, Hong

Wei, Jia

Wei, Jianshe

Wei, Jinli

Wei, Lise

Wei, Maria L.

Wei, Minjie

Wei, Po‐Li

Wei, Qi

Wei, Qiang

Wei, Sheng

Wei, Shenhai

Wei, Tao

Wei, Wei

Wei, Wenbin

Wei, Wen‐Jun

Wei, Xi

Wei, Yongyue

Wei, Yue

Wei, Yunwei

Weigert, Andreas

Weinberg, Benjamin

Weiner‐Gorzel, Karolina

Weingart, Saul

Weir, Hannah K.

Weisel, Katja

Weiser, Martin

Weiss, Anna

Weiss, Brian

Weiss, Glen

Weiss, Glen J.

Weiss, Heidi

Weiss, Jennifer

Weiss, Vivian L.

Weissenbacher, Tobias

Weisser, M.

Weitz, Jeffrey

Weitzel, Jeffrey

Weitzel, Jeffrey N.

Weller, Michael

Wells, Alan

Wells, Claire

Wells, Elizabeth M.

Welsh, James

Welt, Anja

Wen, Jiahuai

Wen, Patrick

Wen, Weiping

Wen, Yingsheng

Wender, Richard

Weng, Desheng

Weng, Yingbei

WenJun, Cheng

Wentzensen, Nicolas

Werner, Theresa

Wernli, Karen J.

Wertz, Jasmin

Wessels, Lodewyk F. A.

Wessely, Anja

West, Robert B.

Westenberg, Jos J. M.

Westphalen, C. Benedikt

Whalen, Michael J.

Wheatley‐Price, Paul

Wheeler, Stephanie

Whelan, Jeremy

White, Arica

White, Richard

White, Wesley M.

Whitehall, V. L. J.

White‐Means, Shelley

Whitfield, Jamie

Whitman, Eric

Whop, Lisa J.

Wichmann, Gunnar

Wiemels, Joe

Wiestner, Adrian

Wietrzyk, Joanna

Wijetunga, N. Ari

Wijkstra, Hessel

Wiklund, Peter

Wild, Philipp

Wilder, Julius

Wildes, Tanya

Wilkins, S.

Wilkins, Simon

Willems, Stefan M.

Williams, Amy

Williams, Christina

Williams, Courtney

Williams, Faustine

Williams, Grant R.

Williams, H. C.

Williams, John R.

Williams, Lindsay

Williams, Stephen

Williams, Terence

Wilmott, J. S.

Wilson, Brooke

Wilson, Katherine

Wilson, Richard K.

Wilson, Robert

Wilson, Zachary

Winer, Rachel

Winestone, Lena

Winkler, Eva

Winkler, Frank

Winn, Aaron

Winquist, Eric

Winter, Alexander

Winter, Jean

Winter, Jordan

Winter, Stuart

Winther, Jeanette

Wirsing, Anna

Wisman, G. Bea A.

Witt, Claudia

Witt, Olaf

Wittmann, Daniela

Witzig, Thomas

Woff, Erwin

Wojtukiewicz, Marek Z.

Wolchok, Jedd

Wolden, Suzanne L.

Wolf, Ido

Wolf, Juergen

Wolfe, Heather

Wolfe, Joanne

Wolff, Henri

Wolff, Robert

Wolfgang, Christopher

Wolk, A.

Wolmark, Norman

Wolpin, Brian

Won, Kyu Yeoun

Won, Young‐Joo

Wong, Carmen Chak‐Lui

Wong, Chun‐Ming

Wong, Deborah

Wong, Eric

Wong, Gretel

Wong, Joyce

Wong, Joyce Yuk Ping

Wong, Kah Keng

Wong, Kelvin K.

Wong, Kwok‐Kin

Wong, Kwong‐Kwok

Wong, Martin

Wong, Martin C. S.

Wong, Mee‐Lian

Wong, Nathan

Wong, Ping Pui

Wong, Robert J.

Wong, Samuel Y. S.

Wong, Siu

Wong, Stephanie

Wong, Stephen T. C.

Wong, Vincent Wai‐Sun

Wood, Laura D.

Wood, Lori

Wood, Marie

Woods, Laura

Woods, Susan

Woodward, Aidan J.

Woong Won, Young

Woronoff, Anne‐Sophie

Worster, Brooke

Woudstra, Anke

Wright, Alexi

Wright, Frances

Wright, Graham

Wright, Jason

Wu, Kaijie

Wu, Ai‐Min

Wu, Anhua

Wu, Bin

Wu, Bogang

Wu, Chang‐Ping

Wu, Chen

Wu, Cheng‐Tien

Wu, Chen‐Jiang

Wu, Chia Chin

Wu, Chin‐Lee

Wu, Chunfu

Wu, Chun‐jiao

Wu, Chun‐Ying

Wu, Denglong

Wu, Di

Wu, Fan

Wu, Fang

Wu, Feixiang

Wu, Gang

Wu, Gaosong

Wu, Guan

Wu, Guoqiu

Wu, Hao

Wu, Hongxun

Wu, Hsi‐Chin

Wu, Hua

Wu, Jaw‐Ching

Wu, JF

Wu, Jianbing

Wu, Jianlin

Wu, Jianxiong

Wu, Jiayuan

Wu, Jiong

Wu, Liang

Wu, Li‐Ming

Wu, Ling

Wu, Min

Wu, Ming

Wu, Peihong

Wu, Qifei

Wu, Qiong

Wu, Roseann

Wu, San‐Gang

Wu, Sha

Wu, Shang‐Gin

Wu, Shiao‐Chi

Wu, Shixiu

Wu, Siqi

Wu, Susan

Wu, Teng

Wu, Tongwei

Wu, Tony

Wu, Victor Chien‐Chia

Wu, Wanhua

Wu, Wei

Wu, Wenchuan

Wu, Wen‐Chuan

Wu, Wendy

Wu, Wenfei

Wu, Xianpei

Wu, Xiaohua

Wu, Xifeng

Wu, Xiuqing

Wu, Xuan

Wu, Yaoyu

Wu, Yi‐Long

Wu, Yishuo

Wu, Yongyan

Wu, Yue

Wu, Yun

Wu, Zenghong

Wu, Ze‐Shen

Wu, Zhengyuan

Wu, Zhenyu

Wu, Zhi‐feng

Wullich, Bernd

Wun, Ted

Wunder, Jay

Wushouer, Aihemaiti

Wuu, Shenhong

Wyatt, A. W.

Wyatt, Alexander W.

Xavier, Joao

Xerri, Luc

Xi, Kexing

Xi, Lia

Xi, Mian

Xi, Xiaowei

Xi, Yong‐ming

Xia, Huan

Xia, Jian‐chuan

Xia, Jinglin

Xia, Leizhou

Xia, Liang

Xia, Liangping

Xia, Liang‐Ping

Xia, Lin‐Yu

Xia, Liqun

Xia, Peng

Xia, Ping

Xia, Qiang

Xia, Shuang

Xia, Tiansong

Xia, Wei

Xia, Weixiong

Xia, Yi

Xia, Yijun

Xia, Yong

Xia, Yujia

Xia, Zhangchuan

Xia, Zhijun

Xian, Junfang

Xiang, Cheng

Xiang, Fu

Xiang, Juanjuan

Xiang, Qiao

Xiang, Rong

Xiang, Ruo‐Lan

Xiang, Yan‐Qun

Xiao, Bingxiu

Xiao, Feifei

Xiao, Guodong

Xiao, Hongjun

Xiao, Liang

Xiao, Lingyan

Xiao, Weiwei

Xiao, Xinshu

Xiao, Xiu‐ying

Xiao, Yi

Xiao, Yu

Xiao, Yun

Xiao, Zefen

Xiao, Zhengrui

Xiao, Zhijian

Xiao, Zhiyu

Xie, Bowen

Xie, Chengrong

Xie, Conghua

Xie, Congying

Xie, Dan

Xie, Dong

Xie, Fang‐Yun

Xie, Haizhu

Xie, Jialing

Xie, Jingdun

Xie, Juan

Xie, Keping

Xie, Liang‐Xi

Xie, Liping

Xie, Peng

Xie, Qiang

Xie, Qing

Xie, Qi‐qi

Xie, Shang

Xie, Shuwei

Xie, Shu‐Yang

Xie, Tian

Xie, Wenbing

Xie, Xiaoming

Xie, Xiao‐ming

Xie, Xinhua

Xie, Yang

Xie, Yao

Xie, Yongzhi

Xie, Zhigang

Xie, Zhuoer Xie

Xing, Lei

Xing, Mingzhao

Xing, Puyuan

Xing, Qianwei

Xiong, Bin

Xiong, Gang

Xiong, Kun

Xiong, Momiao

Xiong, Wei

Xiong, Xiaoxing

Xiong, Zhenchong

Xu, Bilian

Xu, Bing‐he

Xu, Chongrui

Xu, Chuanliang

Xu, Chun‐wei

Xu, Dazhi

Xu, Feng

Xu, Guoping

Xu, Guoxiong

Xu, Hao

Xu, Hong

Xu, Hong‐Tao

Xu, Hua

Xu, Hui

Xu, Jianfeng

Xu, Jianhui

Xu, Jianming

Xu, Jiejie

Xu, Jiheng

Xu, Juan

Xu, Ke

Xu, L.

Xu, Lei

Xu, Lin

Xu, Ling

Xu, Mei‐Qing

Xu, Midie

Xu, Min

Xu, Ming

Xu, Ning

Xu, Ou

Xu, Pingping

Xu, Qian

Xu, Qingyong

Xu, Renfang

Xu, Rui‐Hua

Xu, Shaohua

Xu, Sheng

Xu, Shifen

Xu, Shuqin

Xu, Song

Xu, Teng

Xu, Tong‐Peng

Xu, W.

Xu, Wei

Xu, Weiguo

Xu, Wenrong

Xu, Wen‐Rong

Xu, Xianlin

Xu, Xiao

Xu, Xin‐Jian

Xu, Ying

Xu, Yinghua

Xu, Yingying

Xu, Yuzhen

Xu, Zekuan

Xu, Zhaomin

Xu, Zheng

Xu, Zhengshui

Xu, Zhijian

Xu, Zhijie

Xu, Zhonghua

Xue, Dong

Xue, Jianxin

Xue, Kangyi

Xue, Qiang

Xue, Ruiqi

Xue, Wanjiang

Xue, Wenrui

Xue, Xiangyang

Xue, Yuzhou

Yabroff, K. Robin

Yachida, Shinichi

Yagi, Asami

Yagi, Kenta

Yahalom, Joachim

Yamada, Kazutoshi

Yamada, Suguru

Yamada, Tadaaki

Yamada, Tesshi

Yamada, Yasutaka

Yamada, Yoshiya

Yamagami, Wataru

Yamaguchi, Hiroki

Yamaguchi, Motoko

Yamaguchi, Tatsuro

Yamamoto, Gou

Yamamoto, Keiko

Yamamoto, Masatsugu

Yamamoto, Masayuki

Yamamoto, Takaya

Yamasaki, Fumiyuki

Yamasaki, Toshinari

Yamashita, Hiroharu

Yamashita, Keishi

Yamashita, Tatsuya

Yamazaki, Shintaro

Yan, Jun

Yan, Caiwang

Yan, Cunling

Yan, Haiyan

Yan, Hua

Yan, Jinsong

Yan, Li

Yan, Lijun

Yan, Min

Yan, Pu

Yan, Rui

Yan, Taiqiang

Yan, Wang

Yan, Weigang

Yan, Xia

Yan, Yanhua

Yan, Yong

Yan, Yuanliang

Yanaga, Katsuhiko

Yanagawa, Masahiro

Yanagawa, Naoki

Yanagisawa, Takafumi

Yanagiya, Masahiro

Yanaihara, Nozomu

Yang, Ankui

Yang, Bikang

Yang, Bin

Yang, Bo

Yang, Chengliang

Yang, Chih‐Hsin

Yang, Da

Yang, Dae‐Sik

Yang, Da‐Jun

Yang, Daping

Yang, Dawei

Yang, Dong

Yang, Dong‐Hua

Yang, Eric

Yang, Fen

Yang, Fu

Yang, Gong

Yang, Grace Meijuan

Yang, GuoWang

Yang, Haidi

Yang, Hannah

Yang, Hong

Yang, Hongji

Yang, Hongjian

Yang, Hsin‐Ling

Yang, Hui

Yang, Huijun

Yang, Jilong

Yang, Jin

Yang, Jin‐Ji

Yang, Jinxiao

Yang, Jun

Yang, Jun‐Feng

Yang, Kehu

Yang, Keming

Yang, Kun

Yang, Lei

Yang, Li

Yang, Lian‐Yue

Yang, Lili

Yang, Limin

Yang, Lingge

Yang, Lin‐Jie

Yang, Liu

Yang, Min

Yang, Min‐fu

Yang, MingâYu

Yang, Pan‐Chyr

Yang, Qing

Yang, Qiwei

Yang, Richard

Yang, Shengyu

Yang, Shiming

Yang, Shun‐Fa

Yang, Si

Yang, Szu‐Chun

Yang, Tian

Yang, Tsai‐Sheng

Yang, Wanshui

Yang, Wei

Yang, Weina

Yang, Wenfang

Yang, Wenjuan

Yang, William

Yang, Wulin

Yang, Xi

Yang, Xia

Yang, Xiao

Yang, Xiaofeng

Yang, Xiaohang

Yang, Xiaohong

Yang, Xiaojun

Yang, Xiaokun

Yang, Xuelian

Yang, Yan

Yang, Yili

Yang, Ying

Yang, Yiping

Yang, Yu

Yang, Yungui

Yang, Yunpeng

Yang, Zhaozhi

Yang, Zhi

Yang, Zhiying

Yao, Yandan

Yao, Bowen

Yao, Hongliang

Yao, James

Yao, Jia

Yao, Jie

Yao, Ji‐Jin

Yao, Min

Yao, Ming

Yao, Shanglong

Yao, Yi‐Chen

Yao, Yingmin

Yao, Yongliang

Yao, Yongzhong

Yao, Yue

Yao, Zhenwei

Yao, Zhiqiang

Yardeni, Maya

Yared, Jean

Yarema, Roman

Yasaka, Koichiro

Yashiro, Masakazu

Yasuda, Koji

Yasuda, Masanori

Yasui, Hirofumi

Yasumatsu, Ryuji

Yates, Patsy

Yau, Thomas

Yauner, Del Pozo

Yavuz, Tunzala

Yaxley, John W.

Yaykasli, Kursat

Ye, Ding‐Wei

Ye, Fang‐Lei

Ye, Feng

Ye, Hua

Ye, Jian

Ye, Jing

Ye, Lihong

Ye, Mao

Ye, Shenglong

Ye, Ting‐Hong

Ye, Xinping

Ye, Yong'an

Ye, Yun

Ye, Zeng Jie

Ye, Zhaoming

Ye, Zhaoxiang

Yedjou, Clement

Yee Pin, Tan

Yee, Sook Wah

Yeh, Chao Hsing

Yeh, Chih‐Ching

Yeh, Chi‐Tai

Yeh, Edward T.H

Yeh, Kun‐Huei

Yeh, Kun‐Yun

Yeh, Norman

Yeh, Ying‐Chih

Yen, TC

Yen, Tina

Yen, Yi‐Ting

Yennurajalingam, Sriram

Yeoh, Allen Eng‐Juh

Yeom, Kristen

Yeung, Cecilia

Yeung, Sai‐Ching

Yeung, Wing‐Fai

Yi, Zhou

Yi, Gwan‐Su

Yi, Min

Yi, Ming

Yi, Shen

Yi, Yong

Yildirim, Fatma

Yilmaz, Atilla

Yilmaz, Emrullah

Yim, H. J.

Yin, Chonggao

Yin, Dong

Yin, Gang

Yin, Huiqing

Yin, Ivy

Yin, Jiandong

Yin, Ji‐Ye

Yin, Kai

Yin, Li

Yin, Lianghong

Yin, Lihong

Yin, Pei‐Hao

Yin, Rong

Yin, Sheng

Yin, Xiao‐Yu

Yin, Xin

Yin, Xinhai

Ying, H.

Ying, Hongmei

Ying, Hong‐mei

Ying, Hou‐Qun

Ying, Jianming

Ying, Zhitao

Yip, Desmond

Yip, Linwah

Yiu, Ming

Ynoe de Moraes, Fabio

Yock, Torunn

Yokota, Takaaki

Yokouchi, Hiroshi

Yokoyama, Minato

Yokozaki, Hiroshi

Yoldas, Tamer

Yomo, Shoji

Yong, Agnes

Yong, Kwee

Yong, Wei Peng

Yoo, Changhoon

Yoon, Dae Young

Yoon, Hyung‐Suk

Yoon, Jung Hyun

Yoon, Seong woo

Yoon, Sung‐Soo

Yoon, Ung‐Hwan

Yoon, WS

Yopp, Adam C.

Yoshida, Go

Yoshida, Kotaro

Yoshida, Takayuki

Yoshida, Tsutomu

Yoshida, Yoichiro

Yoshida, Yukio

Yoshihara, Kosuke

Yoshikawa, Takaki

Yoshimoto, Seiichi

Yoshino, Keiichi

Yoshino, Kiyoshi

Yoshino, Tadashi

Yosuico, Victor

You, Gan

You, Guoling

You, Hui

You, James

You, Jia

You, Kai‐Yun

You, Qidong

You, Wei

You, Yongping

You, Zhenhui

Youlden, Danny

Young, Kelvin

Young, Ken

Young, Ken He

Young, Lawrence S.

Young, Teresa

Yousry, Tarek

Youyong, Lu

Yozgat, Ahmet

Ysebaert, Loic

Yu, Baozhong

Yu, Chaohui

Yu, Cheng‐Ping

Yu, Chunjiang

Yu, Chun‐Ping

Yu, Dandan

Yu, Dianke

Yu, Evan

Yu, Gordon

Yu, Hongping

Yu, Hsiang‐Hsuan

Yu, Hua

Yu, Hui

Yu, Huiming

Yu, Irene

Yu, J.

Yu, James

Yu, Jianxiu

Yu, Jingru

Yu, Jinming

Yu, Keda

Yu, Ke‐Da

Yu, Kenneth

Yu, Kexun

Yu, Kun

Yu, Lan

Yu, Lijuan

Yu, Lin

Yu, Miao

Yu, Ming

Yu, Mingxia

Yu, Ming‐Xia

Yu, Qi

Yu, Qiangfeng

Yu, Qingzhao

Yu, Qiong

Yu, Rao

Yu, Ri‐Sheng

Yu, Shan

Yu, Shitong

Yu, Shu‐Han

Yu, Su Jong

Yu, Wei

Yu, Xianjun

Yu, Xiaobing

Yu, Xue

Yu, Xue Qin

Yu, Yan

Yu, Yang

Yu, Yingyan

Yu, Yiqun

Yu, Yongfeng

Yu, Yuming

Yu, Zhi Gang

Yu, Zhi‐Ling

Yu, Zhiyong

Yu, Zhuang

Yuan, Chen

Yuan, Jianlin

Yuan, Jian‐Min

Yuan, Jiansong

Yuan, Jia‐qi

Yuan, Lei

Yuan, NaiJun

Yuan, Peng

Yuan, Sheng‐Tao

Yuan, Shuang‐Hu

Yuan, Taize

Yuan, Xianglin

Yuan, Yan

Yuan, Ying

Yuan, Yuan

Yuan, Yun‐Fei

Yuan, Zheng

Yuan, Zhi‐Yong

Yuan, Zou

Yue, Jinbo

Yue, Junming

Yue, Ning

Yue, Zhixia

Yue, Zhiyong

Yuen, Carlen

Yun, Jing‐Ping

Yun, Young Ho

Yurgelun, Matthew B.

Yurkiewicz, Ilana

Yusoff, Muhamad Saiful Bahri

Yussif, Shaimaa M.

Yusuf, Mehran

Z. Kornblith, Lucy

Zabihollahy, Fatemeh

Zachary, Iris

Zafar, Mohammad Ishraq

Zafar, Yousuf

Zagouri, Flora

Zahnd, Whitney

Zakharia, Yousef

Zalaudek, Iris

Zalcman, Gerard

Zamagni, Elena

Zamani‐Ahmadmahmud, Mohamad

Zamarin, Dmitriy

Zamponi, Gerald

Zanardi, Elisa

Zandi, Ashkan

Zang, Rongyu

Zang, Xingxing

Zangari, Maurizio

Zaninotto, Giovanni

Zannad, Faiez

Zaorsky, Nicholas

Zaorsky, Nicholas G.

Zapardiel, Ignacio

Zappulli, Valentina

Zaravinos, Apostolos

Zarif, Talal

Zatelli, Maria Chiara

Zattra, Ottavia

Zdravkovic, Marko

Zebrack, Brad

Zehir, Ahmet

Zeidan, Amer M.

Zeidner, Joshua

Zeng, Bing

Zeng, Bo

Zeng, Hao

Zeng, Jian

Zeng, Jiang‐hui

Zeng, Jin

Zeng, Jinhua

Zeng, Lingfeng

Zeng, Lingliao

Zeng, Musheng

Zeng, Mu‐Sheng

Zeng, Xian‐Tao

Zeng, Xian‐tao

Zeng, Xiaomin

Zeng, Yi

Zeng, Yingchun

Zeng, Yong

Zeng, Yongji

Zeng, Zhili

Zeng, Zhuoying

Zenke, Yoshitaka

Zent, Clive S.

Zepeda, Rossana

Zeraati, Hojjat

Zerini, Dario

Zeuner, A.

Zhai, Guangtao

Zhan, Cheng

Zhan, Chuanchuan

Zhan, Siyan

Zhan, Xinli

Zhang, Rui

Zhang, Ben

Zhang, Bin

Zhang, Bixiang

Zhang, Bo

Zhang, Boxin

Zhang, Caiguo

Zhang, Chenan

Zhang, Chengkai

Zhang, Chenlin

Zhang, Chen‐Yu

Zhang, Chi

Zhang, Chi‐Hao

Zhang, Chuan‐bao

Zhang, Chunlin

Zhang, Chunlong

Zhang, Cuifeng

Zhang, Cuiting

Zhang, Dahong

Zhang, Diancai

Zhang, Dongkun

Zhang, Donglu

Zhang, Dongyu

Zhang, Erbao

Zhang, Fan

Zhang, Guihai

Zhang, Guo‐Jun

Zhang, Guo‐Nan

Zhang, Guoxin

Zhang, Haifang

Zhang, Hailiang

Zhang, Hai‐Liang

Zhang, Hanbin

Zhang, Hanqun

Zhang, Hao

Zhang, Hongliang

Zhang, Hong‐Liang

Zhang, Hong‐Mei

Zhang, Hongpan

Zhang, Hongtao

Zhang, Hong‐Tao

Zhang, Hongxia

Zhang, Hongyi

Zhang, Hua

Zhang, Huafeng

Zhang, Huan

Zhang, Hui

Zhang, Hui Lai

Zhang, Huojun

Zhang, Jian

Zhang, Jianguo

Zhang, Jianning

Zhang, Jianying

Zhang, Jiaqi

Zhang, Jiarui

Zhang, Jihong

Zhang, Jin

Zhang, Jinfang

Zhang, Jing

Zhang, Jingdong

Zhang, Jingyao

Zhang, Jing‐yu

Zhang, Jing‐Zhe

Zhang, Jun

Zhang, Kai

Zhang, Lan

Zhang, Lanjing

Zhang, Lei

Zhang, Leiyi

Zhang, Li

Zhang, Lichao

Zhang, Liling

Zhang, Lin

Zhang, Lingyun

Zhang, Linyou

Zhang, Liping

Zhang, Liqun

Zhang, Liwei

Zhang, Lu

Zhang, Mei

Zhang, Meng

Zhang, Mengping

Zhang, Mengqi

Zhang, Mengxian

Zhang, Milan

Zhang, Ming

Zhang, Mingxin

Zhang, Nancy R.

Zhang, Nu

Zhang, Nuo

Zhang, Qi

Zhang, Qin

Zhang, Qing

Zhang, Qingyong

Zhang, Qingyu

Zhang, Qiuping

Zhang, Ronggui

Zhang, Rongyu

Zhang, Shengqiang

Zhang, Shuishen

Zhang, Shuisheng

Zhang, Shutian

Zhang, Shuyu

Zhang, Si‐He

Zhang, Sina

Zhang, Songling

Zhang, Tao

Zhang, Ti

Zhang, Tian

Zhang, Tingyou

Zhang, Wei

Zhang, Weijuan

Zhang, Weiying

Zhang, Wen‐Wen

Zhang, Wenxiong

Zhang, Xianbin

Zhang, Xiang

Zhang, Xiao

Zhang, Xiaochun

Zhang, Xiaohua Douglas

Zhang, Xiaojing

Zhang, Xiaoshi

Zhang, Xiaotao

Zhang, Xiaowen

Zhang, Xiaoyang

Zhang, Xiao‐Yun

Zhang, Xiaozhi

Zhang, Xipeng

Zhang, Xuchao

Zhang, Xue

Zhang, Xuebin

Zhang, Xuehong

Zhang, Xuening

Zhang, Xuepei

Zhang, Ya

Zhang, Yafang

Zhang, Yan

Zhang, Yanmei

Zhang, Yaodong

Zhang, Yao‐Jun

Zhang, Yi

Zhang, Yilei

Zhang, Yimu

Zhang, Yingshi

Zhang, Yinjie

Zhang, Yiteng

Zhang, Yiwen

Zhang, Yiyin

Zhang, Yonghong

Zhang, Yuan

Zhang, Yue

Zhang, Zedan

Zhang, Zhang

Zhang, Zhe

Zhang, Zhen

Zhang, Zheng

Zhang, Zhengdong

Zhang, Zhenwen

Zhang, Zhigang

Zhang, Zhi‐Jiang

Zhang, Zhimin

Zhang, Zhiqiang

Zhang, Z‐J

Zhao, An

Zhao, Bin

Zhao, Chengliang

Zhao, Chengquan

Zhao, Chong

Zhao, D.

Zhao, Dakun

Zhao, Dongbing

Zhao, Fangqing

Zhao, Fangzheng

Zhao, Feng

Zhao, Gang

Zhao, Haitao

Zhao, Hengqiang

Zhao, Hui

Zhao, Jiangman

Zhao, Jie

Zhao, Jingjing

Zhao, Jingxuan

Zhao, Jizong

Zhao, Jun

Zhao, Jun‐Wei

Zhao, Kuaile

Zhao, Lan

Zhao, Lin

Zhao, Liwei

Zhao, Lujun

Zhao, Ming

Zhao, Na

Zhao, Qian

Zhao, Qingyuan

Zhao, Shuang

Zhao, Shujun

Zhao, Song

Zhao, Wei

Zhao, Wei‐Li

Zhao, Xiao‐Su

Zhao, Yan

Zhao, Yang

Zhao, Yashuang

Zhao, Yong

Zhao, Yongzhao

Zhao, Yu

Zhao, Zhenyu

Zhao, Ziran

Zhao, Zongmao

Zhen, Jianzhuo

Zhen, Weining

Zhen, Yong‐Su

Zheng, Binglin

Zheng, Chun‐Hou

Zheng, Guoqiao

Zheng, Jie

Zheng, Junfang

Zheng, Limin

Zheng, Liwei

Zheng, Ming‐Hua

Zheng, Mingjun

Zheng, Qiusheng

Zheng, Ronghui

Zheng, Rongshou

Zheng, Shangyong

Zheng, Shuohan

Zheng, Shu‐Sen

Zheng, Wei

Zheng, Xiangqian

Zheng, Xiao

Zheng, Xinhua

Zheng, Yan

Zheng, Yijie

Zheng, Yuanlin

Zheng, Yuan‐Lin

Zheng, Yuansheng

Zheng, Yun

Zhi, W.

Zhi, Xiuling

Zhong, Guo‐Chao

Zhong, Jian‐Hong

Zhong, Judy

Zhong, Miaochun

Zhong, Rong

Zhong, Tian

Zhong, Wenzhao

Zhong, Wen‐Zhao

Zhong, Yucheng

Zhong, Yue

Zhong, Yun

Zhong, Zhengxiang

Zhongliu, Wei

Zhou, Jian‐Guo

Zhou, Baosen

Zhou, Binggang

Zhou, Bingrong

Zhou, Binhua (Peter)

Zhou, Caicun

Zhou, Can

Zhou, Chengmao

Zhou, Chengzhi

Zhou, DaoBin

Zhou, Fan

Zhou, Fang‐Jian

zhou, Fuling

Zhou, Guanqun

Zhou, Haiyu

Zhou, Haojie

Zhou, Hebing

Zhou, He‐Bing

Zhou, Hongfei

Zhou, Huaqiang

Zhou, Hui

Zhou, Huixia

Zhou, J.

Zhou, Jian

Zhou, Jian‐Guo

Zhou, Jian‐Hui

Zhou, Jian‐Ping

Zhou, Jianqiao

Zhou, Jianya

Zhou, Jianyin

Zhou, Jifang

Zhou, Jing

Zhou, Juan

Zhou, Lei

Zhou, Li

Zhou, Liang

Zhou, Liqun

Zhou, Maigeng

Zhou, Meng

Zhou, Qi

Zhou, Qiang

Zhou, Qing

Zhou, Qingyu

Zhou, Quan

Zhou, Shengtao

Zhou, Songwen

Zhou, Ting

Zhou, Wei

Zhou, Wei‐ping

Zhou, WeiWen

Zhou, Wenbin

Zhou, Wence

Zhou, Wuyuan

Zhou, Xiang

Zhou, Xiaodong

Zhou, Xifa

Zhou, Xiong‐Hui

Zhou, Yan‐Ming

Zhou, Yiming

Zhou, Ying

Zhou, Yong

Zhou, Yongying

Zhou, Youlang

Zhou, Yuan

Zhou, Zhengyang

Zhou, Zhiaing

Zhou, Zhihua

Zhou, ZiYuan

Zhu, Andrew

Zhu, Bo

Zhu, Chen

Zhu, Dingjun

Zhu, Dong

Zhu, Fan

Zhu, Feng‐Cai

Zhu, Guangli

Zhu, Guopei

Zhu, Haihong

Zhu, Haiyan

Zhu, Hao

Zhu, Hengcheng

Zhu, Hongbo

Zhu, Hui

Zhu, Jie

Zhu, Jinhong

Zhu, Jinshui

Zhu, Jun

Zhu, Kun

Zhu, Li

Zhu, Liancheng

Zhu, Lingjun

Zhu, Liucun

Zhu, Lizhe

Zhu, Ma

Zhu, Meng

Zhu, Shijie

Zhu, Shikai

Zhu, Shuchai

Zhu, Ting

Zhu, Tong

Zhu, Wan

Zhu, Weiwen

Zhu, Xi

Zhu, Xiao

Zhu, Xiaoliang

Zhu, Xiaolu

Zhu, Xiaonian

Zhu, Xin

Zhu, Xingen

Zhu, Xueqiong

Zhu, Yanjie

Zhu, Ya‐Yun

Zhu, Yazhen

Zhu, Ya‐Zhen

Zhu, Yi

Zhu, Yi

Zhu, Yi‐Ping

Zhu, Yueyong

Zhu, Zhaowei

Zhu, Zhengfei

Zhu, Zheng‐Jiang

Zhuang, Guohong

Zhuang, QianFeng

Zhuang, Xiaodong

Zhuang, Zhixiang

Ziebland, Sue

Ziebolz, Dirk

Zielinski, Christina E.

Ziemlewicz, Timothy J.

Ziglioli, F.

Zikmund‐Fisher, Brian

Zimmer, Jacques

Zimmer, Lisa

Zimmermann, Tanja

Zingone, Fabiana

Zingue, Stephane

Zinzani, Pier Luigi

Zito, Emanuela

Zixing, Chen

Zlotoff, Dan

Zlotta, Alexandre R.

Znaor, Ariana

Zoccarato, Marco

Zokaasadi, Mohammad

Zöller, Bengt

Zong, Liang

Zong, Xiangyun

Zong, Zhi‐Hong

Zongwei, Huang

Zorzi, Manuel

Zoschke, Christian

Zou, RuHai

Zou, Weiping

Zou, Wenbo

Zou, Xi

Zou, Yuan Yuan

Zou, Zhengzhi

Zribi, Kaouther

Zu, Youli

Zubatkina, Irina

Zucca, Emanuele

Zucca, Emanuelle

Zugmaier, Gerhard

Zukauskaite, Ruta

Zumsteg, Zachary

Zuna, Rosemary

Zuo, Shuguang

Zuo, Ying

Zureikat, Amer

Zurita, Amado

Zuur, Lotje

Zylla, Dylan

Zyoud, Sa'ed

